# Neuronal ageing is promoted by the decay of the microtubule cytoskeleton

**DOI:** 10.1371/journal.pbio.3002504

**Published:** 2024-03-13

**Authors:** Pilar Okenve-Ramos, Rory Gosling, Monika Chojnowska-Monga, Kriti Gupta, Samuel Shields, Haifa Alhadyian, Ceryce Collie, Emilia Gregory, Natalia Sanchez-Soriano

**Affiliations:** Department of Biochemistry, Cell and Systems Biology, Institute of Systems, Molecular & Integrative Biology, University of Liverpool, Liverpool, United Kingdom; MRC Laboratory of Molecular Biology, UNITED KINGDOM

## Abstract

Natural ageing is accompanied by a decline in motor, sensory, and cognitive functions, all impacting quality of life. Ageing is also the predominant risk factor for many neurodegenerative diseases, including Parkinson’s disease and Alzheimer’s disease. We need to therefore gain a better understanding of the cellular and physiological processes underlying age-related neuronal decay. However, gaining this understanding is a slow process due to the large amount of time required to age mammalian or vertebrate animal models. Here, we introduce a new cellular model within the *Drosophila* brain, in which we report classical ageing hallmarks previously observed in the primate brain. These hallmarks include axonal swellings, cytoskeletal decay, a reduction in axonal calibre, and morphological changes arising at synaptic terminals. In the fly brain, these changes begin to occur within a few weeks, ideal to study the underlying mechanisms of ageing. We discovered that the decay of the neuronal microtubule (MT) cytoskeleton precedes the onset of other ageing hallmarks. We showed that the MT-binding factors Tau, EB1, and Shot/MACF1, are necessary for MT maintenance in axons and synapses, and that their functional loss during ageing triggers MT bundle decay, followed by a decline in axons and synaptic terminals. Furthermore, genetic manipulations that improve MT networks slowed down the onset of neuronal ageing hallmarks and confer aged specimens the ability to outperform age-matched controls. Our work suggests that MT networks are a key lesion site in ageing neurons and therefore the MT cytoskeleton offers a promising target to improve neuronal decay in advanced age.

## Abstract 2

Aging is associated with structural and functional changes in neurons thought to drive motor, sensory, and cognitive impairments. Using a Drosophila model of aging, the authors report that deterioration of the microtubule cytoskeleton is an important driver of neuronal aging.

## Introduction

Normal physiological ageing conveys a decline in sensory, motor, and cognitive functions, with deterioration accelerating in the last decade of life. For example, short-term memory shows a slow deterioration with increasing age, which accelerates dramatically after the age of 70 years old. Similarly, weakening of sensory functions, such as hearing and vision, becomes widespread amongst individuals over 80 years of age [1–5]. The deterioration of these functions is characterised by behavioural changes and deficits. For example, the loss of sensory input is linked to a decline in social and physical activities leading to progressive social isolation, affecting activities of daily living and quality of life [6].

The key factors which cause an age-related decline in brain function and cognitive behaviour are not well understood. However, a picture is emerging that suggests this is not due to widespread cell death [7,8]. Instead, distinct age-related alterations of neuronal structures, such as synapses, axons, and dendrites, are proposed to be responsible for the extensive volumetric loss of brain tissue found in humans during ageing [2,9]. Examples of such alterations in the mammalian brain include the progressive occurrence of atrophic axons, typically characterised by irregular membrane profiles and swellings [10]. Peripheral mouse motor axons display a marked decrease in axonal diameter with age [11–13]. Additionally, in axons comprising the murine retina, varicosities, and reduced synaptic content are observed upon age [14]. Synaptic structures also display morphological changes during ageing. For example, in the rat hippocampus and mouse cerebellum, synapses develop discrete membrane swellings or oedemas, and exhibit a decrease in active zones [15–17]. Understanding the causes behind these structural changes will be pivotal to ameliorate the time-dependent decline of the nervous system.

A promising starting point is the microtubule (MT) cytoskeleton, given it is an essential component of neurons, and in particular, axons. MTs are arranged into parallel bundles that run uninterrupted throughout axons. MTs form molecular highways to facilitate for life-sustaining transport, required for most biological processes in axons, and serve essential roles in driving morphogenetic processes [18–20]. However, maintaining MT bundles long-term is a demanding task given the continued mechanical challenges MTs are subjected to [21–24]. Accordingly, MT density and organisation are reported to be altered in ageing axons of humans and other primates [10,25].

Considering the unique dependency of cellular functions on MTs, we asked whether MT deterioration may be a cause for axon decay, rather than a secondary consequence. The regulation of MTs in vivo requires the function of MT-binding proteins (MTBPs), which regulate MT nucleation, polymerisation, disassembly, stabilisation and cross-linkage, posttranslational modification, and repair [26–29]. An imbalance in this complex regulatory network of MTBPs could explain MT decay and neuronal atrophy reported during ageing [23,25,30–32]. We propose that gaining a better understanding of MT regulation and its roles during axonal maintenance and ageing could provide a promising path to gain new insights into age-related neuronal decay and unveil new therapeutic strategies.

To overcome the high complexity of axonal cell biology and MT regulatory mechanisms, we used the model organism *Drosophila melanogaster*. Importantly, *Drosophila* shows clear signs of severe ageing within a matter of a few weeks, including diminished stress resistance, changes in behaviour, altered metabolism, reduced barrier function in the gut, and compromised cardiac function [33]. Ageing has also been reported to impact *Drosophila* neurons, including age-related changes in mitochondrial dynamics, synaptic content, and diminished neuronal function [34–40].

Using this model, we performed a detailed characterisation of neuronal ageing in cells situated within the fly visual system. We observed prominent ageing hallmarks, remarkably similar to those observed in mammals, including axonal swellings, altered axon diameters, and cytoskeletal and synaptic decay. The earliest ageing phenotypes was MT bundle decay: discontinuity, disturbances in the bundle organisation and loss of MT volume. Based on their key functions in axonal development in *Drosophila* neurons, we selected 3 MTBPs to further investigate their possible role in axonal maintenance: Tau, the spectraplakin Short stop (Shot) and end-binding protein 1 (EB1). Both the MT lattice-binding factor Tau and Shot regulate MT stability in developing neurons. The MT plus end-binding factor EB1 works as a scaffold protein mediating MT plus-end binding of multiple MTBPs. In addition to this, it is known that together Tau, Shot and EB1 provide guidance to polymerising MTs in growing axons [29,41–45]. Here, we found that genetic reduction of these MT regulators induced ageing-like MT defects and exacerbated age-dependent neuronal decay. Moreover, by performing genetic manipulations known to stabilise and enhance MT networks, neurons were protected from developing hallmarks of ageing. As a result, a causative link between MT deregulation and neuronal decay was demonstrated.

## Results

### Age alters the morphology of axons and synaptic terminals

**Fig 1:**
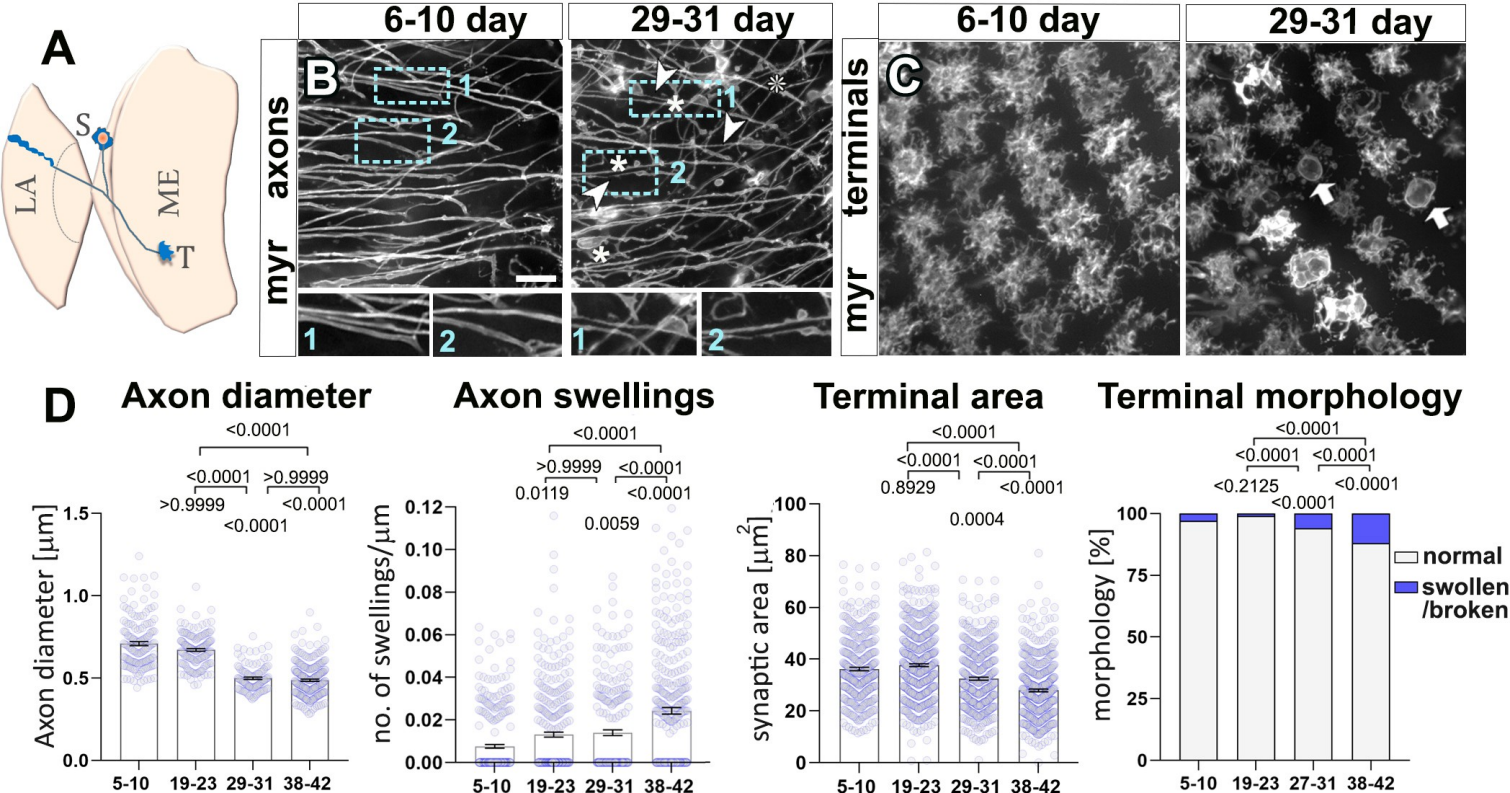
Axons and synaptic terminals within the *Drosophila* visual system deteriorate during ageing. (**A**) Representation of an optic lobe region of the adult *Drosophila* brain depicting a soma (S) of a T1 neuron which has a projection that bifurcates terminating in the medulla (ME; T, terminals analysed in this study) and in the lamina (LA). (**B, C**) Axons (B) and terminals (C) of the T1 medulla projections from young (6–10 days) and old specimens (29–31 days) labelled with the plasma membrane marker myr-Tom (myr). In aged specimens, axons show thinning (arrow heads) and swellings (asterisks; dashed blue boxes shown as 1.7-fold magnified images below), and axonal terminals appear broken down and with swellings (arrows) and overall reduced area. (**D**) Quantifications of phenotypes shown in B and C, with age groups in DAE, indicated on the X-axes. In the left 3 graphs, data points are shown in lilac and as mean ± SEM. *P*-values obtained via Kruskall–Wallis ANOVA multiple comparison tests are indicated in the graphs. For terminal morphology, data are represented as distribution of normal versus swollen/broken synapses; significance obtained via Chi-square test is indicated above. Data were taken from a minimum of 10 specimens, 100 axons or 300 terminals per age group. For detailed statistical values and genotypes, see Table A within the S1 Tables. All the single values are provided in the S1 Datapoints. Scale bar in B represents 10 μm in B and 14 μm in C. DAE, days after eclosure.

To understand how axons and synaptic terminals are changing with age and what can be driving this process, we set out to establish an in vivo cellular model which would allow us to study the cell biology of ageing. We focused on the *Drosophila* visual system, in particular, on T1 neurons which are second order interneurons in the medulla rind (Fig 1A). T1 neurons form projections that bifurcate into 1 branch arborising in layer M2 of the medulla where they contact several synaptic partners, and another projecting into the lamina [46,47] (Fig 1A). Several properties make the medulla-localised axons of T1 neurons ideal for subcellular studies: they are large calibre axons with characteristic synaptic terminals in stereotyped medulla locations; they are easy to image and manipulate due to their proximity to the surface of the tissue; and numerous genetic tools are available to facilitate their effective visualisation and manipulation.

To study the medulla axons of T1 neurons, we labelled the membrane via targeted expression of the fluorescently tagged membrane marker myristoylated-Tomato (myr-Tom) driven by the *GMR31F10-Gal4* line [48]. Labelled axons were visualised via an ex vivo live imaging method (see Materials and methods). We performed comparative analyses between specimens of different ages. Since the range of temperatures at which *Drosophila* can be reared is variable and higher temperatures facilitate faster specimen ageing, we opted to age *Drosophila* at 29°C, a temperature that is ecologically relevant and allows faster experimentation [49]. Under these conditions, we found that the diameter of T1 axons decreased compared to young axons, their membranes become irregular, and they develop frequent axonal swellings (Fig 1B). These changes were significant in flies aged over 29 days after eclosure (DAE) (Fig 1D). Other neurons within the visual system also undergo similar age-related changes in axons, as shown in L2 lamina neurons (S1 Fig), suggesting that these alterations are not T1-dependent and may be widespread across other *Drosophila* neuronal cell types.

In addition to morphological changes in the shaft of axons, we found that ageing altered the morphology of axonal terminals containing synapses. In young specimens, T1 terminals in layer M2 of the medulla neuropile consist of hand-shaped, dense arborisations (Fig 1A and 1C). In old specimens (starting at approximately 30 days old), these arborisations become irregular and less compact, resulting in an overall decrease in the area of the terminal, and in extreme cases, apparent fragmentation of the synaptic membranes (Fig 1C and 1D). Additionally, synaptic terminals appear swollen, adopting a highly spherical and varicose shape (arrows in Fig 1C and 1D). These changes occur in the absence of neuronal death, as suggested by quantification of T1 neuron nuclei labelled with RedStinger [50] (S2 Fig). These ageing alterations occurring in *Drosophila* show similarities to transformations which occur in the murine retina, motor axons, and the primate brain, which all display axonal varicosities and reduced axonal terminals with age [10–12,14].

To demonstrate that these alterations were not a consequence of long-term expression of the exogenous membrane marker myr-Tom, we used the conditional expression system Gal4/Gal80^ts^ [51,52] (see Materials and methods) that enabled us to restrict expression of the membrane marker to equal short time periods in both young and old flies. This system relies on the ubiquitous expression of the transcriptional repressor Gal80, which contains a temperature-sensitive mutation that renders it inactive at 29°C. Thus, at 29°C, Gal80 is inactivated, allowing Gal4 to freely induce transcription of UAS-dependent transgenes, whereas in flies reared at 18°C, expression of the transgene is repressed by active Gal80. Since rearing flies at 18°C delays development, we aged flies for either 53 to 56 (old) or 0 to 3 (young) days at 18°C in the absence of myr-Tom (marker expression is suppressed at 18°C), then incubated both young and old flies at 29°C for 4 days to enable short-term expression of myr-Tom, before dissecting and imaging the brains (S3 Fig). We found that axons from old specimens displayed: reduced diameters, irregular membrane profiles, increased swellings, and altered synaptic morphology, when compared to young ones (S3E Fig). This indicated that the defects observed using this model were a consequence of ageing and not of long-term stress due to continuous prolonged myr-Tom expression. These results further confirm that significant ageing alterations found in axonal and synaptic terminals are independent of the temperature *Drosophila* is reared at.

Taken together, our data demonstrated that significant structural changes occur in both axonal and synaptic compartments during ageing. Aged neurons display a decrease in the diameter of axons that develop swellings in addition to alterations in the morphology of synaptic terminals, suggesting that axonal and synaptic integrity are compromised.

### Changes in the MT cytoskeleton precede axonal and synaptic decay during ageing

The MT cytoskeleton is a key determinant of cell shape and of the morphology of axons and synaptic terminals [53–55]. MTs are also suggested to be key factors in regulating the calibre of axons, especially in conditions of low abundance or absence of neurofilaments [19,56–58]. We therefore hypothesised that MTs can play a key role in the context of age-related neuronal defects observed with our model. To this end, we first investigated whether ageing acutely affects the MT cytoskeleton of neurons.

**Fig 2:**
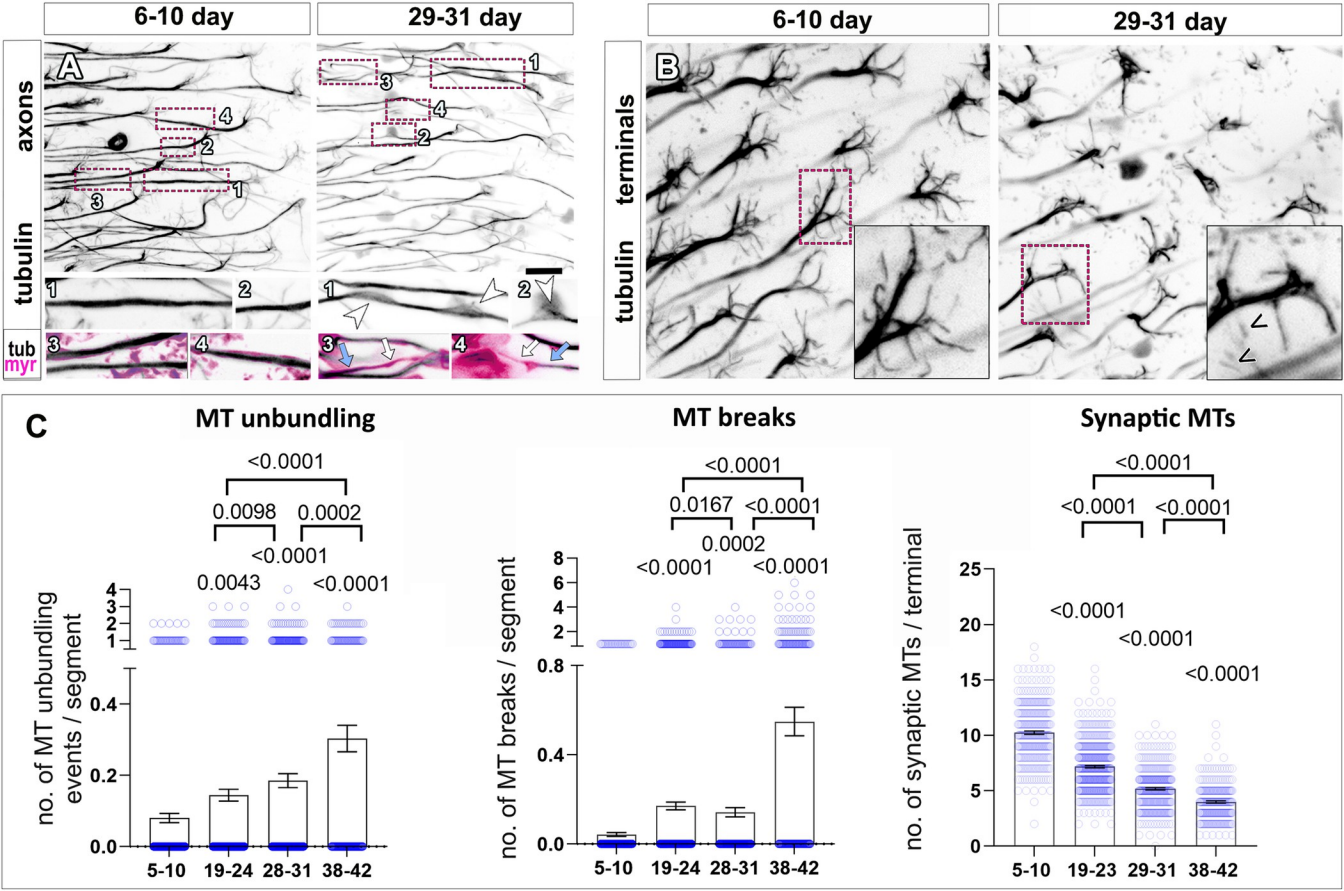
MT aberration precedes axonal and synaptic decay during ageing. (**A, B**) T1 neuron axons (A) and synaptic terminals (B) in the medulla region of young (6–10 days) or old (29–31 days) flies and labelled with GFP-tagged α-tubulin (tubulin or tub. in inverted greyscale images for easier visualisation), and the plasma membrane marker myr-Tom (myr, magenta in insets). In A, magenta encircled boxes are shown 2.3-fold magnified below with old flies showing MT unbundling (arrowheads, insets 1 and 2 in A) as well as breaks on MT bundles (white arrows, insets 3 and 4 in A) compared to uninterrupted stretches of MTs in in the same axon (blue arrows) or in young flies. In B, old flies also show a reduction of splayed MTs in synaptic terminals (insets) and MTs which appeared fragmented (chevrons in inset in B). (**C**) Quantifications of phenotypes shown in A and B, with age groups indicated below X-axes; data points are shown as blue circles and as mean bars ± SEM; *p*-values obtained via Kruskall–Wallis ANOVA multiple comparison tests are indicated above. Data were taken from a minimum of 17 specimens per age group. For detailed statistical values and genotypes, see table D within the S1 Tables. All the single values are provided in the S1 Datapoints. Scale bar in A represents 10 μm in A and 5 μm in B. MT, microtubule.

To visualise axonal and synaptic MTs in T1 neurons, we expressed GFP-tagged α-tubulin (tubulin::GFP, Grieder and colleagues). As anticipated, axonal MTs of T1 neurons in young adults are arranged in a prominent, continuous, and uniform bundle along the axon, which splays into multiple thinner bundle branches at the synaptic terminal (left panels, Fig 2A and 2B). However, in older specimens, we observed that axonal MTs lose their tight, parallel arrangements, deviating from their bundles leading to disorganised foci (“MT unbundling”). Moreover, there were regions within axons which appeared to contain thinner MT bundles and, in extreme examples, appeared devoid of MTs (“MT breaks”) (right panels, Figs 2A, 2C and S4 for further examples at higher magnification). We confirmed the age-dependent overall thinning of axonal MT bundles using high-end confocal microscopy, as well as stimulated emission depletion (STED) super-resolution microscopy (S5A and S5B Fig). By measuring the cross-section of MT bundles within T1 axons (as an indication of the bundle diameters), we found a significant decrease in old versus young specimens when imaged with both techniques (S5C and S5D Fig). In parallel, we also observed that the splayed synaptic MTs often appeared fragmented and decreased in number with age (over 50% reduction from 38 days; Fig 2B and 2C). An assessment of the ageing phenotypes at multiple time points revealed that axonal and synaptic MT bundle deterioration is already prominent from 19 days onwards, suggesting that MT defects occur prior to the onset of morphological changes, e.g., thinning of axons, and synaptic alterations (detectable not before about 30 days; Fig 2C versus Fig 1D).

In order to discard any confounding contributions to such phenotypes due to long-term expression of tubulin::GFP, we utilised the same approach as carried out for myrTom: the Gal4/Gal80^ts^ system was used to control temporal expression of tubulin-GFP for an equal amount of days in young and old specimens. Under these conditions of time-controlled expression, aged specimens still developed the same age-related MT phenotypes (S6 Fig), thus ruling out long-term expression of tubulin-GFP as the cause of the described ageing phenotypes.

**Fig 3:**
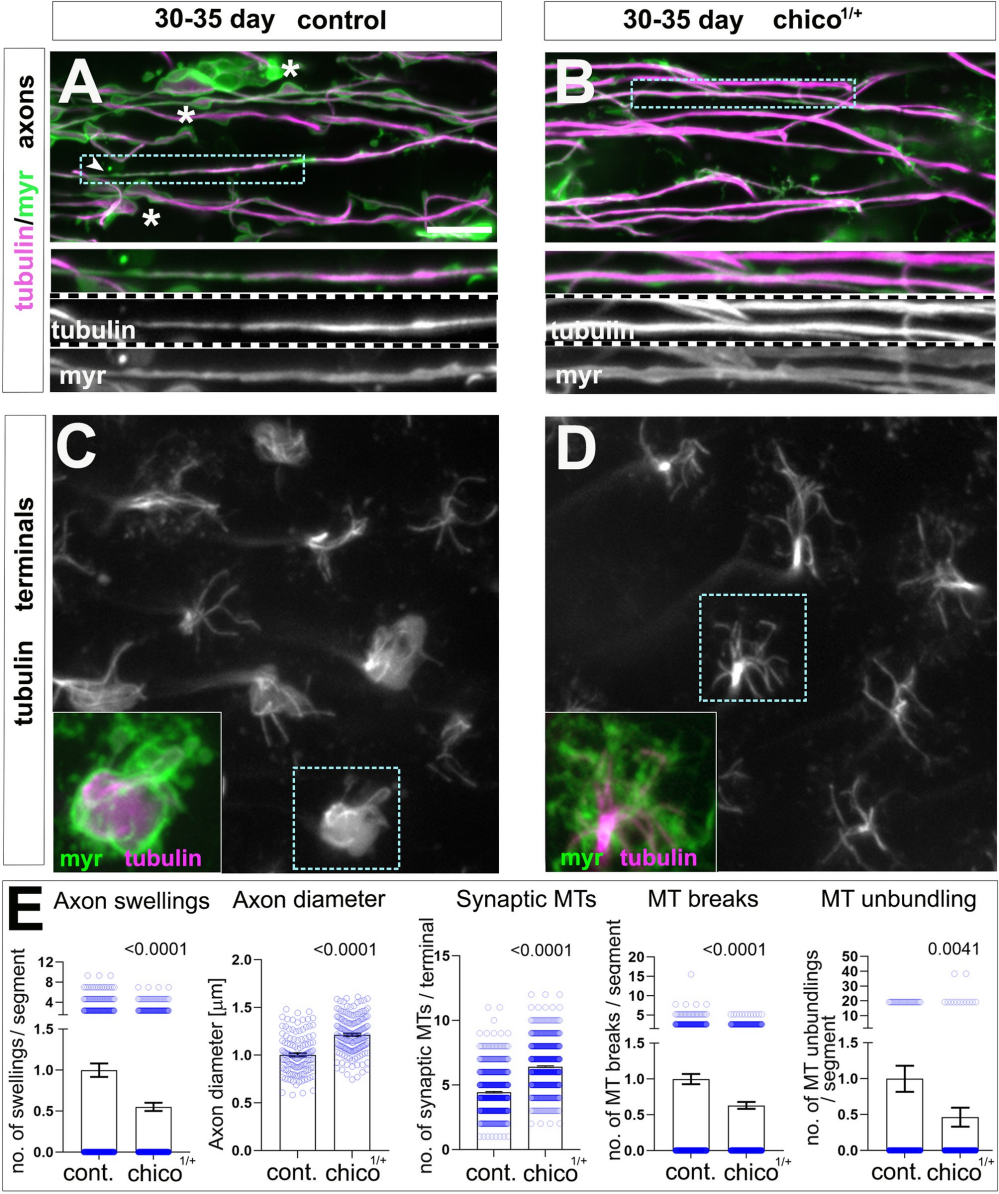
A mutation in the insulin receptor substrate CHICO improves neuronal ageing hallmarks and MT decay. (**A–D**) T1 axons (top) and synaptic terminals (bottom) in the medulla of old specimens of 30–35 days are labelled with GFP-tagged α-tubulin (tubulin, magenta in A and B and greyscale in C and D) and the plasma membrane marker myr-Tom (myr, green in A and B). Ageing phenotypes in old specimens in A include axon swellings (asterisks), axon thinning (arrow heads), and MT breaks/thinning (boxed area shown as 2-fold magnified inset); as well as reduction of splayed MTs in synaptic terminals shown in C (boxed areas shown as 2-fold magnified insets). *chico*^*1*^ heterozygous in B and D, suppresses phenotypes in ageing neurons in aged-matched specimens. (**E**) Quantifications of phenotypes shown in A–D, in the absence or presence of *chico*^*1/+*^, indicated below X-axes; data points are shown as blue circles and as mean ± SEM; *p*-values obtained via Mann–Whitney test are indicated above. Data were taken from a minimum of 16 specimens per group. For detailed statistical values and genotypes, see Table F within the S1 Tables. All the single values are provided in the S1 Datapoints. Scale bar in A represents 10 μm in A and B, and 6 μm in C and D. MT, microtubule.

To further validate whether the observed phenotypes are consequences of ageing, we combined our cellular model with the insulin/IGF signalling pathway which is a well-studied longevity pathway known to modulate the rate of ageing in a variety of organisms [59]. We utilised the *chico*^*1*^ allele, which is a mutation in the insulin receptor substrate CHICO that increases lifespan in heterozygotes up to 36% [60]. We reasoned that lifespan-improving factors that modulate ageing and improve the overall fitness of the organisms would better the health of different tissues, including the nervous system. T1 neurons in the *chico*^*1*^ heterozygotes background improved all measured ageing hallmarks in old flies, including swellings and axonal thinning, as well as MT deterioration in the axons and synaptic terminals (Fig 3).

Altogether, our results suggest that the hallmarks reported here are the consequence of physiological ageing. Furthermore, MT bundle deterioration occurs before the onset of morphological changes, suggesting that MT bundle deterioration is an important event implicated in axon and synaptic decay observed during ageing.

### Tubulin incorporation to axonal MTs decreases with age

The thinning of axonal MT bundles and the decrease in synaptic MTs we observed in old specimens may suggest that mechanisms mediating the maintenance of MT density in axons are compromised with age. Such mechanisms could include MT nucleation, polymerisation, and stabilisation within axons, which have been the subject of multiple studies conducted primarily in developing axons [26–29]. However, to our knowledge, very little is known about MT replenishment and maintenance in mature axons which can be expected to be an important mechanism to counteract processes such as the wear and tear, imposed for example by motor proteins (see Introduction).

**Fig 4:**
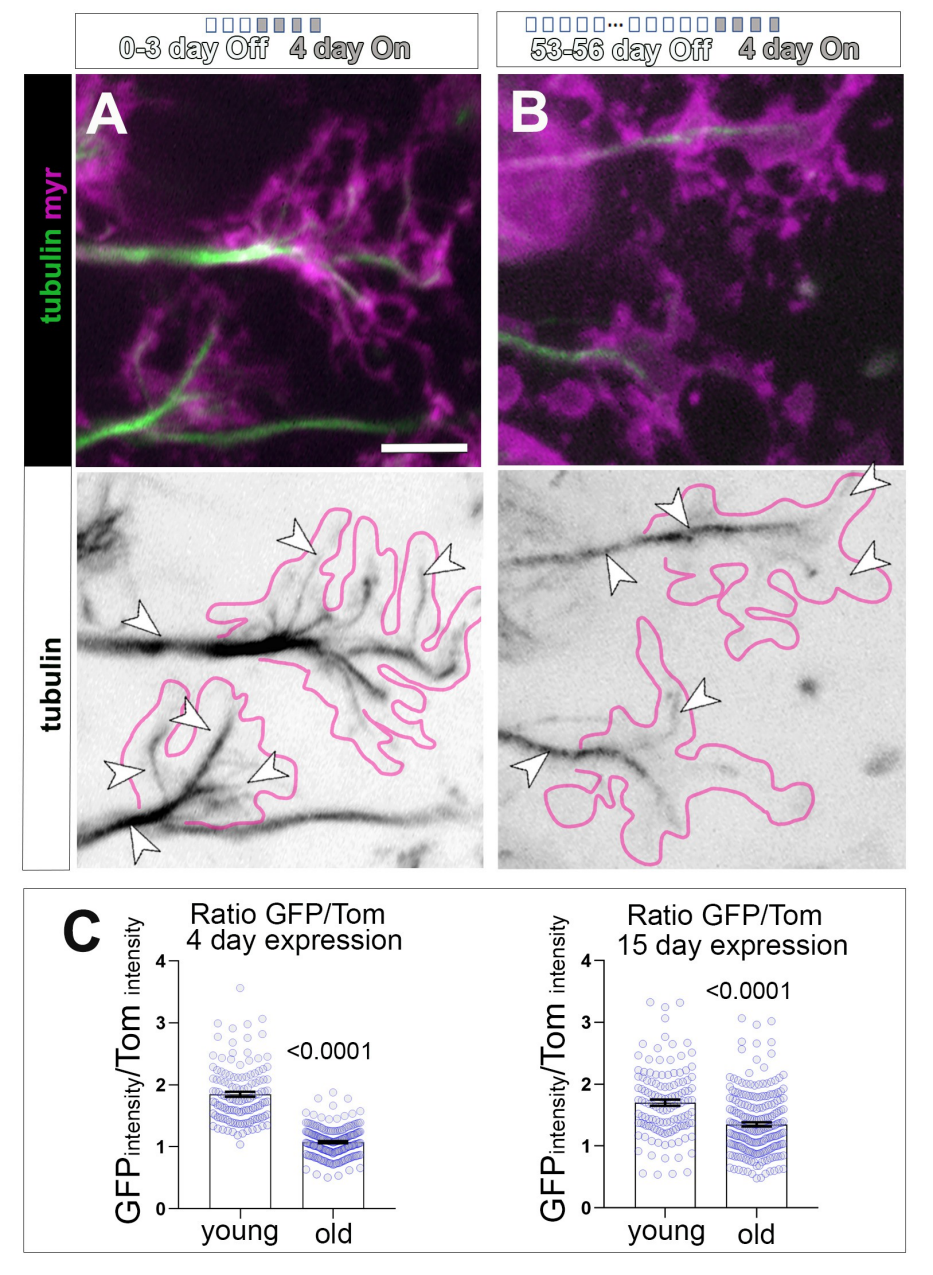
Decreased presence of tubulin-GFP at axonal MTs in a pulse chase experiment suggests changes in MT replenishment with age. (**A, B**) T1 axonal terminals in the medulla of flies expressing GFP-tagged α-tubulin (tubulin, in green and in inverted greyscale image) and the plasma membrane marker myr-Tom (myr, magenta), using the UAS/Gal4/Gal80^ts^ system. The induction of gene expression in the adult brains is restricted to 4 days by the shift of temperature from 18 to 29°C. For this, flies were kept at 18°C throughout development and adult life until the last 4 days before imaging, at which point they were shifted to 29°C to initiate gene expression. Young specimens (A; 4–7 days old flies with 0–3 days at 18°C “Off” + 4 days at 29°C “On”) are compared to old specimens (B; 57–60 days old flies with 53–56 days at 18°C “Off” + 4 days at 29°C “On”). Tub-GFP can be observed incorporated within MTs (arrowheads) in axons and axonal terminals (indicated by the magenta outline) with very low cytosolic signal. (C) Quantifications of the ratio between GFP-tub and myr-Tom (Ratio GFP/Tom) in 2 experimental conditions (under 4 days or 15 days expression regime), with young versus old specimens indicated on the X-axes; data points are shown as blue circles and as mean ± SEM; *p*-values obtained via Mann–Whitney test are indicated above. Individual data are taken from at least 5 or 6 specimens per age group for 4 or 15 days expression, respectively. For detailed statistical values and genotypes, see Table G within the S1 Tables. All the single values are provided in the S1 Datapoints. Scale bar can be found in A bottom right and corresponds to 5 μm. MT, microtubule.

To investigate MT replenishment and maintenance in mature neurons and whether they are affected by age, we performed a pulse-chase experiment in which tubulin::GFP was expressed during a restricted period of 4 days in either young or old specimens, after which we examined to what degree *GMR31F10-Gal4*-driven tubulin::GFP was incorporated into the axonal MT bundle. To achieve this, we utilised the conditional Gal4/Gal80^ts^ expression system: young and old flies were maintained at 18°C (tubulin::GFP expression is inactive) until being moved to 29°C for 4 days (tubulin::GFP expression is active). Young flies were moved to 29°C at days 0 to 3 of age. Old flies were moved at days 53 to 56 of age. In both young and old specimens, tubulin::GFP was incorporated within axonal and synaptic MTs, indicating that MTs continue to be replenished in adult T1 neurons at both time points (Fig 4A and 4B). To assess the level of tubulin::GFP incorporation, we quantified the intensity of GFP in axonal MT bundles, and to exclude potential artefacts due to changes in driver activity, we normalised tubulin::GFP to co-expressed myr-Tom. These analyses revealed a significant decrease in GFP intensity in axons from old flies when compared to the younger group (Fig 4C, left graph). Even in conditions where the pulse of tubulin expression had been increased to 15 days, we observed a significant decrease in tubulin::GFP incorporated to MTs (Fig 4C, right graph).

These data strongly suggest that MT replenishment and maintenance is diminished during ageing, which could explain the deterioration of the axonal MT bundle we observed in ageing flies.

### MT-binding activities of EB1 and Tau decrease with age

Next, we set out to investigate the mechanisms that could lead to the decrease in MT integrity and replenishment with age. EB1 and Tau are potential candidates responsible for the maintenance of MT bundles. EB1 is a scaffold protein preferentially localising to polymerising MT plus ends where it mediates the binding of factors involved in polymerisation dynamics and the guidance of extending MTs into organised bundles [29,41–44]. Tau stabilises MTs along their lattice, and it promotes MT polymerisation by maintaining EB1 at extending MT plus ends [43,61,62]. Although loss of endogenous *Drosophila* Tau does not affect lifespan [63], the aberrant localisation of Tau is a hallmark of ageing in the human brain and correlates with memory dysfunction [64,65].

**Fig 5:**
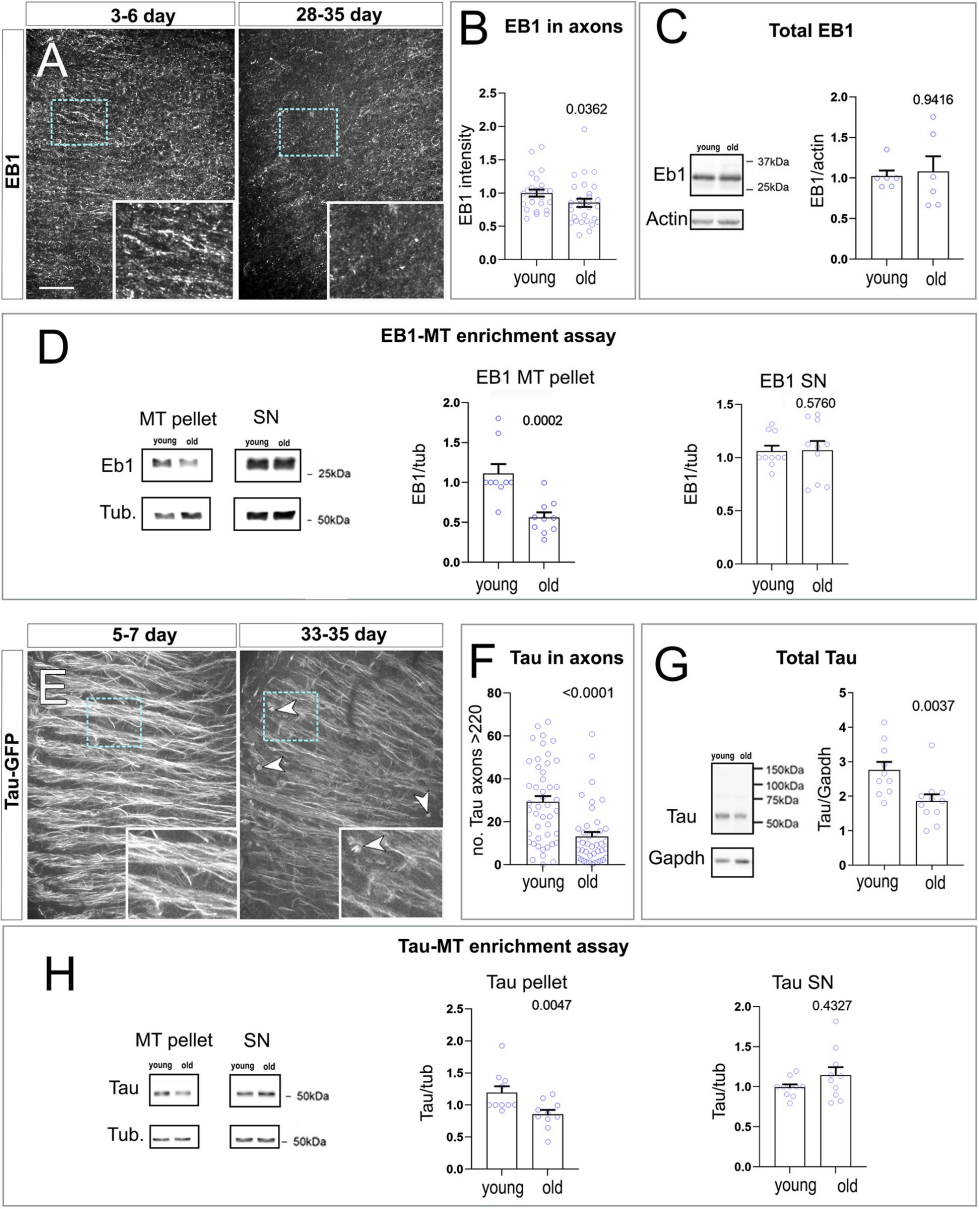
The function of EB1 and Tau is altered during ageing. (**A**) Axonal region of the medulla of young (3–6 days, at 29°C) and old (28–35 days, at 29°C) flies labelled with anti-EB1. (**E**) Axonal region of a medulla labelled with endogenous GFP-tagged Tau (carrying the *tau*^*wee304/+*^ allele) at 2 different time points (young 5–7 and old 33–35 days old, both aged at 29°C). In aged specimens, axons show a decrease in EB1 and GFP-Tau (dashed cyan boxes in A and E are shown as 2- and 2.3-fold magnified insets, respectively. Arrowheads in E indicate aggregate-like structures). Images as in A and E are used for (**B**) quantifications of EB1 signal intensity and (**F**) of the number of axons with high levels of GFP-Tau (above 220 units of intensity). Age categories, as explained above, are indicated below X-axes. Data points represent medullas and are shown as blue circles and as mean ± SEM; *p*-values obtained by Mann–Whitney test are indicated above. Data were taken from a minimum of 17 specimens per age group. (**C** and **G**) Total EB1 and Tau by western blot reveals a decrease in Tau during ageing, while the level of EB1 remains constant. Age categories are indicated below the X-axes (4–9 and 29–32 for C and 8–10 and 29–31 for G). Data points represent independent lysates and are shown as blue circles and as mean ± SEM; *p*-values obtained by Mann–Whitney test are indicated above. (**D** and **H**) MT-binding assays were performed using head extracts from young (4–6 days old) and old (28–31 days old) specimens, followed by fractionation to obtain a MT-enriched pellet and a soluble SN. Both the MT and SN fraction were probed with anti-EB1, anti-Tau, and anti-Tubulin. Data points represent independent MT-binding assays and are shown as blue circles and as mean ± SEM; *p*-values obtained by Mann–Whitney test are indicated above. Quantifications confirm that the amount of EB1 and Tau bound to MTs decreases with age. For detailed statistical values and genotypes, see Table H within the S1 Tables. All the single values are provided in the S1 Datapoints and all western blots can be found in the S1 Western Blots. Scale bar in A represents 20 μm in A and E. MT, microtubule; SN, supernatant.

We started by investigating age-dependent changes in EB1 and Tau localisation in whole brains. In the medulla of young specimens, we found that EB1 antibody staining appeared as distinct speckles throughout the brain tissue, with regions of short, continued strokes (Fig 5A) within areas containing T1 axonal MTs (S7B Fig). Such patterns of expression likely represent EB1 bound to the plus end and/or discrete regions along MT lattices as previously observed [24,43]. Notably, EB1 axonal localisation is weaker in brains dissected from old flies, as suggested by a significant reduction in EB1 intensity in axonal regions within the medulla (Fig 5A and 5B).

Tau antibody staining proved to be unreliable in whole brains. As an alternative strategy, we used the viable *tau*^*wee304*^ allele which contains a GFP inserted into its genomic locus [66]. In young adult *tau*^*wee304/+*^ specimens, Tau^wee304^ was predominantly localised to axons within the medulla, exhibiting a filamentous-like pattern, consistent with binding along MT lattices (Fig 5E). Colocalisation of Tau to MTs was further demonstrated with the MT probe Sirt-Tubulin (S7A Fig). However, this pattern changed in aged medullas that displayed a significant decrease in Tau^wee304^ GFP intensity in axons (Fig 5E and 5F). In addition, we observed the appearance of aggregate-like structures (Fig 5E, arrowheads). This relocalisation of *Drosophila* Tau shows similarities to alterations observed in aged human individuals or upon certain tauopathies including Alzheimer’s disease [67,68].

Reduced axonal association of EB1 and Tau in ageing neurons may reflect an overall decrease in their levels. To test this hypothesis, we quantified Tau and EB1 protein levels in extracts from *Drosophila* heads isolated at different ages. We found that the level of EB1 remained unaffected in old specimens (Fig 5C), while there was a decrease of total Tau in old specimens (Fig 5G). The decrease in Tau could be due to either decrease in expression or a sequestration of Tau in aggregates which may have not been detected in our western blot studies.

To further understand whether the MT regulatory function of Tau and EB1 may be altered during ageing, we compared the MT-binding properties of these factors in adult young and old brains. For this, we performed a standard MT-binding spin-down assay using extracts from fly heads at different ages [69]. We found that the amount of EB1 and Tau detected in the MT-enriched fraction from old brains significantly decreased when compared to young counterparts (Fig 5D and 5H), implying that there is less EB1 and Tau bound to MTs in old specimens. This suggests that the ability of EB1 to bind MTs decreases with age and is consistent with the reduced EB1 intensity we observed in aged axons, while the axonal Tau loss in aged brains may be due to both diminished MT binding and a global decrease in protein levels in the old brain.

### Loss of MT regulators exacerbates ageing hallmarks

**Fig 6:**
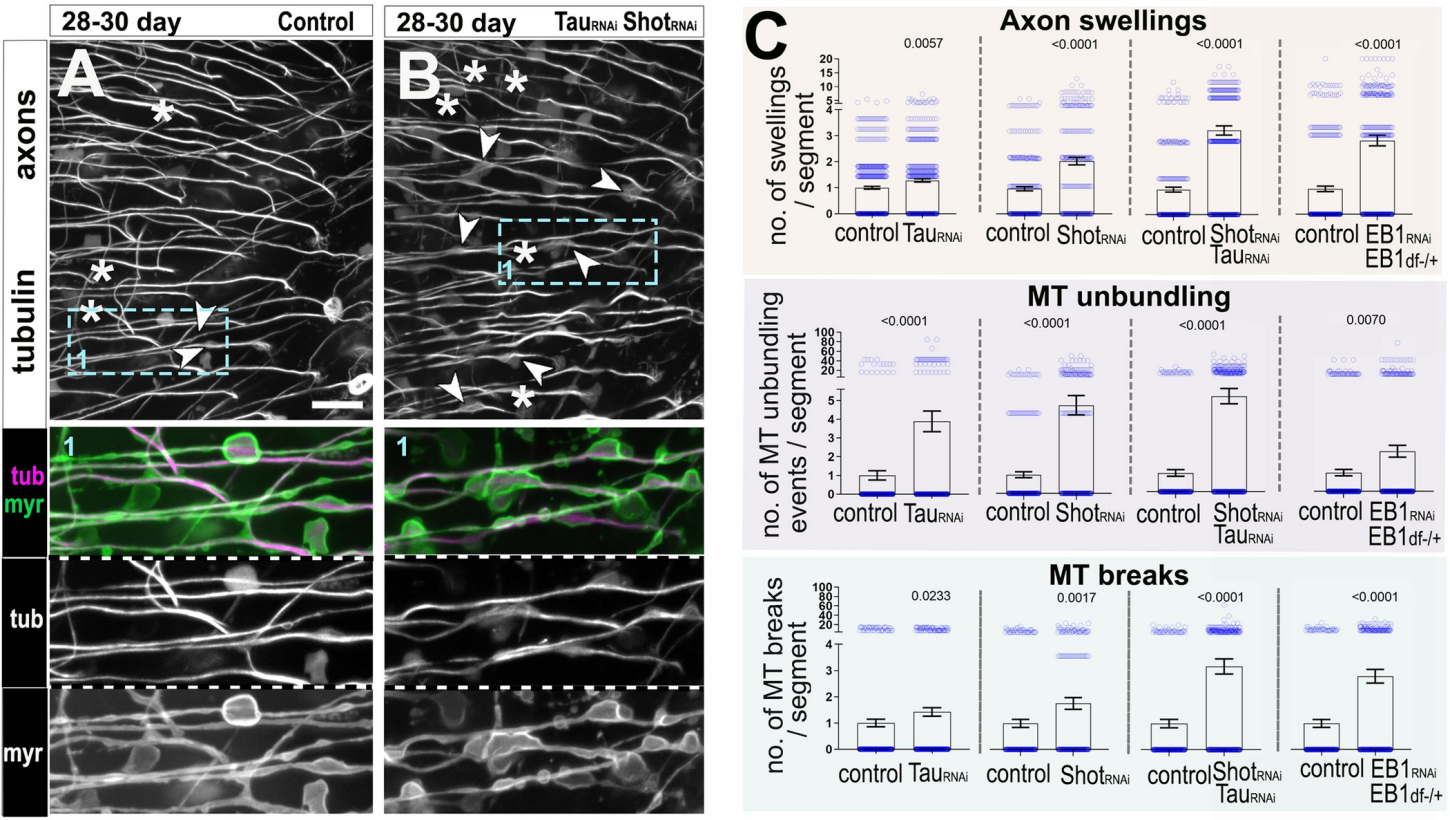
Knocking down Eb1, Tau, and Shot exacerbates age-related MT decay and ageing hallmarks. (**A, B**) Representative images of T1 axons in the medulla of 28–30 old flies labelled with GFP-tagged α-tubulin (tubulin, greyscale, and magenta in insets) and the plasma membrane marker myr-Tom (myr, green in insets). Aged neurons in the absence (A) or presence of combined Tau and Shot knockdowns (*Tau*_*RNAi*_ *Shot*_*RNAi*_ in B) are compared. Combined Tau and Shot knockdowns enhance phenotypes in ageing neurons, comprising axon swellings often displaying MT unbundling (arrow heads) and MT breaks (asterisks); boxed areas shown as 2-fold magnified double/single-channel images below. (**C**) Quantifications of phenotypes shown in A and B plus conditions of further individual knockdowns for Tau (*Tau*_*RNAi*_), Shot (*Shot*_*RNAi*_), and EB1 in an EB1 heterozygote background (*EB1*_*RNAi*_*; EB1*^*+/*Df^); specific knockdowns are indicated below the X-axes. Data points are shown as blue circles and as mean ± SEM; *p*-values obtained via Mann–Whitney tests are indicated above. Data were taken from a minimum of 14 specimens per age group and condition. For detailed statistical values and genotypes, see Table I within the S1 Tables. All the single values are provided in the S1 Datapoints. Scale bar in A represents 10 μm in all images. MT, microtubule.

Our results might suggest that an age-related decrease in the function and/or abundance of key MT regulators could be the cause of MT loss and unbundling, and even of the subsequent axonal and synaptic decay that occurs in the ageing process. To assess this possibility, we performed targeted expression in T1 neurons of previously validated RNAi constructs against several essential MT regulators: Tau, EB1 as well as the *Drosophila* spectraplakin Shot as a further essential MT regulator [29,41]. T1-specific knock-down of Tau in old specimens compared to age-matched controls resulted in a significant increase in the number of sites where MT bundles were disturbed or displayed breaks. These MT phenotypes correlated with a rise in the number of axonal swellings (Figs 6C, S8A and S8B).

Tau and Shot were shown previously to redundantly regulate MT stability in developing neurons [62]. In addition, Shot provides guidance of MT growth [41]. We therefore carried out T1-specific knock-down of *shot* either alone or in double knock-down with *tau*, and found a similar increase in MT phenotypes and axonal swellings as observed upon knock-down of *tau* alone, with MT breaks and axonal swellings being clearly enhanced upon double knock-down (Figs 6, S8A and S8C). We extended our analysis to synaptic terminals, revealing a clear exacerbation of deficits in the number of synaptic MTs and an increase in swollen and broken synapses in T1-specific double knock-down (S9A, S9C and S9E Fig).

EB1 long half-life has been reported before [41]. To overcome this in our studies, we performed T1-specific knock-down of *EB1* in an *EB1* heterozygous mutant background (using an allele carrying a deficiency of the *EB1* gene *Df(2R)Exel6065*). This condition led to an increase in MT breaks, areas of MT disorganisation and the development of axonal swellings in aged axons (Figs 6C, S8A and S8D).

None of the knock-down experiments induced MT breaks and unbundling/disorganisation or axonal swellings in young specimens (S10 and S11 Figs) suggesting MT phenotypes and axonal swellings are promoted by age in adult specimens.

Taken together, we show that the loss of MT regulator activities such as Tau, Shot, and EB1 exacerbate MT phenotypes in aged neurons, suggesting these factors are contributing to the maintenance of axonal MT bundles. Furthermore, their deficient function greatly exacerbates age-associated axonal and synaptic atrophy, suggesting that they are likely factors implicated in the normal process of neuronal ageing.

### Protecting and enhancing MT bundles ameliorates axonal ageing phenotypes

The combination of MT bundle deterioration and a reduction of key MT regulators to MTs (Fig 5), together with their loss of function phenotypes (Figs 6, S8 and S9) and the late onset of non-MT-related axonal phenotypes (Figs 1 and 2), suggest that aberrant MT regulation is a major cause for axonal decay during ageing. We therefore reasoned that we may be able to slow down age-related axonal atrophy by artificially boosting the function of specific MT regulators to protect MTs from decay during ageing.

**Fig 7:**
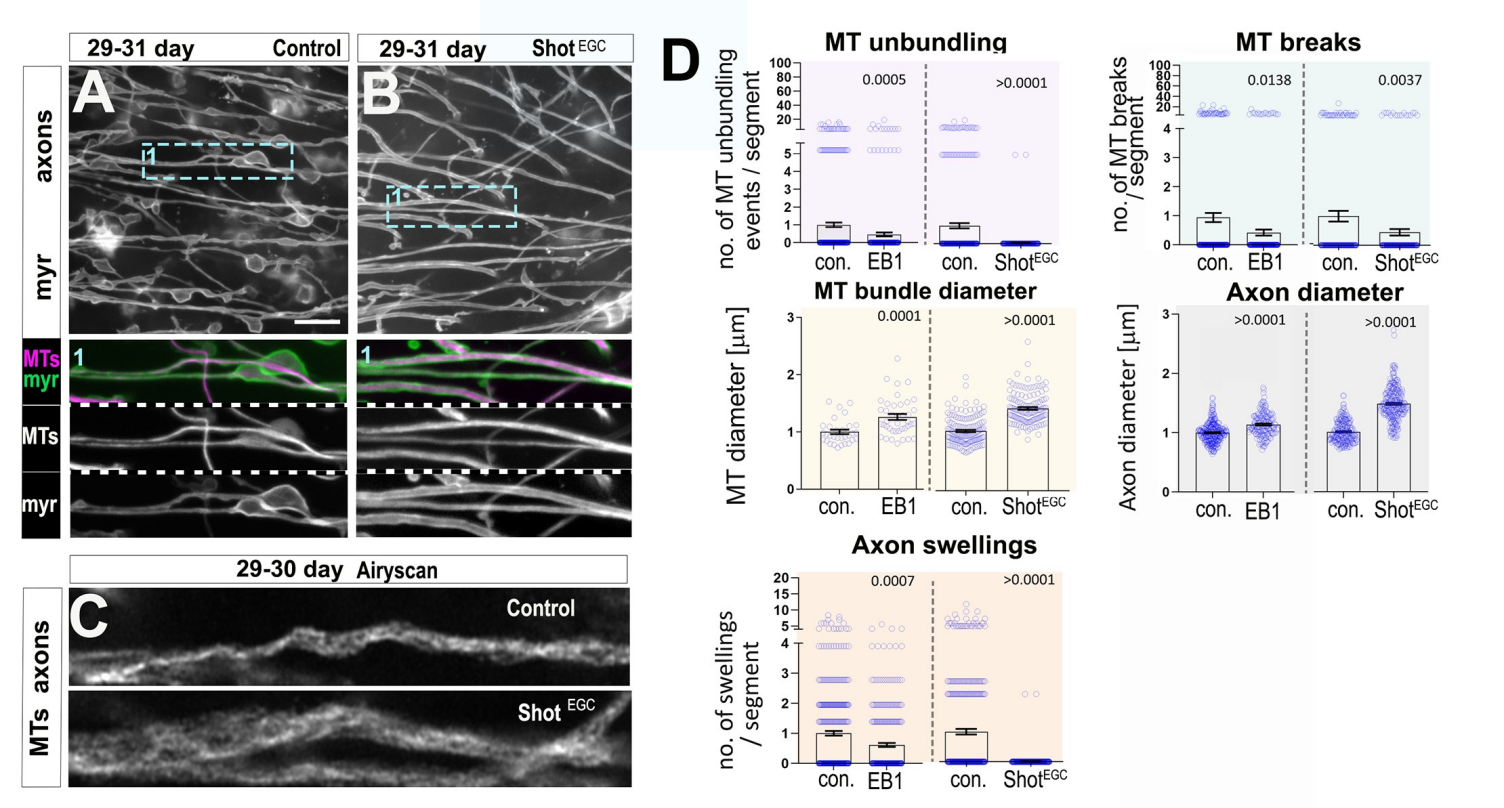
Expression of EB1 and of Shot^EGC^ ameliorates axonal ageing phenotypes. (**A, B**) T1 axons in the medulla of old specimens of 29–31 days old are labelled with the plasma membrane marker myr-Tom (myr, greyscale in A and B, and green in double-channel images). Ageing phenotypes including axon swellings, axon thinning, and MT unbundling can be observed in old specimens in A, but are absent upon T1-specific expression of Shot^EGC^ encoding the C-terminus of Shot (A compared to B, boxed area 1 shown as 2-fold magnified inset where MTs are labelled with GFP-tagged α-tubulin in A or GFP-tagged Shot^EGC^ in B). (**C**) MT axonal bundles from T1 neurons from old specimens (29–30 days old, labelled with GFP-tagged α-tubulin or GFP-tagged Shot^EGC^), imaged using Airyscanning high-end confocal microscopy, appear thicker upon Shot^EGC^ expression. (**D**) Quantifications of phenotypes shown in A–C, plus conditions of EB1 ectopic expression. Specific conditions are indicated below X-axes. Data points are shown as blue circles and as mean ± SEM; *p*-values obtained via Mann–Whitney test are indicated above. Data were taken from a minimum of 13 specimens per group (except for diameter measures of MT bundle with a minimum of 6, and axons with minimum of 10 specimens). For detailed statistical values and genotypes, see Table L within the S1 Tables. All the single values are provided in the S1 Datapoints. Scale bar in A represents 10 μm in A and B, and 2 μm in C. MT, microtubule.

Counteracting the loss of Tau from MTs by overexpressing Tau might be counterproductive as suggested by detrimental outcomes of this strategy in *Drosophila* neurons [70]. Alternatively, we overexpressed EB1 in T1 neurons and examined the ageing-associated phenotypes at 4 to 5 weeks. We observed an improvement across all tested ageing phenotypes (Figs 7D, S9E and S13), including reduced disorganisation and breaks of axonal MT bundles, in addition to an increase in the MT bundle diameters and quantity of MTs at the synaptic terminals. Moreover, EB1 overexpression rescues non-MT-related phenotypes, including a reduction in axonal swellings, an increase in axonal calibres, and a rejuvenated morphology of synaptic terminals in old specimens.

Next, we tested another approach to decelerate age-related axonal atrophy, by expressing the C-terminus of Shot fused to GFP (Shot^EGC^::GFP) as an alternative to protect MTs from deterioration during ageing. Shot^EGC^ contains the Gas2-related domain/GRD, which is weakly associated with MT lattices and protects them against depolymerisation, and the C-tail, which shows modest association with MT lattices and contains EB1-binding SxIP motives. Combined in the Shot^EGC^::GFP construct, these domains mediate strong association all along MT lattices, fully decorate MT bundles protecting them against depolymerisation and facilitate an association with EB1 [29,41]. Consistent with our previous work in primary neurons and fibroblasts [41], we find that Shot^EGC^::GFP also decorates axonal MTs in T1 neurons (S12 Fig). Similar to EB1 overexpression, Shot^EGC^::GFP primarily improved deteriorating MTs and substantially improved all examined ageing phenotypes in axons, including axonal swellings and thinner axons (Fig 7A–7D), and in synaptic terminals (S9B, S9D and S9E Fig). In summary, strengthening MT networks via EB1::GFP and Shot^EGC^::GFP overexpression is sufficient to rescue age-related phenotypes in T1 neurons.

When Shot^EGC^ was strictly overexpressed in T1 mature neurons in the adult brain using the Gal4/Gal80^ts^ system, all tested age-related MT phenotypes, together with axonal swellings, were significantly improved. Overexpressing EB1 in adult T1 neurons, on the other hand, significantly rescues axonal swellings, with weaker improvement on MTs breaks and unbundling (S14 Fig). This data suggests that Shot^EGC^ is sufficient to block MT and axonal decay in adult brains, while the protective effects mediated by EB1 during ageing may require expression at an early developmental stage to fully rescue MT atrophy.

These findings further support our hypothesis that MT decay can be an important cause for axon and synaptic decay during ageing. They further highlight the prospect of employing MT regulation as a target for potential therapeutic intervention to combat the deterioration of neurons during ageing.

### Shot^EGC^ and EB1 expression improves the motor performance of flies

**Fig 8:**
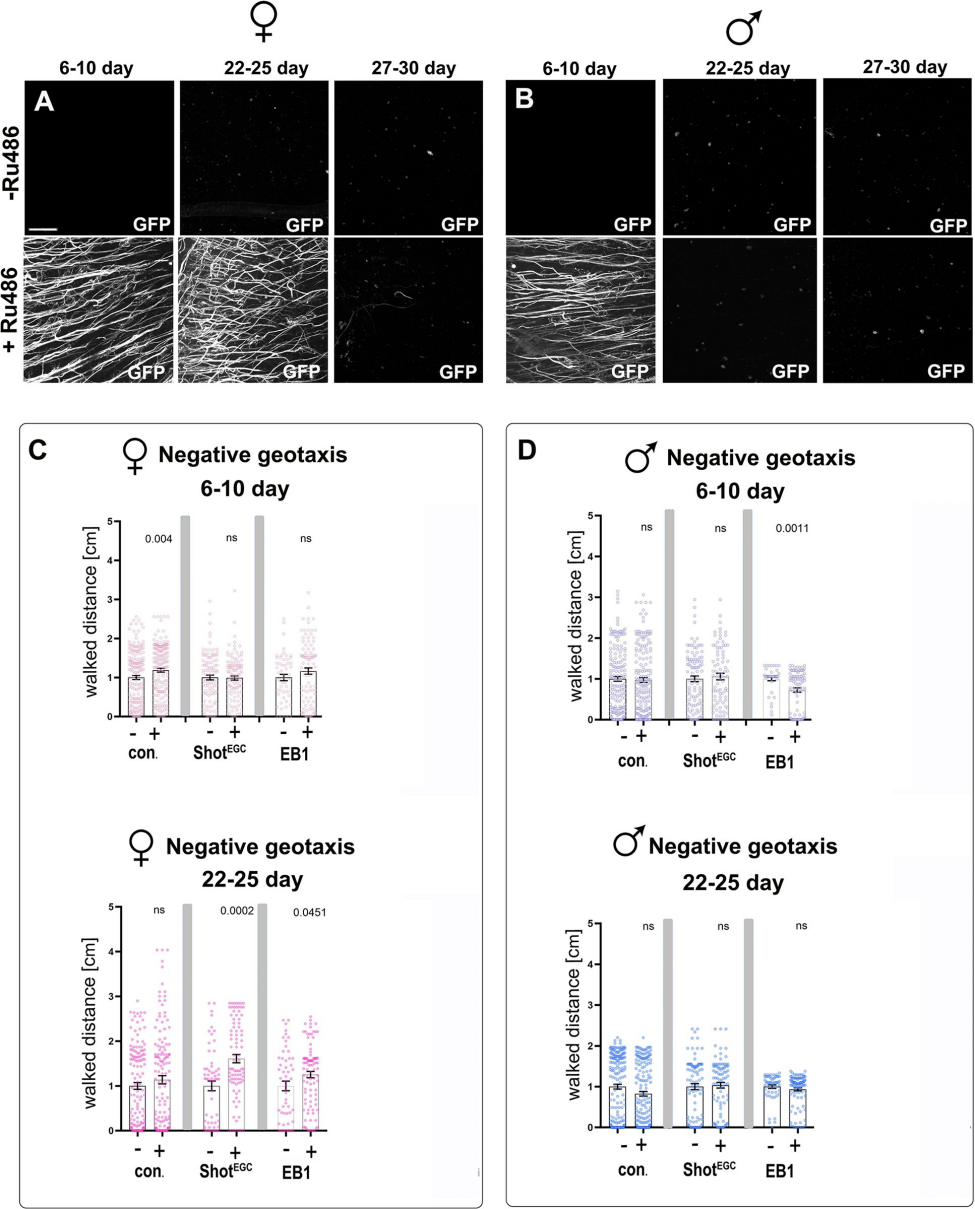
Expression of Shot^EGC^ and EB1 in adult *Drosophila* using the Geneswitch system improve locomotion of aged flies. (**A, B**) Axonal region of medullas from specimen at different ages (6–10, 22–25, and 27–30 days at 29°C), carrying the elav-Gene-Switch system together with UAS-GFP-tagged Shot^EGC^ fragment, which has been continuously maintained in food without (−) or with (+) RU-486. RU-486 induces GFP expression, which is prominent in female and male young specimens. However, GFP expression levels decrease as the specimens age, with males from 22–25 days onwards showing no GFP, and females maintaining GFP expression at 22–25 days which is lost by 27 to 30 days at 29°C. (**C, D**) Negative geotaxis walking assays with flies at 2 different ages (6–10 and 22–25 days old) that have been continuously maintained in food without (−) or with RU-486 (+). Specific conditions are indicated below the X-axes and include control flies or flies expressing either Shot^EGC^ or EB1 in adulthood with the pan-neuronal Elav-GeneSwitch driver. Walking distance of each genotype in the presence of RU-486 has been normalised to the mean walking distance of the same genotype without drug treatment. Data points are shown as circles and as mean ± SEM; *p*-values obtained from comparisons of pairs of same genotypes with or without RU-486 via Mann–Whitney test. For detailed statistical values and genotypes, see Table N within the S1 Tables. All the single values are provided in the S1 Datapoints. Scale bar in A = 20 μm.

To assess whether the improvement of subcellular neuronal phenotypes has an impact on the systemic performance of flies, we examined fly locomotion as a readout known to be affected by ageing [38,71]. For this, we tested the flies negative geotaxis response, which is the natural reaction of flies to walk upwards after being tapped to the bottom of a container. It is usually quantified by measuring the distance flies climb as a function of time (S15A Fig). For our experiments, we used the GeneSwitch system which is a modified GAL4-UAS system where GAL4 expression is induced upon the presence of the drug RU-486 [72]. This system allows us to compare flies from the same backgrounds and genotypes in the absence (without transgene expression) or presence of RU-486 (inducing expression), ruling out the possibility that the different genetic backgrounds of these flies were contributing to the locomotion improvement. Using this approach, adult specimens were reared on standard fly food supplemented with RU-486 throughout their lifespan to induce overexpression of Shot^EGC^::GFP or EB1::GFP. Using Shot-GFP expression as a readout of the GeneSwitch activity, we found that, as expected, feeding flies RU-486-supplemented food induced expression. However, the expression was not maintained for the entire lifespan: in females, expression was observed early on at 6 to 10 days and maintained at 22 days at 29°C, but it was absent in 27-day-old flies or older. In males reared on RU-486-supplemented food, exogenous expression was observed in 6 to 10 days old specimens but was already absent in 22-day-old flies or older (Fig 8A and 8B). As to locomotion capabilities, negative geotaxis tests demonstrated a significant increase in the distance walked by 22–25 days old females reared on RU-486 containing food and expressing Shot^EGC^ or EB1, when compared to same genotype flies but reared without RU-486 (Fig 8C). Such improvement in locomotion was not observed in old male specimens (Fig 8D), in agreement with the GeneSwitch expression profile described above. Young and control (without UAS-transgenes) specimens showed no improvement in locomotion when fed with RU-486 compared to non RU-486 animals.

In addition to the GeneSwitch system, we used the Gal4/Gal80^ts^ system in combination with the pan-neuronal *elav-Gal4* driver to restrict expression of EB1::GFP and Shot^EGC^::GFP to the adult stage (starting after eclosure). We found that young flies at 4 to 5 DAE expressing Shot^EGC^::GFP performed similar to age-matched wild-type controls, whereas EB1::GFP expressing flies showed a slight decrease in locomotion (S15B Fig). Compared to the young flies, older control flies (25 to 26 DAE) showed a stark decrease in locomotion, and this was partially rescued by the expression of Shot^EGC^::GFP (S15B Fig).

Our data strongly suggest that restoring neuronal MT networks improves nervous system function with a positive impact on wider body physiology during ageing. Given that locomotion requires physiology outside the nervous system likewise affected by ageing, such as the activity of muscles, a full rescue was not expected. Therefore, carefully designed strategies targeting MT regulation might provide a path to ameliorate age-related pathological changes in neurons and improve their functionality.

## Discussion

In this study, we established a novel model to study neuronal ageing in the *Drosophila* brain. This model is highly accessible to genetic and experimental manipulations, and it displays well-conserved hallmarks of ageing in only 5 weeks. These age-related morphological changes in *Drosophila* axons and synapses, reminiscent of alterations described in higher organisms, include the appearance of axonal swellings, decrease in axonal calibre and the breakdown of synaptic terminals accompanied by membrane swellings or oedemas [10–17]. Our study further shows that MT deterioration partly due to decreased replenishment of MTs, is an important change that precedes the other signs of ageing pathology, and that it is likely caused by an age-dependent reduction in the function of MT regulators. Importantly, we showed that reducing key MT regulators such as Tau, EB1, or Shot, exacerbates ageing-associated MT defects as well as axonal and synaptic atrophy. As an additional important outcome, our work proved that protecting and enhancing MTs in mature neurons prevents age-related axonal and synaptic deterioration. This MT-based approach also improved neuronal functionality, which naturally declines during ageing.

We propose therefore that MT aberrations can be considered an important causal factor of age-induced neuronal decay, providing new mechanistic insight into neuronal atrophy during normal ageing. Our work also indicates the possibility of employing MT-focussed strategies to protect neurons from deteriorating during ageing.

### Neuronal ageing hallmarks and their conservation with higher organisms

Our study revealed that the number of T1 neurons is not altered during ageing (S2 Fig). Instead, their axons and synaptic terminals experience remarkable morphological alterations over time (Fig 1). Overall, axons became thinner and developed swellings, and synaptic terminals appeared to swell and fractionate. Similar morphological alterations have been described in several brain regions from aged rodents and primates. For example, synaptic terminals in the rat hippocampus and mouse cerebellum develop swellings or oedemas with age [15–17]. Furthermore, axonal swellings, or diverticula, were found in axons within the dorsolateral prefrontal cortex of aged rhesus monkey brains [10], and the diameter of axons within the mouse peripheral nervous system was found to decrease with age [11–13,73]. Critically, such biological changes that develop naturally during ageing in higher organisms appeared to occur in the absence of neuronal loss [74] and correlate with functional decay of neuronal networks [75,76]. Indeed, axonal swellings are considered a hallmark of axonal degeneration and axonal atrophy [77], and axonal calibre/diameter is a key determinant of the conduction properties of neurons, with thinner axons correlating with decreased conductivity [57]. Therefore, the presented herein age-dependent alterations in *Drosophila* neurons are consistent with those observed in mammals, proving the validity and efficacy of our model.

### The importance of the MT cytoskeleton in neuronal ageing

Although none of the previous studies linked the aforementioned morphological alterations to defects in the MT cytoskeleton, MTs have been proposed to be affected by ageing in other invertebrates models [78] and in higher organisms [79]. Reduction in MT density and alterations in MT organisation within axons are both reported to take place within human and nonhuman primate brains during ageing [10,25]. In human pyramidal neurons, a 55% reduction in MT density was reported prior to the formation of Tau neurofibrillary tangles [25], suggesting that changes occur at the level of the cytoskeleton before other bona fide ageing hallmarks. In *Caenorhabditis elegans*, MT bundles are disorganised in aged specimens [78]. Considering the previous reports together with our new findings, we propose that the changes in the MT cytoskeleton observed in aged neurons constitute a widespread phenomenon, apparent in primates and invertebrates.

The work presented here further demonstrates that there is a causative link between MT deterioration during ageing and natural axonal decay exemplified by the decrease in axonal calibre and the formation of axonal swellings. We show not only that MT bundle impairment precedes other neuronal morphological changes, but also that decreasing or enhancing the function of specific MT regulators is sufficient to aggravate or rescue axonal decay, respectively. However, it may be that the appearance of swellings facilitates MT deficits such as unbundling. The mechanisms by which MT deterioration leads to perturbations of the axon are yet not understood. They may involve changes in membrane tension and/or the axonal membrane associated periodic skeleton, both of which are highly dependent on the MT cytoskeleton [53,80,81]. For example, compromising MT integrity generates membrane tension-driven instabilities, leading to axonal beading in cultured neurons [53,82,83]. Whether swellings appear in the same spatial position where MTs may deteriorate is challenging to prove. We expect both swellings and areas of MT deterioration to be dynamic [53], therefore, such correlative study would require long-term live imaging of MTs and axonal membranes in the brain. MT breaks or areas of disorganisation could also halt transport of organelles along the axon, leading to acute accumulations and the development of swellings. In fact, disruptions in axonal transport and aberrant accumulation of organelles along the axon are frequently observed in aged organisms from different species, including rhesus monkey, *Drosophila* and *C*. *elegans* [10,40,84,85]. In *C*. *elegans*, ageing affects the activity of the motor protein UNC-104, preventing its localisation in axons, and rendering neural circuits more vulnerable [84]. We previously found that the loss of Tau and Shot similarly affects the translocation of UNC-104 to axons. Over time, this leads to a decrease of key synaptic proteins in synapses [62]. The fact that MT deterioration enhances synaptic decay and axonal swellings in our model may suggests that transport capacity in T1 neurons also declines with age.

Importantly, we found that by preserving MTs during ageing via increasing the function of specific MT regulators, we could improve aged axon’s health (Figs 7, S9, S13 and S14), thus firmly demonstrating that MTs are essential contributors of neuronal decay during ageing, although we cannot discard that other pathways contribute to brain ageing. Indeed, in neurodegenerative diseases (Alzheimer’s disease, fronto-temporal dementia, Parkinson’s disease, amyotrophic lateral sclerosis, hereditary sensory autonomic neuropathy), significant decay of axonal MT bundles is observed [25,86–91]. While it remains an open question how these diseases cause such alterations, and whether MT decay is occurring in parallel to axonal and synaptic atrophy, our results suggest that MT deterioration could be a mediator. Ageing is considered a key risk factor common to the aforementioned neurodegenerative diseases, and common to both ageing and these pathologies are MT deterioration, axonal swellings, synaptic fragmentation, and defective transport [10,25,30,31,40,85,92–94]. It may be that MT bundle deterioration is a key shared mechanism that, when unrectified, can trigger transport deficits, likely to impact the movements of synaptic material (organelles and proteins) between the soma and the terminal, leading to axonal and synaptic decay. In this model, age would render the maintenance of MT bundles less efficient, ultimately sensitising axons, making them even more vulnerable in disease conditions.

### MT regulation in mature neurons

How axonal MT bundles are maintained in mature neurons and during ageing has gained limited attention in the past. Our tubulin::GFP pulse-chase experiments suggest that there is continued renewal of MTs in mature axons which may involve MT disassembly, nucleation, polymerisation, and repair [24,95]. Yet, MT replenishment appears severely diminished with age. The observed MT breaks in axons and a decrease in the diameter of MT bundles with age might therefore be the direct consequence of net loss of MT volume, due to an increase in MT instability causing MT loss, or a decrease in the rate at which MTs are replaced.

A hint pointing to the mechanisms mediating loss of MT volume during ageing is suggested by our findings that attenuating the function of Tau, EB1, and the spectraplakin Shot, exacerbates ageing-associated MT breaks in axons from adult *Drosophila*. These findings suggest that the regulation of MT homeostasis in axons from adults involve functions of Tau, EB1, and Shot. Tau and Shot ability to promote MT stability [62] as well as EB1 roles in promoting MT polymerisation dynamics and the repair of damaged MT shafts [24,41,96–98] could directly impact the rate at which MTs are lost and replaced in axons of mature neurons during ageing.

Along with the loss of MT volume in aged specimens, we also observed foci of disorganised MTs in mature axons, which are increased in number by reducing the function of Tau, EB1, and Shot. Such foci could arise from a decrease in MT bundling caused by loss of Tau during ageing, since Tau can bundle MTs by the means of its projection domain [99]. Similarly, Shot and other spectraplakins are proposed to bundle MTs through mechanisms not yet understood [41,45]. Alternatively, the loss of linkage of polymerising MTs to cortical actin cytoskeleton can cause aberrant MTs extension into disorganised and curved conformations contributing to disorganised MT foci in aged axons. Shot, EB1, and Tau cooperate in this linkage function in developing neurons [29,41,43,45,100]. This function appears to remain essential during ageing as suggested by our data showing an increase in disorganised MT foci during ageing upon Tau, Eb1, and Shot loss, and the rescue of this phenotype after expression of EB1 and Shot^EGC^.

Our studies suggest that MT deterioration is likely due to a natural age-dependent reduction in the function of MT regulators such as Tau and EB1. It remains to be established what causes such reduction, but altering their posttranslational modification state is a likely explanation [101,102], or they may be impacted by changes in cytoplasmic ROS or Calcium [103,104], all known to change in the ageing brain. Our findings may once more have important parallels to the aged human brain where the function and localisation of Tau are also altered [68,105].

### The value of the MT cytoskeleton as therapeutic target

In this work, we showed that improving MT’s health halts neuronal decay during ageing. By targeting and protecting MTs, we may be able to decrease susceptibility to age-related diseases, for example, by decreasing the age-related decline in axonal transport capacity. The use of such MT-targeting strategies to battle ageing may also be of benefit in other contexts, as comparable decline in MTs might be occurring in other cell types (and/or tissues). For example, altered MT dynamics during spindle formation is a primary cause for age-related chromosome segregation errors in oocytes. Additionally, changes in mitochondria activity observed in aged mouse oocytes are linked to alterations in the cytoskeleton, and together, could contribute to increased infertility in oocytes from female mammals with age [106,107]. These highlight the potential of targeting MTs to slow down the ageing process in different cell types.

Several studies, including ours, have pointed to the validity of this approach. For example, targeting MTs by increasing their acetylation, which is frequently linked to MT stability, protects axons and transport of mitochondria in a mouse trauma model caused by intracerebral haemorrhage [108]. Furthermore, manipulation of MTs by the use of MT-targeting agents (MTAs) has proven valuable in the context of age-related neurodegenerative diseases, as suggested by recent reports highlighting the beneficial therapeutic effects of MT-stabilising compounds such as Epothilone D in several models of tauopathies [109–111]. However, MTAs can induce toxicity and trigger side effects such as neuropathy, and their prolonged usage can lead to drug resistance [111]. Therefore, modifying MTs by manipulating MT-binding proteins may be a more promising alternative approach [102,112].

## Material and methods

### Fly stocks and husbandry

The following fly stocks were used in this project. Gal4 driver lines include: *elav-Gal4* (3^rd^ chromosomal, expressing pan-neuronally at all stages; [113]), *GMR31F10-Gal4* (Bloomington stock 49685; expressing in T1 medulla neurons; [48]), *GMR53G02-GAL4* (Bloomington stock 50446; expressing in T2 medulla neurons; [114]), *elav-GS-Gal4* (Bloomington stock 43642). Mutant stocks: EB1 deficiency *Df(2R)Exel6065* (EB1^*Df*^; Bloomington stock 7547), *chico*^*1*^ (Bloomington stock 10738). Lines for transgene targeted expression were *UAS-Shot*^*EGC*^*-GFP* and *UAS-EB1-GFP* [41], *UAS-GFP-α-tubulin84B* [115], *UAS-RedStinger* (Bloomington stock 84277), and *UAS-myr*::*tdTomato* (Bloomington stock 32222). Lines for targeted gene knockdowns were *UAS-tau*^*RNAi*^ (*tau*^*GD25023*^ Vienna *Drosophila* RNAi Center, Austria [116]), *UAS-shot*^*RNAi*^ [117] and *UAS-EB1*^*RNAi*^ (*EB1*^*24451*^ Vienna *Drosophila* RNAi Center, Austria, [41]).

For ageing experiments, all flies were maintained during ageing at low density (maximum of 20 flies per vial) on standard sugar-yeast molasses medium, at 29°C unless otherwise specified. Experimental flies were transferred to fresh vials every 3 days. Equal number of males and females were used in this study.

For RU-486 drug feeding for GeneSwitch experiments, flies eclosed on food supplemented with the indicated concentration of RU-486 (Merck-group M8046). For RU-486 containing food, a 25 mg/ml stock solution in 100% ethanol was used to make 25 μg/ml solution of RU-486 in water, and 300 μl was placed onto the surface of each fly food vial. Control vials were generated by using 300 μl of a solution at equal final ethanol concentration. Vials were left to dry for 24 h. Experimental flies were transferred to fresh vials with RU-486 or control solution every 3 days.

### Dissection of adult fly heads

Dissections of *Drosophila* brain were performed in Dulbecco’s PBS (Sigma, RNBF2227) after briefly anaesthetising the specimens on ice for 1 min and 30 s. This short ice treatment showed no decrease of synaptic MTs when compared to brains obtained from flies anaesthetised with CO_2_ (S16 Fig). For live imaging, dissected brains with their laminas and eyes attached were placed into a drop of Dulbecco’s PBS on MatTek glass bottom dishes (P35G1.5-14C), with a spacer and covered by coverslips. Brains were immediately imaged.

To label MTs with the live cell MT probe Sirt-Tubulin, freshly dissected brains were incubated in the dark for 1 h in Dulbecco’s media containing Sirt-Tubulin (at 1:100 dilution). Brains were washed in Dulbecco’s media and mounted for live imaging as indicated above.

### Immunohistochemistry

*Drosophila* brain dissections were performed as explained above and fixed in 4% paraformaldehyde (PFA; in 0.05 M phosphate buffer, pH 7 to 7.2) for 30 min at room temperature (RT). For anti-EB1 staining, brains were fixed at −20°C for 10 min in +TIP fix (90% methanol, 3% formaldehyde, 5 mM sodium carbonate, pH 9; stored at −80°C [43]). Brains were washed in PBT (PBS with 0.3% Triton X-100). Antibody staining and washes were performed with PBT.

Antibodies used include: anti-DmEB1 (gift from H. Ohkura; rabbit, 1:2,000; [96]); anti-GFP (ab290, Abcam, 1:500); FITC-, Cy3- or Cy5-conjugated secondary antibodies (Jackson ImmunoResearch); and STAR 580 (Abberior STAR 580) for super resolution. Specimens were embedded in Vectashield (VectorLabs).

### Microscopy and data analysis

For all ageing parameters, *Drosophila* brains were imaged at the Centre for Cell Imaging at the University of Liverpool with a 3i Marianas Spinning Disk Confocal Microscope, except for the study of MT diameters where the STED system STEDYCON or the LSM 900 Airyscan2 was used.

To measure ageing hallmarks in the optic lobe of adult flies (except for MT diameter), brains were dissected as explained above and immediately imaged with a 3i Marianas Spinning Disk Confocal Microscope. A section of the medulla columns comprising the 4 most proximal axonal terminals was used to quantify MTs axonal phenotypes and the number of swellings. To measure Tub-GFP incorporation into axonal MTs, a line was drawn along these axons, using the Segmented Line Tool in Fiji, in order to measure the mean intensity. To measure the intensity from UAS-myr::tdTomato, a fitting box around the axon was drawn using the Polygon Selection Tool and the mean intensity calculated. To measure axonal and MT core diameter, brain images were orientated so the columns of medulla axonal projections were displayed horizontally. The Line Tool of ImageJ was used to measure the diameter of axons and their MT core, at evenly spaced unbiased positions predetermined by a grid. A mean per axon from multiple measured points was obtained. To score synaptic readouts, a square was drawn through the central part of the medulla and synapses within scored (approximately 30 to 50 per medulla). Synaptic MTs and morphological categorisation were manually calculated. To measure the area of synaptic terminals, the Freehand selection Tool was used to draw around the membrane, marked by myr::tdTomato. Nuclei labelled with RedStinger were quantified manually in Fiji, Image J software.

For pixel intensity analysis of Tau in axons, maximal projections were generated from Z-stacks using Fiji, Image J software. With the Line Tool, 4 regions of interest (ROI) per medulla, spanning across 8 axon bundles, were positioned parallel to each other. Intensity histograms were generated per ROI and pixel intensity threshold bins for grey values >220 were set. Peaks above the threshold were counted and the mean was taken across the 4 ROIs. For pixel intensity analysis of EB1 in axons, maximal projections from 6 stacks were generated from 2 areas of each medulla (upper and lower half). Ten medulla axon columns were selected using the “manual draw” tool on ImageJ, and mean intensity was measured in the EB1 channel. The mean intensity per medulla was calculated and plotted.

### MT-binding assay and western blots

MT-binding assays were performed as previously published [69,118]. In brief, for each age group, 6 heads were homogenised in 40 μl of MT-binding assay buffer (100 mM MES (pH 6.8), 500 μm MgSO4, 1 mM EGTA, 4 mM DTT, 2 mM dGTP, 20 μm Taxol, 0.1% triton X-100, 30 mM NaF, 20 mM sodium pyrophosphate, 40 mM 2-glycerophosphate, 3.5 mM sodium orthovanadate, 10 μm staurosporine, and protease inhibitor cocktail). Homogenates were centrifuged at 12,000 g for 1 h at 4°C. The supernatant, representing the cytosolic fraction, was transferred to a fresh tube while the pellet, representing the fraction enriched for MT-bound proteins, was resuspended in 20 μl of the MT-binding assay buffer. Samples were heated in the Laemmli buffer for 10 min at 95°C and separated by electrophoresis.

### Western blots

For total protein level analysis, heads were homogenised in an ice-cold RIPA buffer, supplemented with Protease and Phosphatase inhibitors. The samples were heated in the Laemmli buffer for 10 min at 95°C before electrophoresis. For electrophoresis separation, extracts were resolved in 10% Stain-free SDS-PAGE gels (BioRad) in running buffer (25 mM Tris Base, 192 mM Glycine, 10% w/v SDS) and transferred to a nitrocellulose membrane followed by blocking in 5% bovine serum albumin for 1 h at RT. Proteins were probed with primary antibodies and detected by incubation with HRP-conjugated secondary antibodies (Invitrogen). The following primary antibodies were used: anti-GAPDH (Thermo Fisher GA1R), anti-GFP (Abcam 6673), anti-actin (JLA20, MercK), anti-a-tubulin (T9026, Merck), anti-DTau (L. Partridge), and anti-EB1 (H. Ohkura). The levels of GAPDH and actin used as loading controls were found not to difference between head tissue from young and old specimens (S17 Fig).

### Negative geotaxis assay

One day prior to the experiment, a maximum of 20 flies were transferred to fresh media vials. On the day of the experiment, flies were transferred to graduated vials without the use of CO_2_. Flies were given 30 min to acclimatise to the new environment. Next, flies were tapped to the bottom of vials and their walking behaviour was filmed. For the Gal4/Gal80^ts^ experiment, the position of each fly was annotated after 15 s. For the GeneSwitch experiment, we first determined the time point at which control untreated flies reached 2/3rds of the maximum walked distance. The established time point was used to determine the position reached by each fly during this time. Three technical replications were conducted, which included all together around 220 to 340 young flies and between 170 and 290 old flies per genotype for Gal4/Gal80^ts^ and between 330 and 700 per genotype and age for the GeneSwitch experiments.

### Statistical analysis

Statistical analyses were performed in GraphPad Prism 9 using Mann–Whitney rank sum tests (indicated as P_MW_), via Kruskall–Wallis ANOVA multiple comparison tests or Chi^2^ (P_Chi_), with 95% confidence intervals. The exact *p*-values are indicated in the graphs and the exact sample size and other statistical values are indicated in Tables A to Q in the S1 Tables.

## Supporting information

S1 Fig Widespread deterioration of neurons within the *Drosophila* visual system with age. (**A and B**) Axons and terminals of the L2 neurons within the medulla of the optic lobe from young (5–7 days, A) and old specimens (28–30 days, B), labelled with the plasma membrane marker myr-Tom (myr). In aged specimens, axons show thinning (arrow heads) and swellings (asterisks; dashed blue boxes shown as 1.7-fold magnified images below). Scale Bar for A and B can be found in A bottom right = 10 μm.
(TIF)

S2 Fig Absence of neuronal death in T1 neurons during ageing. (**A**) T1 neurons labelled with the nucleus marker RedStringer (magenta) and GFP-tagged α-tubulin (green). Dashed blue boxes shown as 2-fold magnified image in the inset below. (**B**) Quantification of nuclei labelled with RedStinger at different ages to determine the number of T1 neurons. Data are shown as mean ± SEM of nuclei per medulla with individual data points in blue; *p*-values obtained with Kruskall–Wallis ANOVA test for the different conditions are indicated in the graph. For detailed statistical values and genotypes, see Table B within the S1 Tables. All the single values are provided in the S1 Datapoints. Scale Bar in A = 30 μm.
(TIF)

S3 Fig The deterioration of T1 neurons during ageing is independent of temperature and marker expression. **(A–D**) T1 axons (top) and synaptic terminals (bottom) in the medulla of flies labelled with the plasma membrane marker myr-Tom (myr) using the UAS/Gal4/Gal80^ts^ system. Gene expression is induced by the shift of temperature from 18°C to 29°C. Flies were kept at 18°C throughout development and adult life until the last 4 days before imaging, at which point they were shifted to 29°C to induce myr-Tomato gene expression. Young specimens (A and C; 4–7 days old flies with 0–3 days at 18°C “Off” + 4 days at 29°C “On”) are compared to old specimens (B and D; 57–60 days old flies with 53–56 days at 18°C “Off” + 4 at 29°C “On”). In aged specimens, axons show thinning (arrowheads) and swellings (asterisks), whereas synaptic terminals appear swollen and broken down (arrows and dashed blue box shown as 3-fold magnified image in the inset below). (**E**) Quantifications of phenotypes shown in A–D, with young versus old indicated on the X-axes. In the top 2 graphs, data points are shown in blue and as mean bars ± SEM (*p*-values obtained via Mann–Whitney test are indicated above). For terminal morphology, data are shown as distribution of normal versus swollen/broken synapses (significance obtained via Chi-square test indicated above). Data were taken from a minimum of 5 specimens per age group. For detailed statistical values and genotypes, see Table C within the S1 Tables. All the single values are provided in the S1 Datapoints. Scale bar in A represents 20 μm in all images.
(TIF)

S4 Fig Extra examples of MT changes during ageing. (**A**) MT bundles labelled with GFP-tagged α-tubulin (in inverted greyscale) from axons of T1 neuron, from specimens of different age groups: young (6–10 days old) or old (29–31 or 38–42 days old). Aged flies show MT unbundling (arrowheads) as well as breaks and thinning of MTs (white arrows) compared to normal/intact stretches of MTs, blue arrows. (**B**) MT bundles from axons of T1 neuron, from specimens labelled with GFP-tagged α-tubulin (tub, magenta) and the plasma membrane marker myr-Tom (myr, green) show MT unbundling (arrowheads) in 19–23 days specimens. Examples of typical axonal swelling which were quantified in this study (blue arrows) can be observed in 29–31 days old specimens. At age 5–10 days, 20% of swellings contain unbundled disorganised microtubules; at age 19–23, 21%, at age 29–31, 40% and at age 38–42, 56% of the swellings contain unbundle disorganised microtubules. Scale bar = 4 μm.
(TIF)

S5 Fig The MT bundle within axons thinners with age. (**A and B**) MT axonal bundles from T1 neurons from young (8–10 days old) and old (34–36 days old) specimens labelled with GFP-tagged α-tubulin were imaged using Airy scanning high-end confocal microscopy (A) or STED super-resolution microscopy (B). (**C and D**) Images obtained using both imaging techniques have been used to quantify the diameter of MT bundles (C with Airy scanning and D with STED). Results are presented as mean ± SEM with individual data points in blue. *P*-values obtained with Mann–Whitney test are indicated in each graph. Data points were taken from at least 13 specimens per age group for Airy Scanning and 3 specimens for STED. For detailed statistical values and genotypes, see Table D within the S1 Tables. All the single values are provided in the S1 Datapoints. Scale bar in A represents 4 μm and in B 0.5 μm.
(TIF)

S6 Fig MT alterations during ageing are independent of the MT reporter expression and are specific to aged specimens. (**A–D**) T1 axons (A and B) and synaptic terminals (bottom and D) in the medulla of flies with their MTs labelled with GFP-tagged α-tubulin (tubulin, in greyscale images and green in insets) and the plasma membrane marker myr-Tom (myr, magenta), using the UAS/Gal4/Gal80^ts^ system. Gene expression is induced by the shift of temperature from 18°C to 29°C. Flies were kept at 18°C throughout development and adult life until the last 15 days before imaging, at which point they were shifted to 29°C to induce gene expression (15 days expression was used to achieve sufficient MT labelling). Young specimens (A and C; 15–17 day old flies with 0–2 days at 18°C “Off” + 15 days at 29°C “On”) are compared to old specimens (B and D; 69–71 days old flies with 54–56 days at 18°C “Off”+ 15 at 29°C “On”). In A and B, cyan encircled boxes are shown 3.7-fold magnified below with old flies in B, showing MT unbundling (insets 1 and 2 in B) as well as breaks and thinning of MTs (insets 3 and 4 in B) compared to young axons. (D) Old flies also show a reduction of splayed MTs in synaptic terminals when compared to young controls in C (boxed areas shown as 2-fold magnified insets). (**E**) Quantifications of phenotypes shown in A–D, with young versus old indicated on the X-axes; data points are shown as blue circles and as mean ± SEM; *p*-values obtained via Mann–Whitney tests are indicated above. Data were taken from a minimum of 5 specimens per age group. For detailed statistical values and genotypes, see Table E within the S1 Tables. All the single values are provided in the S1 Datapoints. Scale bar = 10 μm.
(TIF)

S7 Fig Tau-GFP and EB1 localisation at MTs. (**A**) Axonal region of the medulla from a 5–7 days old specimen labelled with endogenous GFP-tagged Tau (green in coloured image and in inverted greyscale image) and the live cell MT probe Sirt-Tubulin (magenta in coloured image and in inverted greyscale image), showing colocalisation of Tau to MTs (arrowheads in A). (**B**) Axonal region of the medulla from a 3- to 5-day-old specimen labelled with anti-EB1 (green in coloured image and in the inverted greyscale image) and expressing GFP-tagged α-tubulin in T1 neurons (magenta in coloured image and in the inverted greyscale image) showing EB1 signal colocalising to T1 MTs (arrowheads in B). Scale bar in A = 10 μm.
(TIF)

S8 Fig Examples of age-related MT decay and ageing hallmarks caused by loss of *EB1*, *Tau*, and *Shot*. (**A–D**) Representative images of T1 axons in the medulla of 28–30 days old flies labelled with GFP-tagged α-tubulin (tubulin, greyscale and magenta in insets) and the plasma membrane marker myr-Tom (myr, green in insets). Aged neurons in the absence (A) or presence of Tau knockdown (*Tau*_*RNAi*_ in B), *Shot* knockdown (*Shot*_*RNAi*_ C), and EB1 knockdown in a deficiency heterozygous background (*EB1*_*RNAi*_*; EB1*^*+/*Df^ in D). Knockdown of the above MT regulators enhance phenotypes in ageing neurons, comprising axon swellings often displaying MT unbundling (arrowheads) and MT breaks (asterisks); boxed areas shown as 2-fold magnified double/single-channel images below. Boxed area shown as 2-fold magnified double/single-channel images below. Scale bar in A = 10 μm.
(TIF)

S9 Fig The deterioration of synaptic terminals during ageing can be exacerbated or rescued by altering the function of MT regulators. (**A–D**) Terminals of the T1 medulla projections from old specimens (28–30 days A and C; 29–31 days B and D) with the plasma membrane labelled with myr-Tom (myr) and their MTs with GFP-tagged α-tubulin (A–C) or GFP-tagged Shot^EGC^ (D). Ageing phenotypes at the synaptic terminals, including a decrease in synaptic MTs (arrowheads) and an increase in broken and swollen terminals (asterisks), are enhanced in the presence of combined Tau and Shot knockdowns (Tau_RNAi_ Shot_RNAi_ in C). The same ageing phenotypes are suppressed by the expression of Shot^EGC^ (D). (**E**) Quantifications of phenotypes shown in A to D plus conditions of EB1 ectopic expression. Specific conditions are indicated below the X-axes. In the upper graphs, data points are shown as blue circles and as mean ± SEM; *p*-values obtained via Mann–Whitney test are indicated above. For terminal morphology, data are represented as distribution of normal versus swollen/broken synapses; significance obtained via Chi-square test is indicated above. Data were taken from a minimum of 14 specimens per group. For detailed statistical values and genotypes, see Table J within the S1 Tables. All the single values are provided in the S1 Datapoints. Scale bar in A represents 10 μm in A–D.
(TIF)

S10 Fig MT decay and ageing hallmarks are not present in young specimens with *EB1*, *Tau*, and *Shot* knockdowns. (**A and B**) Representative images of T1 axons in the medulla of 3–7 days old flies labelled with GFP-tagged α-tubulin (tubulin, greyscale and magenta in insets) and the plasma membrane marker myr-Tom (myr, green in insets). Images show specimens in the absence (A) or presence of combined Tau and Shot knockdowns (*Tau*_*RNAi*_ *Shot*_*RNAi*_ in B). MTs axonal bundles maintain their diameter and organisation, and axons lack swellings and areas of decreased diameter in the presence of knockdowns; boxed area shown as 2-fold magnified double/single-channel images below. (**C**) Quantifications of phenotypes shown in A and B plus conditions of further knockdowns for Tau (*Tau*_*RNAi*_), Shot (*Shot*_*RNAi*_), and EB1 in an EB1 heterozygous background (*EB1*_*RNAi*_*; EB1*^*+/*Df^); specific knockdowns are indicated below the X-axes. Data points are shown as blue circles and as mean ± SEM; *p*-values obtained via Mann–Whitney tests are indicated above. Data were taken from a minimum of 13 specimens per age group and condition with the exception of 8 specimens for Shot. For detailed statistical values and genotypes, see Table K within the S1 Tables. All the single values are provided in the S1 Datapoints. Boxed area shown as 2-fold magnified double/single-channel images below. Scale bar in A = 10 μm.
(TIF)

S11 Fig Examples of young specimens with EB1, Tau, and Shot single knockdowns. (**A, D**) Representative images of T1 axons in the medulla of 5–8 days old flies labelled with GFP-tagged α-tubulin (tubulin, greyscale and magenta in insets) and the plasma membrane marker myr-Tom (myr, green in insets). Images show young specimens in the absence (A) or presence of Tau knockdown (*Tau*_*RNAi*_ in B), Shot knockdown (*Shot*_*RNAi*_ in C), and EB1 knockdown in a deficiency heterozygous background (*EB1*_*RNAi*_*; EB1*^*+/*Df^ in D). In the presence of knockdowns, the diameter and organisation of MTs axonal bundles are unaltered, and axons lack swellings and their diameter are maintained; boxed area shown as 2-fold magnified double/single-channel images below. Scale bar in A represents 10 μm in all images.
(TIF)

S12 Fig Shot ^EGC^ localisation at MTs. (**A**) Axonal region of the medulla from a 5–7 days old specimen expressing the GFP-tagged Shot^EGC^ fragment and the plasma membrane marker myr-Tom (myr) in T1 neurons and labelled with the live cell MT probe Sirt-Tubulin (Myr in red, Shot^EGC^ in green and Tubulin^Sirt^ in blue, single channels are shown in inverted greyscale images). Shot^EGC^ colocalises to the shaft of MTs (some examples are indicated with arrowheads). Scale bar in A = 10 μm.
(TIF)

S13 Fig Axonal ageing phenotypes are ameliorated by expression of EB1. (**A–F**) T1 axons in the medulla are labelled with the plasma membrane marker myr-Tom (myr, greyscale in A and B, age of specimens between 35 and 38 days old) or with GFP-tagged α-tubulin (tubulin, age of specimens between 26 and 28 days old). Ageing phenotypes including axon swellings (arrows in A), axon thinning (inset in A), MT unbundling (inset in C), and sparse synaptic MTs (inset in E) can be observed in old specimens, but are absent upon T1-specific expression of EB1 (B, D, F). Scale bar in A–D represents 10 μm, and 14 μm in E and F.
(TIF)

S14 Fig Post-developmental expression of Shot^EGC^ but not of EB1 is sufficient to rescue all axonal ageing phenotypes. (**A–C**) T1 axons in the medulla of old specimens of 27–33 days old control brains (A), overexpressing EB1 (B), or Shot^EGC^ (C). Brains are labelled with the plasma membrane marker myr-Tom (myr, greyscale images in A–C, and green in double-channel images) and with the MT markers GFP-tagged α-tubulin, in A and B, or GFP-tagged Shot^EGC^ in C (MTs for both and in magenta in double-channel images). Using the UAS/Gal4/Gal80^ts^ system, gene expression is induced after development by shifting newly eclosed flies from 18°C to 29°C. Ageing phenotypes including axon swellings, axon thinning (A), MT thinning (boxed area 1 in A), and MT unbundling (boxed area 2 in A) can be observed in old specimens and specimens overexpressing EB1 post-development, but are absent upon post-developmental expression of Shot^EGC^ in adult T1 neurons (A and B compared to C, boxed areas 1 and 2 shown as 2-fold magnified inset were MTs are labelled with GFP-tagged α-tubulin in A and B, or GFP-tagged Shot^EGC^ in B). (**C**) Quantifications of phenotypes in old specimens shown in A and B, plus conditions of post-development expression of EB1 in old specimens in C (27–33 days old). Specific conditions are indicated below the X-axes; data points are shown as blue circles and as mean ± SEM; *p*-values obtained via Mann–Whitney test are indicated above. Data were taken from a minimum of 23 specimens per group. For detailed statistical values and genotypes, see Table M within the S1 Tables. All the single values are provided in the S1 Datapoints. Boxed area shown as 2-fold magnified double/single-channel images below. Scale bar in A represents 10 μm.
(TIF)

S15 Fig The decline in locomotion of aged flies improves upon adult expression of Shot^EGC^. (**A**) Representation of the negative geotaxis walking assays with flies allowed to walk upwards for 15 s, in a narrow-graduated cylinder. (**B**) Quantifications of the distance walked by flies in 15 s at 2 different ages 4–5 days old and 25–26 days old. Specific conditions are indicated below the X-axes, and include control flies or flies expressing either Shot^EGC^ or EB1 in adulthood with the pan-neuronal *elav-Gal4* driver using the UAS/Gal4/Gal80^ts^ system (gene expression is induced after development is completed by shifting newly hatched flies from 18 to 29°C, controls are treated with the same regime but are lacking the transgene). Data points are shown as blue circles and as mean ± SEM. *P*-values obtained via Kruskall–Wallis ANOVA multiple comparison tests are indicated in the graphs. Data were taken from a minimum of 140 specimens per group. For detailed statistical values and genotypes, see Table O within the S1 Tables. All the single values are provided in the S1 Datapoints.
(TIF)

S16 Fig Comparison of the effect on T1 MTs of Ice/cold and CO_2_-based anaesthesia treatments prior dissection. **(A and B**) Synaptic terminals with labelled MTs with GFP-tagged α-tubulin (tubulin), from 4–7 days old specimens which have been anaesthetised for 90 s, either by incubation on ice (A) or exposure to CO_2_ (B) (boxed areas shown as 2-fold magnified insets). (**C**) Quantifications of synaptic MTs from similar images shown in A and B, with type of anaesthesia treatment indicated below the X-axes; data points are shown as blue circles and as mean ± SEM; *p*-values obtained via Mann–Whitney test. For detailed statistical values and genotypes, see Table P within the S1 Tables. All the single values are provided in the S1 Datapoints. A scale bar can be found in A, bottom left (A and B, 10 μm).
(TIF)

S17 Fig The levels of loading control proteins Gapdh and Actin do not change during ageing. Quantifications of total Gapdh and Actin from western blots and relative to the total protein are compared between young (4–9 days at 29°C) and old (29–32 days at 29°C) specimens, demonstrating there is no significant difference between young and old. The graphs depict data points that represent independent lysates, and mean bars ± SEM; *p*-values obtained via Mann–Whitney test. Values have been normalised to the correspondent young for each experiment. For detailed statistical values and genotypes, see Table Q within the S1 Tables. All the single values are provided in the S1 Datapoints, and all western blots can be found in the S1 Western Blots.
(TIF)

S1 Tables Collection of tables including genotypes and correspondent statistical details. The statistical details and specific genotypes for all qualifications in this study can be found in this collection of tables.
(PDF)

S1 Datapoints Underlying datapoints used for statistical analysis. All the datapoints for all quantitative analysis are found in this excel. The values from the different figures can be found in different sheets at the bottom of the excel file.
(XLSX)

S1 Western Blots Underlying western blots used for statistical analysis in Fig 5 and S16. Compilation of western blots used for quantification in Figs 5 and S16.
(PDF)

## Abbreviations

DAE: days after eclosure
MT: microtubule
MTA: microtubule-targeting agent
MTBP: microtubule-binding protein
ROI: region of interest
ROS: reactive oxygen species
RT: room temperature
STED: stimulated emission depletion
