# Supplementary figures and images for: Neuronal ageing is promoted by the decay of the microtubule cytoskeleton
Source: PLoS Biol. 2024 Mar 13;22(3):e3002504. doi: 10.1371/journal.pbio.3002504 (PMC10962844; doi:10.1371/journal.pbio.3002504)

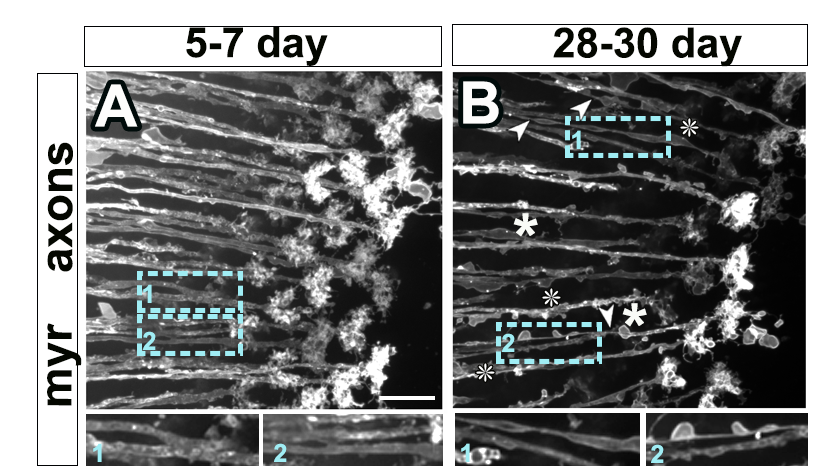

Supplement: S1 Fig — (A and B) Axons and terminals of the L2 neurons within the medulla of the optic lobe from young (5–7 days, A) and old specimens (28–30 days, B), labelled with the plasma membrane marker myr-Tom (myr). In aged specimens, axons show thinning (arrow heads) and swellings (asterisks; dashed blue boxes shown as 1.7-fold magnified images below). Scale Bar for A and B can be found in A bottom right = 10 μm. (TIF) [file pbio.3002504.s001.tif]

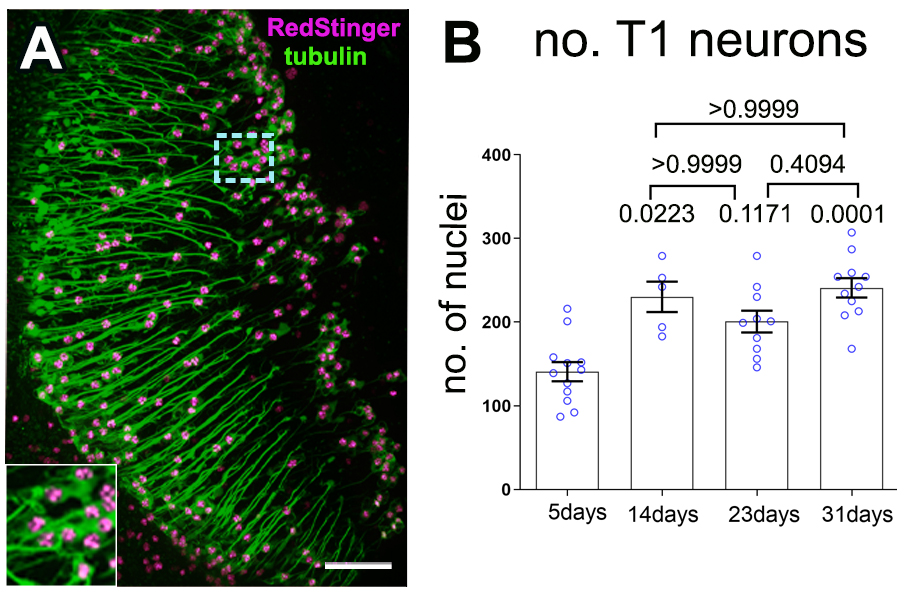

Supplement: S2 Fig — (A) T1 neurons labelled with the nucleus marker RedStringer (magenta) and GFP-tagged α-tubulin (green). Dashed blue boxes shown as 2-fold magnified image in the inset below. (B) Quantification of nuclei labelled with RedStinger at different ages to determine the number of T1 neurons. Data are shown as mean ± SEM of nuclei per medulla with individual data points in blue; p-values obtained with Kruskall–Wallis ANOVA test for the different conditions are indicated in the graph. For detailed statistical values and genotypes, see Table B within the S1 Tables. All the single values are provided in the S1 Datapoints. Scale Bar in A = 30 μm. (TIF) [file pbio.3002504.s002.tif]

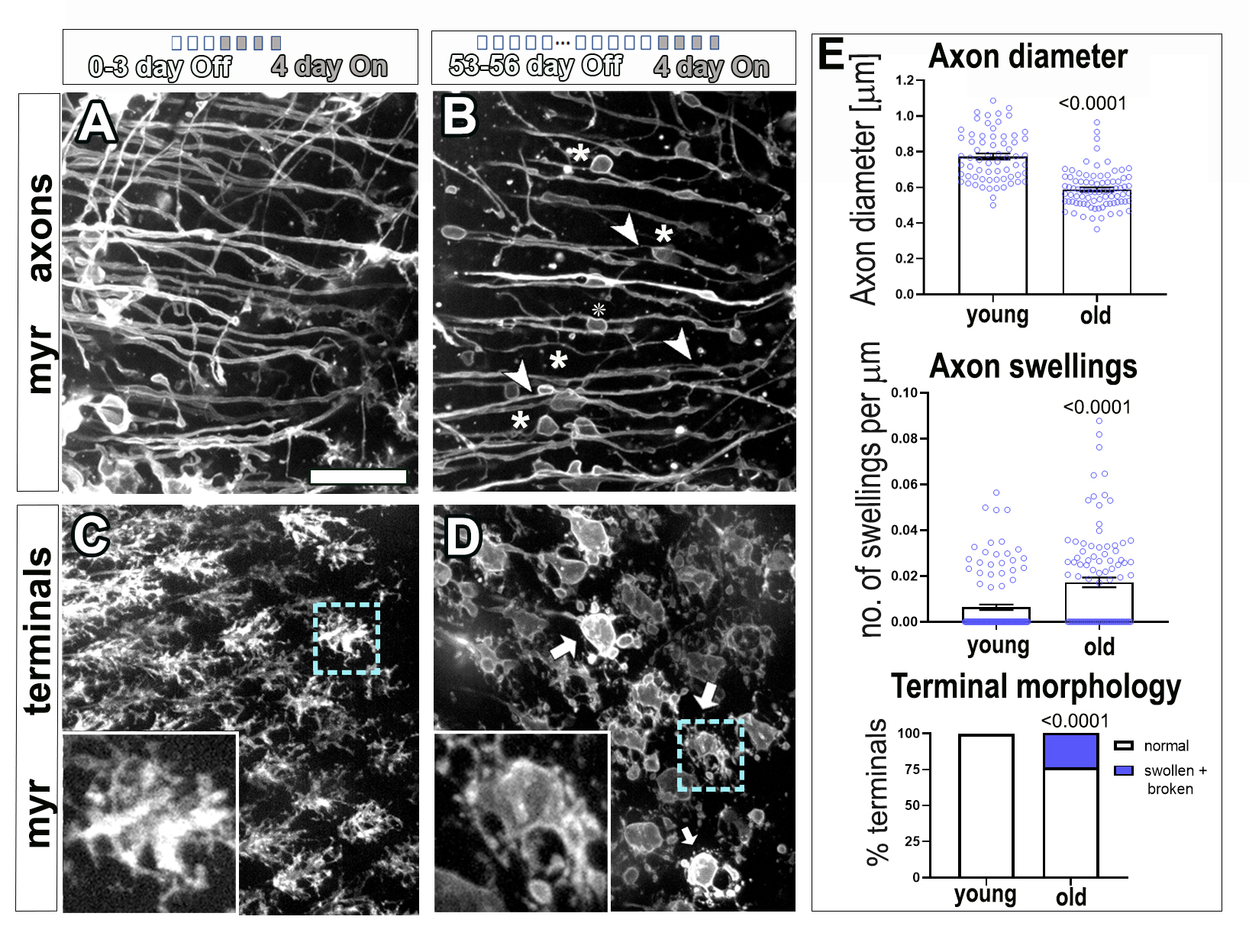

Supplement: S3 Fig — (A–D) T1 axons (top) and synaptic terminals (bottom) in the medulla of flies labelled with the plasma membrane marker myr-Tom (myr) using the UAS/Gal4/Gal80ts system. Gene expression is induced by the shift of temperature from 18°C to 29°C. Flies were kept at 18°C throughout development and adult life until the last 4 days before imaging, at which point they were shifted to 29°C to induce myr-Tomato gene expression. Young specimens (A and C; 4–7 days old flies with 0–3 days at 18°C “Off” + 4 days at 29°C “On”) are compared to old specimens (B and D; 57–60 days old flies with 53–56 days at 18°C “Off” + 4 at 29°C “On”). In aged specimens, axons show thinning (arrowheads) and swellings (asterisks), whereas synaptic terminals appear swollen and broken down (arrows and dashed blue box shown as 3-fold magnified image in the inset below). (E) Quantifications of phenotypes shown in A–D, with young versus old indicated on the X-axes. In the top 2 graphs, data points are shown in blue and as mean bars ± SEM (p-values obtained via Mann–Whitney test are indicated above). For terminal morphology, data are shown as distribution of normal versus swollen/broken synapses (significance obtained via Chi-square test indicated above). Data were taken from a minimum of 5 specimens per age group. For detailed statistical values and genotypes, see Table C within the S1 Tables. All the single values are provided in the S1 Datapoints. Scale bar in A represents 20 μm in all images. (TIF) [file pbio.3002504.s003.tif]

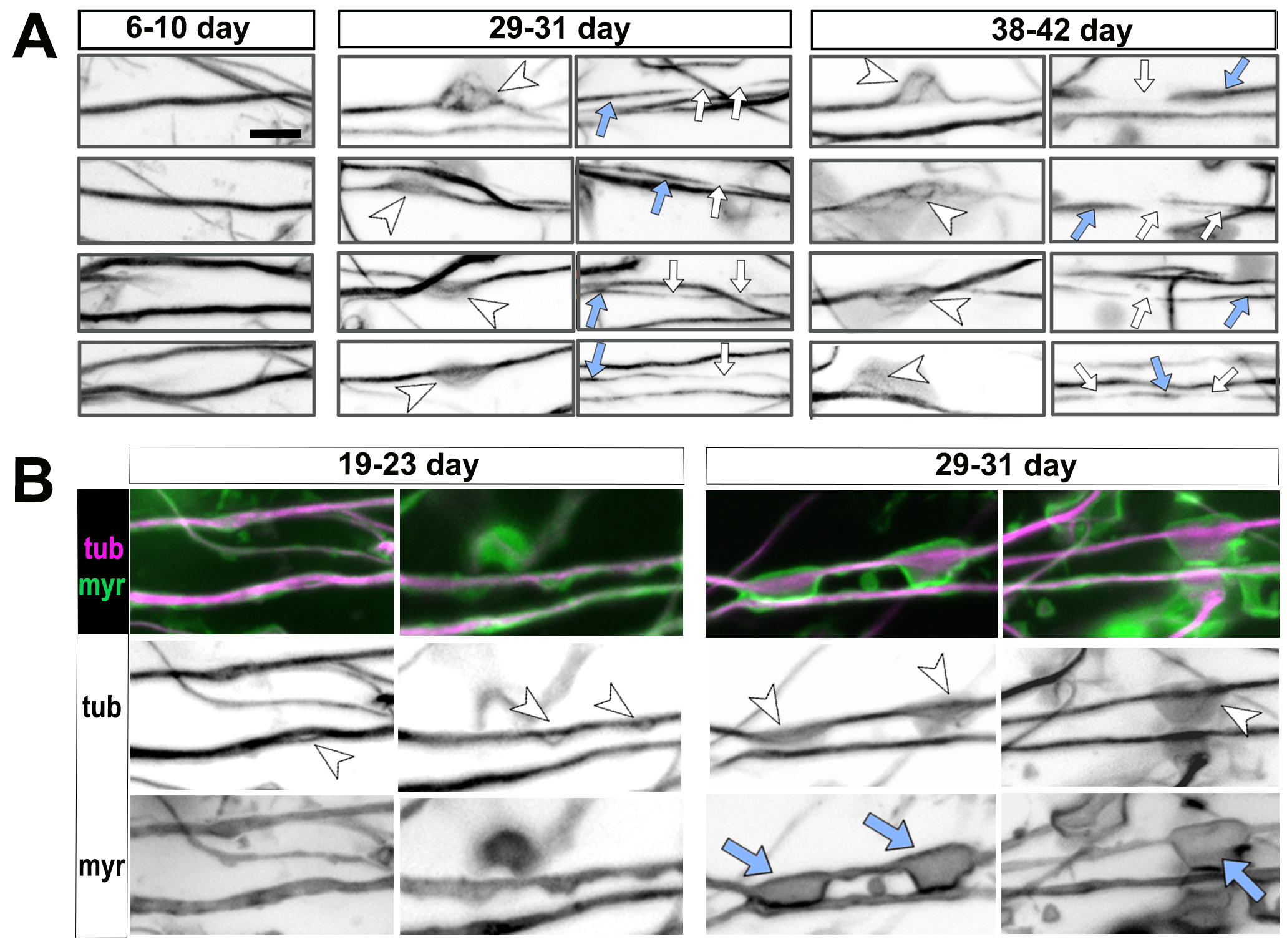

Supplement: S4 Fig — (A) MT bundles labelled with GFP-tagged α-tubulin (in inverted greyscale) from axons of T1 neuron, from specimens of different age groups: young (6–10 days old) or old (29–31 or 38–42 days old). Aged flies show MT unbundling (arrowheads) as well as breaks and thinning of MTs (white arrows) compared to normal/intact stretches of MTs, blue arrows. (B) MT bundles from axons of T1 neuron, from specimens labelled with GFP-tagged α-tubulin (tub, magenta) and the plasma membrane marker myr-Tom (myr, green) show MT unbundling (arrowheads) in 19–23 days specimens. Examples of typical axonal swelling which were quantified in this study (blue arrows) can be observed in 29–31 days old specimens. At age 5–10 days, 20% of swellings contain unbundled disorganised microtubules; at age 19–23, 21%, at age 29–31, 40% and at age 38–42, 56% of the swellings contain unbundle disorganised microtubules. Scale bar = 4 μm. (TIF) [file pbio.3002504.s004.tif]

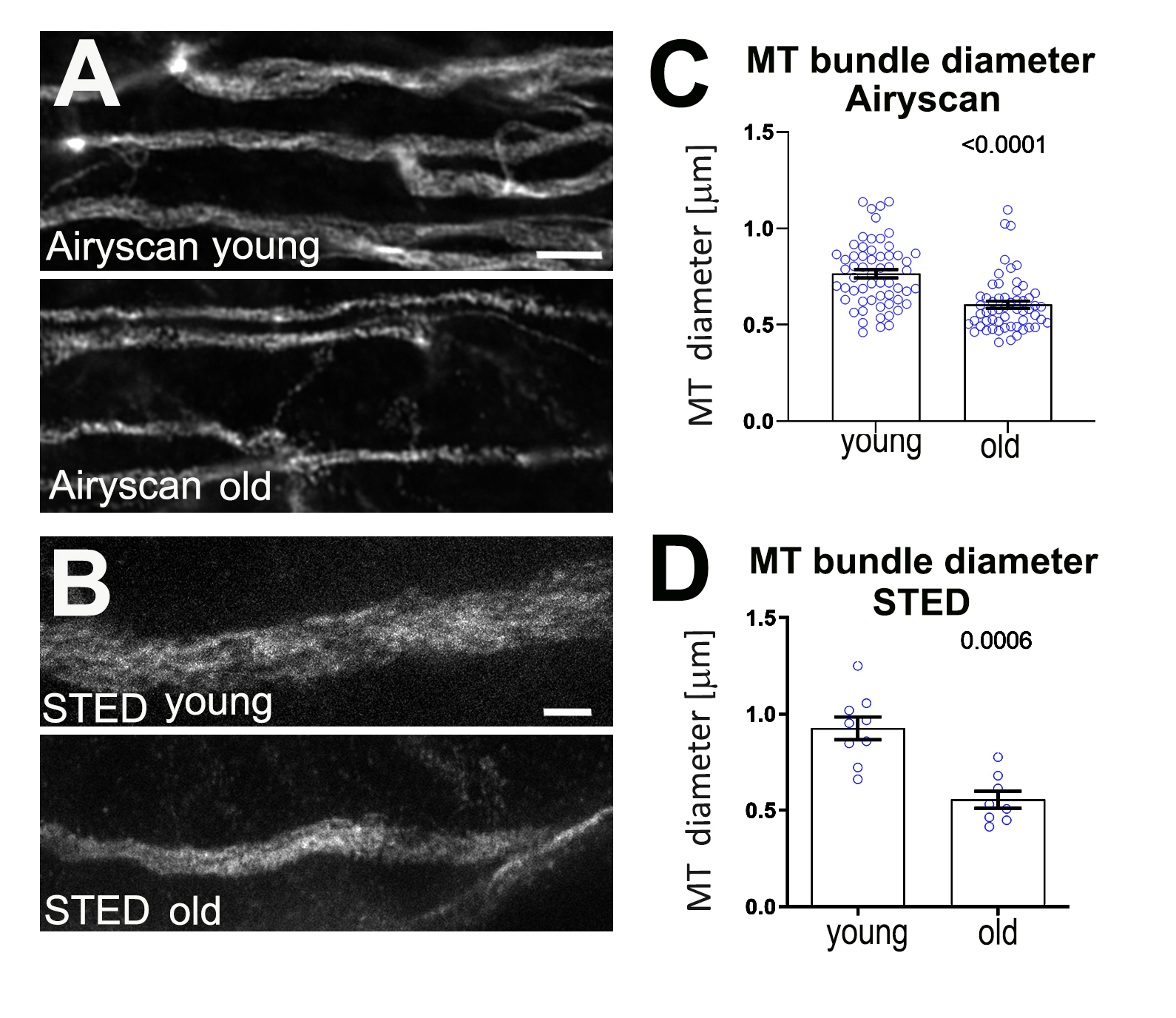

Supplement: S5 Fig — (A and B) MT axonal bundles from T1 neurons from young (8–10 days old) and old (34–36 days old) specimens labelled with GFP-tagged α-tubulin were imaged using Airy scanning high-end confocal microscopy (A) or STED super-resolution microscopy (B). (C and D) Images obtained using both imaging techniques have been used to quantify the diameter of MT bundles (C with Airy scanning and D with STED). Results are presented as mean ± SEM with individual data points in blue. P-values obtained with Mann–Whitney test are indicated in each graph. Data points were taken from at least 13 specimens per age group for Airy Scanning and 3 specimens for STED. For detailed statistical values and genotypes, see Table D within the S1 Tables. All the single values are provided in the S1 Datapoints. Scale bar in A represents 4 μm and in B 0.5 μm. (TIF) [file pbio.3002504.s005.tif]

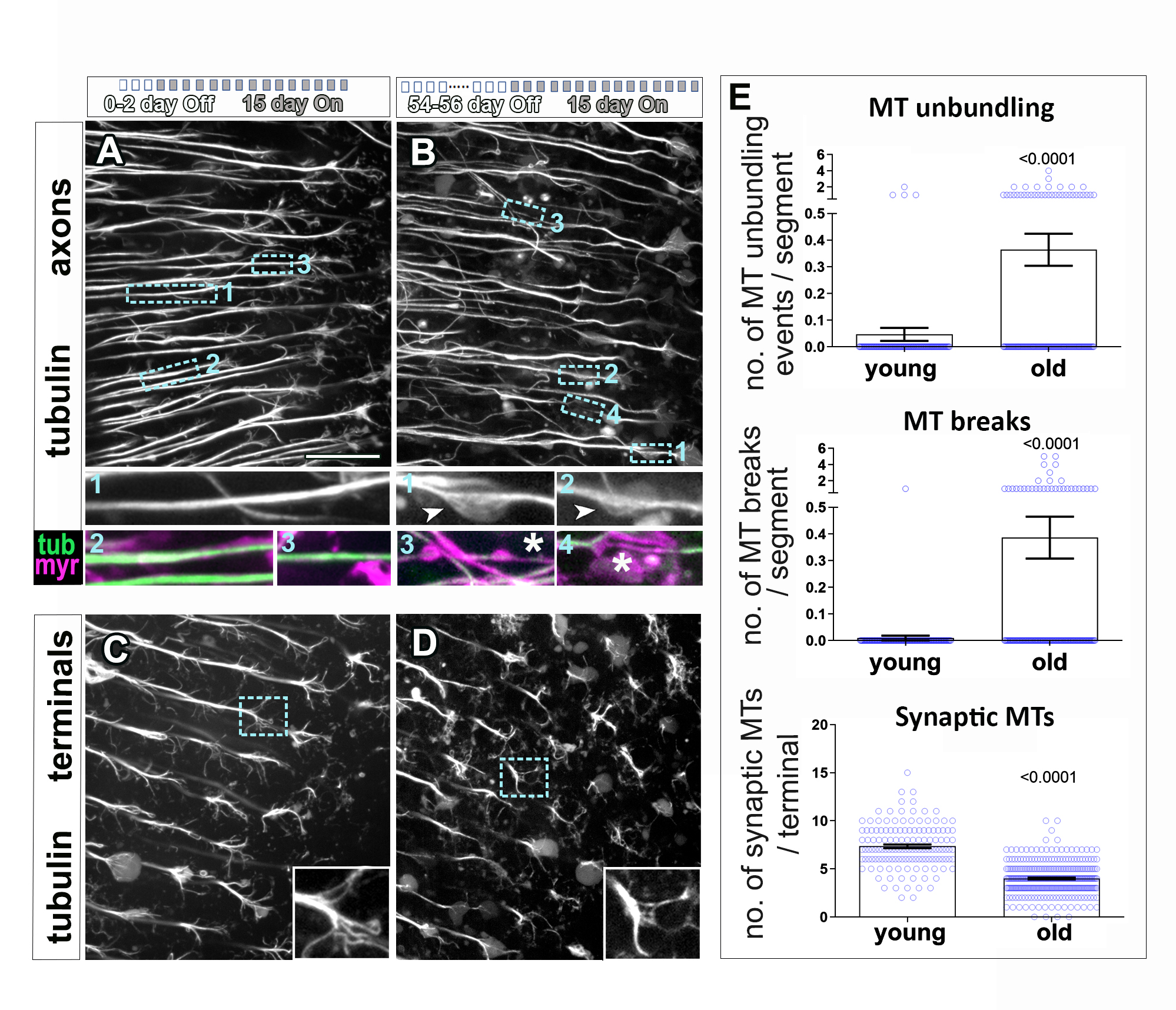

Supplement: S6 Fig — (A–D) T1 axons (A and B) and synaptic terminals (bottom and D) in the medulla of flies with their MTs labelled with GFP-tagged α-tubulin (tubulin, in greyscale images and green in insets) and the plasma membrane marker myr-Tom (myr, magenta), using the UAS/Gal4/Gal80ts system. Gene expression is induced by the shift of temperature from 18°C to 29°C. Flies were kept at 18°C throughout development and adult life until the last 15 days before imaging, at which point they were shifted to 29°C to induce gene expression (15 days expression was used to achieve sufficient MT labelling). Young specimens (A and C; 15–17 day old flies with 0–2 days at 18°C “Off” + 15 days at 29°C “On”) are compared to old specimens (B and D; 69–71 days old flies with 54–56 days at 18°C “Off”+ 15 at 29°C “On”). In A and B, cyan encircled boxes are shown 3.7-fold magnified below with old flies in B, showing MT unbundling (insets 1 and 2 in B) as well as breaks and thinning of MTs (insets 3 and 4 in B) compared to young axons. (D) Old flies also show a reduction of splayed MTs in synaptic terminals when compared to young controls in C (boxed areas shown as 2-fold magnified insets). (E) Quantifications of phenotypes shown in A–D, with young versus old indicated on the X-axes; data points are shown as blue circles and as mean ± SEM; p-values obtained via Mann–Whitney tests are indicated above. Data were taken from a minimum of 5 specimens per age group. For detailed statistical values and genotypes, see Table E within the S1 Tables. All the single values are provided in the S1 Datapoints. Scale bar = 10 μm. (TIF) [file pbio.3002504.s006.tif]

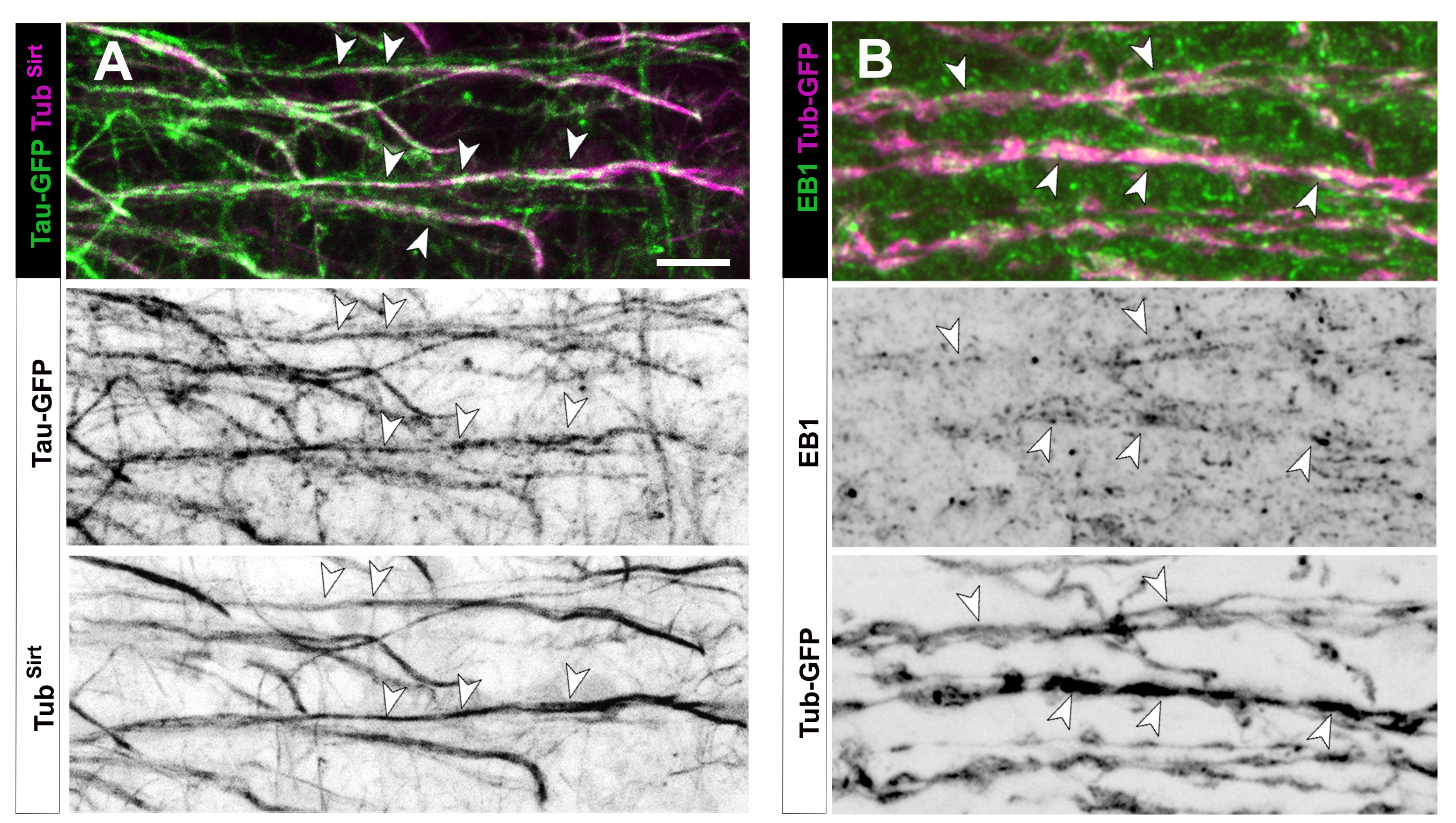

Supplement: S7 Fig — (A) Axonal region of the medulla from a 5–7 days old specimen labelled with endogenous GFP-tagged Tau (green in coloured image and in inverted greyscale image) and the live cell MT probe Sirt-Tubulin (magenta in coloured image and in inverted greyscale image), showing colocalisation of Tau to MTs (arrowheads in A). (B) Axonal region of the medulla from a 3- to 5-day-old specimen labelled with anti-EB1 (green in coloured image and in the inverted greyscale image) and expressing GFP-tagged α-tubulin in T1 neurons (magenta in coloured image and in the inverted greyscale image) showing EB1 signal colocalising to T1 MTs (arrowheads in B). Scale bar in A = 10 μm. (TIF) [file pbio.3002504.s007.tif]

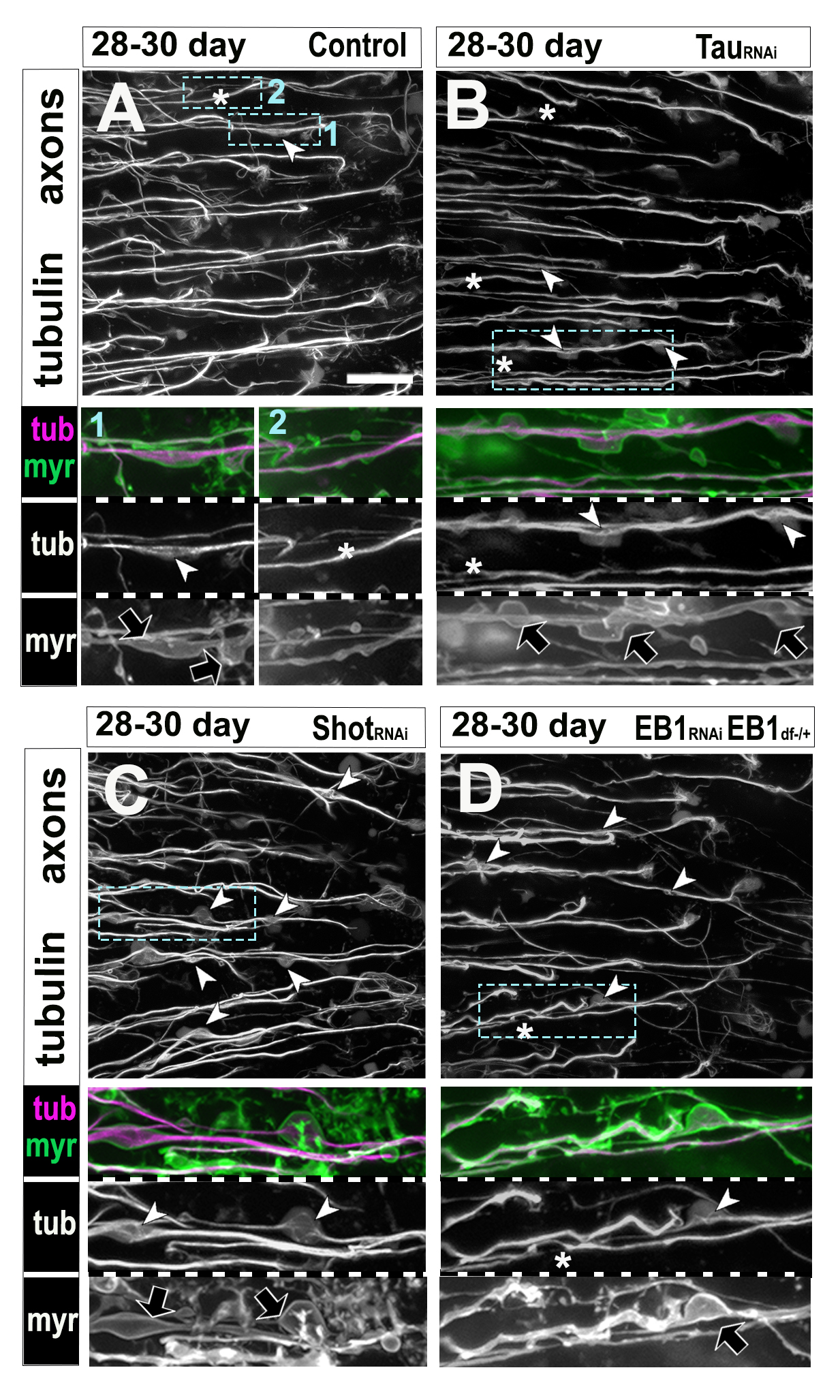

Supplement: S8 Fig — (A–D) Representative images of T1 axons in the medulla of 28–30 days old flies labelled with GFP-tagged α-tubulin (tubulin, greyscale and magenta in insets) and the plasma membrane marker myr-Tom (myr, green in insets). Aged neurons in the absence (A) or presence of Tau knockdown (TauRNAi in B), Shot knockdown (ShotRNAi C), and EB1 knockdown in a deficiency heterozygous background (EB1RNAi; EB1+/Df in D). Knockdown of the above MT regulators enhance phenotypes in ageing neurons, comprising axon swellings often displaying MT unbundling (arrowheads) and MT breaks (asterisks); boxed areas shown as 2-fold magnified double/single-channel images below. Boxed area shown as 2-fold magnified double/single-channel images below. Scale bar in A = 10 μm. (TIF) [file pbio.3002504.s008.tif]

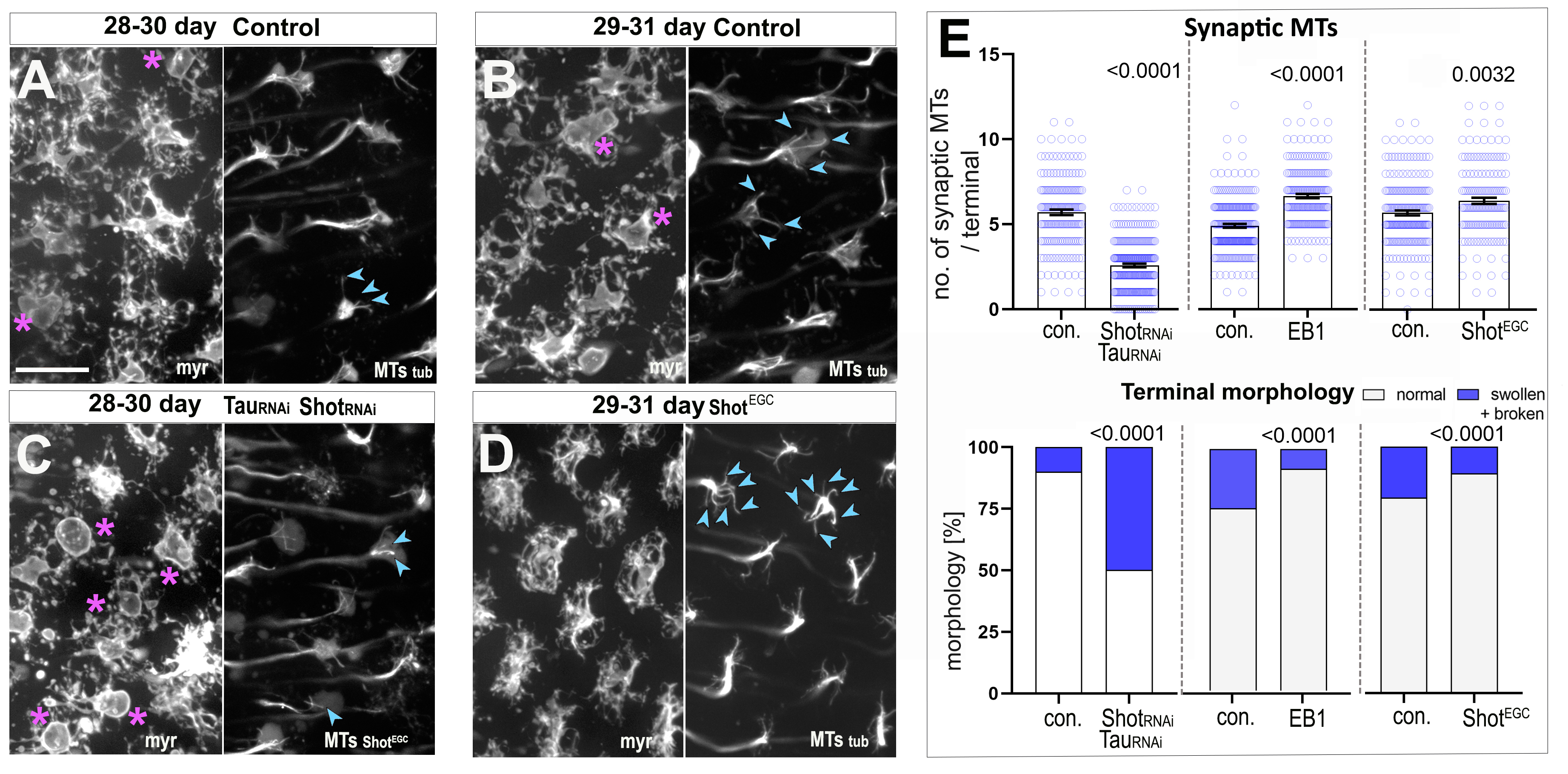

Supplement: S9 Fig — (A–D) Terminals of the T1 medulla projections from old specimens (28–30 days A and C; 29–31 days B and D) with the plasma membrane labelled with myr-Tom (myr) and their MTs with GFP-tagged α-tubulin (A–C) or GFP-tagged ShotEGC (D). Ageing phenotypes at the synaptic terminals, including a decrease in synaptic MTs (arrowheads) and an increase in broken and swollen terminals (asterisks), are enhanced in the presence of combined Tau and Shot knockdowns (TauRNAi ShotRNAi in C). The same ageing phenotypes are suppressed by the expression of ShotEGC (D). (E) Quantifications of phenotypes shown in A to D plus conditions of EB1 ectopic expression. Specific conditions are indicated below the X-axes. In the upper graphs, data points are shown as blue circles and as mean ± SEM; p-values obtained via Mann–Whitney test are indicated above. For terminal morphology, data are represented as distribution of normal versus swollen/broken synapses; significance obtained via Chi-square test is indicated above. Data were taken from a minimum of 14 specimens per group. For detailed statistical values and genotypes, see Table J within the S1 Tables. All the single values are provided in the S1 Datapoints. Scale bar in A represents 10 μm in A–D. (TIF) [file pbio.3002504.s009.tif]

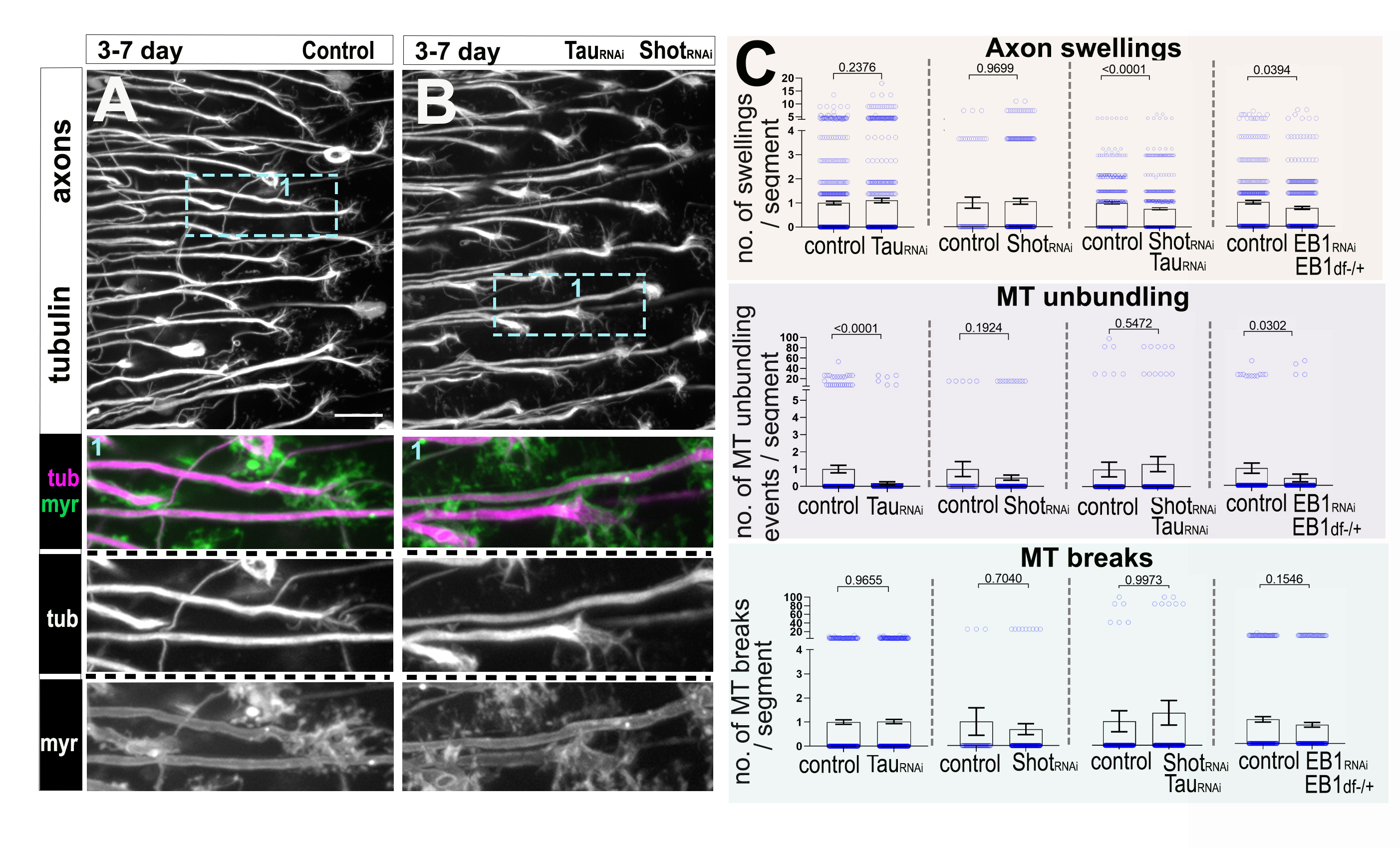

Supplement: S10 Fig — (A and B) Representative images of T1 axons in the medulla of 3–7 days old flies labelled with GFP-tagged α-tubulin (tubulin, greyscale and magenta in insets) and the plasma membrane marker myr-Tom (myr, green in insets). Images show specimens in the absence (A) or presence of combined Tau and Shot knockdowns (TauRNAi ShotRNAi in B). MTs axonal bundles maintain their diameter and organisation, and axons lack swellings and areas of decreased diameter in the presence of knockdowns; boxed area shown as 2-fold magnified double/single-channel images below. (C) Quantifications of phenotypes shown in A and B plus conditions of further knockdowns for Tau (TauRNAi), Shot (ShotRNAi), and EB1 in an EB1 heterozygous background (EB1RNAi; EB1+/Df); specific knockdowns are indicated below the X-axes. Data points are shown as blue circles and as mean ± SEM; p-values obtained via Mann–Whitney tests are indicated above. Data were taken from a minimum of 13 specimens per age group and condition with the exception of 8 specimens for Shot. For detailed statistical values and genotypes, see Table K within the S1 Tables. All the single values are provided in the S1 Datapoints. Boxed area shown as 2-fold magnified double/single-channel images below. Scale bar in A = 10 μm. (TIF) [file pbio.3002504.s010.tif]

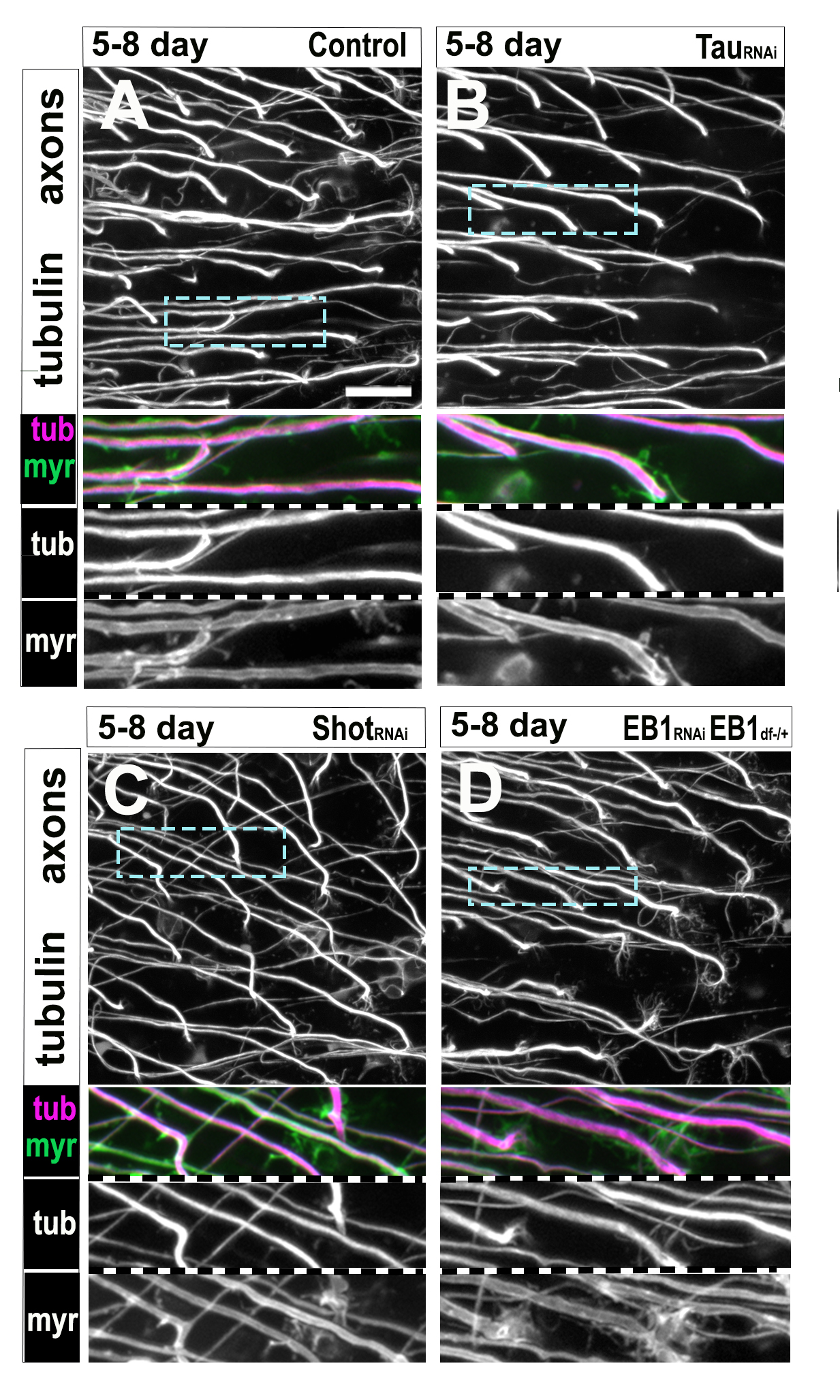

Supplement: S11 Fig — (A, D) Representative images of T1 axons in the medulla of 5–8 days old flies labelled with GFP-tagged α-tubulin (tubulin, greyscale and magenta in insets) and the plasma membrane marker myr-Tom (myr, green in insets). Images show young specimens in the absence (A) or presence of Tau knockdown (TauRNAi in B), Shot knockdown (ShotRNAi in C), and EB1 knockdown in a deficiency heterozygous background (EB1RNAi; EB1+/Df in D). In the presence of knockdowns, the diameter and organisation of MTs axonal bundles are unaltered, and axons lack swellings and their diameter are maintained; boxed area shown as 2-fold magnified double/single-channel images below. Scale bar in A represents 10 μm in all images. (TIF) [file pbio.3002504.s011.tif]

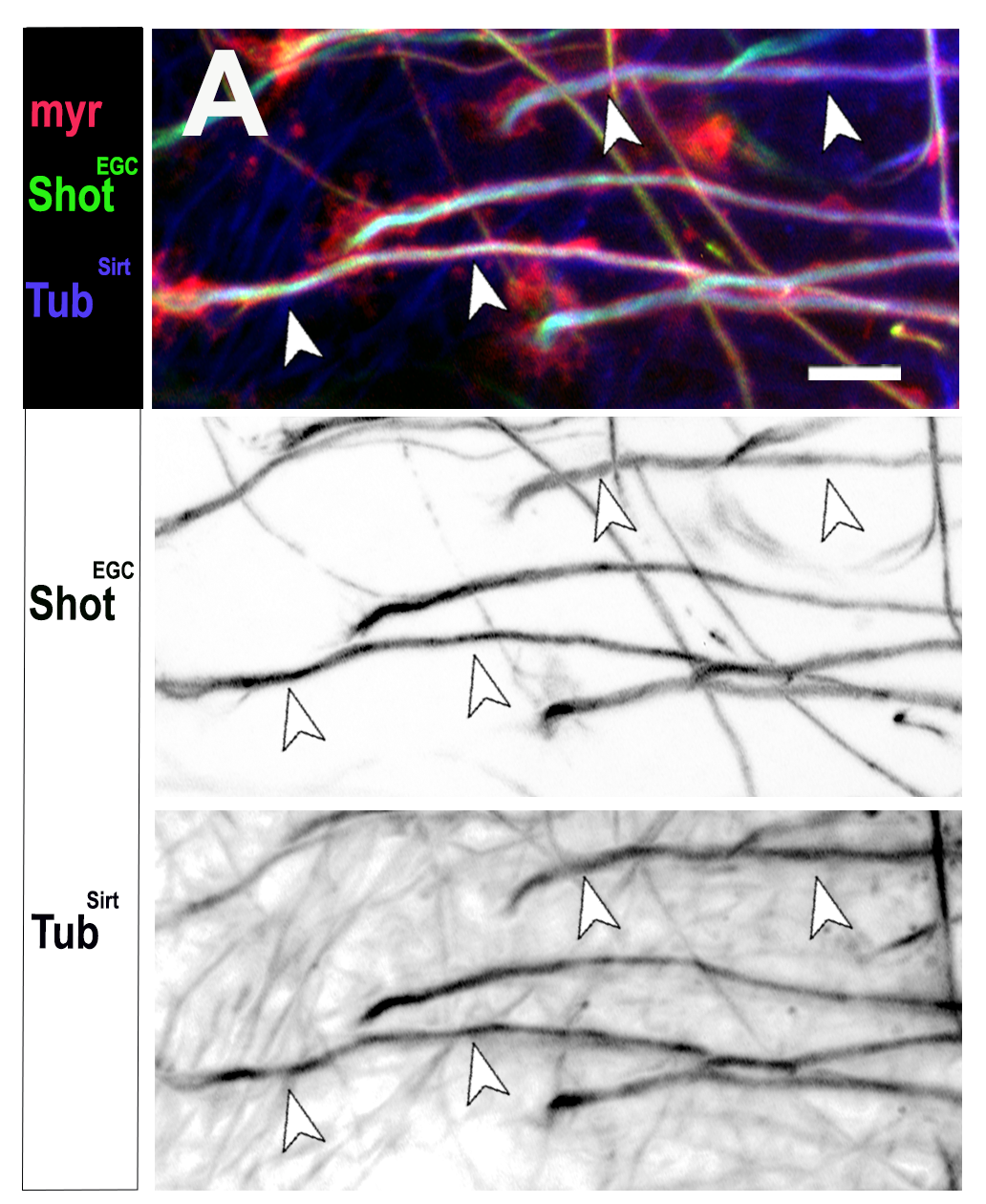

Supplement: S12 Fig — (A) Axonal region of the medulla from a 5–7 days old specimen expressing the GFP-tagged ShotEGC fragment and the plasma membrane marker myr-Tom (myr) in T1 neurons and labelled with the live cell MT probe Sirt-Tubulin (Myr in red, ShotEGC in green and TubulinSirt in blue, single channels are shown in inverted greyscale images). ShotEGC colocalises to the shaft of MTs (some examples are indicated with arrowheads). Scale bar in A = 10 μm. (TIF) [file pbio.3002504.s012.tif]

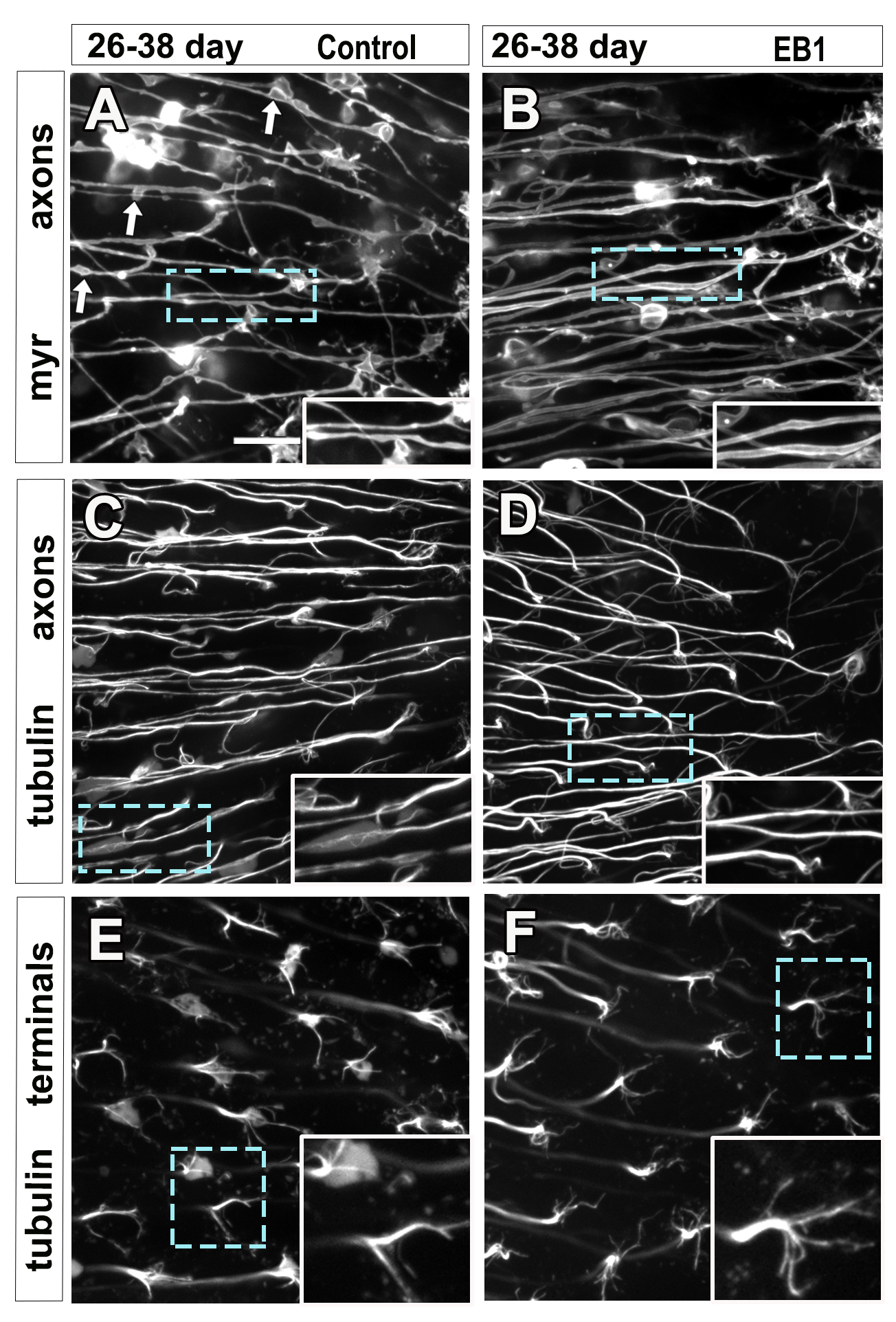

Supplement: S13 Fig — (A–F) T1 axons in the medulla are labelled with the plasma membrane marker myr-Tom (myr, greyscale in A and B, age of specimens between 35 and 38 days old) or with GFP-tagged α-tubulin (tubulin, age of specimens between 26 and 28 days old). Ageing phenotypes including axon swellings (arrows in A), axon thinning (inset in A), MT unbundling (inset in C), and sparse synaptic MTs (inset in E) can be observed in old specimens, but are absent upon T1-specific expression of EB1 (B, D, F). Scale bar in A–D represents 10 μm, and 14 μm in E and F. (TIF) [file pbio.3002504.s013.tif]

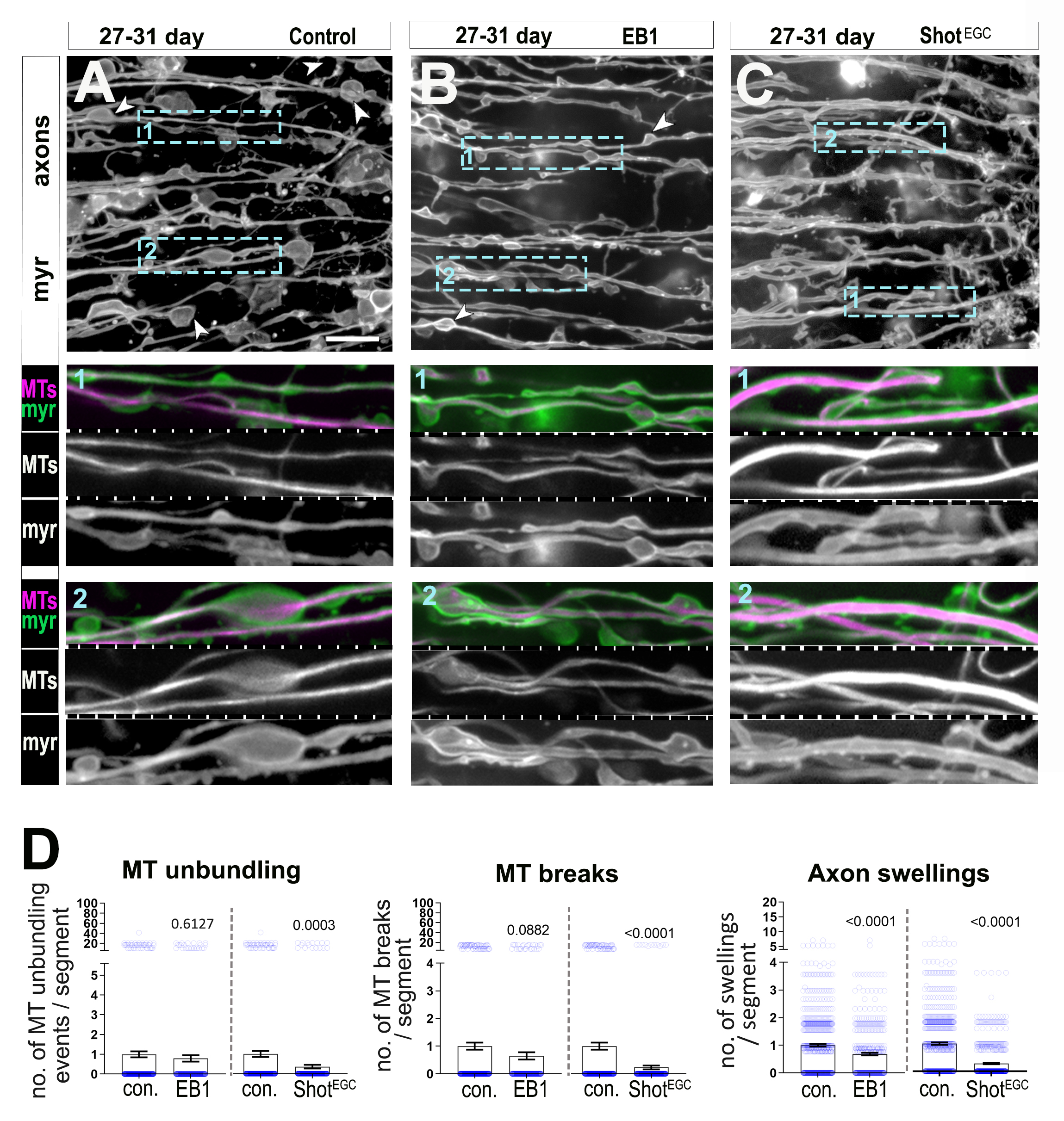

Supplement: S14 Fig — (A–C) T1 axons in the medulla of old specimens of 27–33 days old control brains (A), overexpressing EB1 (B), or ShotEGC (C). Brains are labelled with the plasma membrane marker myr-Tom (myr, greyscale images in A–C, and green in double-channel images) and with the MT markers GFP-tagged α-tubulin, in A and B, or GFP-tagged ShotEGC in C (MTs for both and in magenta in double-channel images). Using the UAS/Gal4/Gal80ts system, gene expression is induced after development by shifting newly eclosed flies from 18°C to 29°C. Ageing phenotypes including axon swellings, axon thinning (A), MT thinning (boxed area 1 in A), and MT unbundling (boxed area 2 in A) can be observed in old specimens and specimens overexpressing EB1 post-development, but are absent upon post-developmental expression of ShotEGC in adult T1 neurons (A and B compared to C, boxed areas 1 and 2 shown as 2-fold magnified inset were MTs are labelled with GFP-tagged α-tubulin in A and B, or GFP-tagged ShotEGC in B). (C) Quantifications of phenotypes in old specimens shown in A and B, plus conditions of post-development expression of EB1 in old specimens in C (27–33 days old). Specific conditions are indicated below the X-axes; data points are shown as blue circles and as mean ± SEM; p-values obtained via Mann–Whitney test are indicated above. Data were taken from a minimum of 23 specimens per group. For detailed statistical values and genotypes, see Table M within the S1 Tables. All the single values are provided in the S1 Datapoints. Boxed area shown as 2-fold magnified double/single-channel images below. Scale bar in A represents 10 μm. (TIF) [file pbio.3002504.s014.tif]

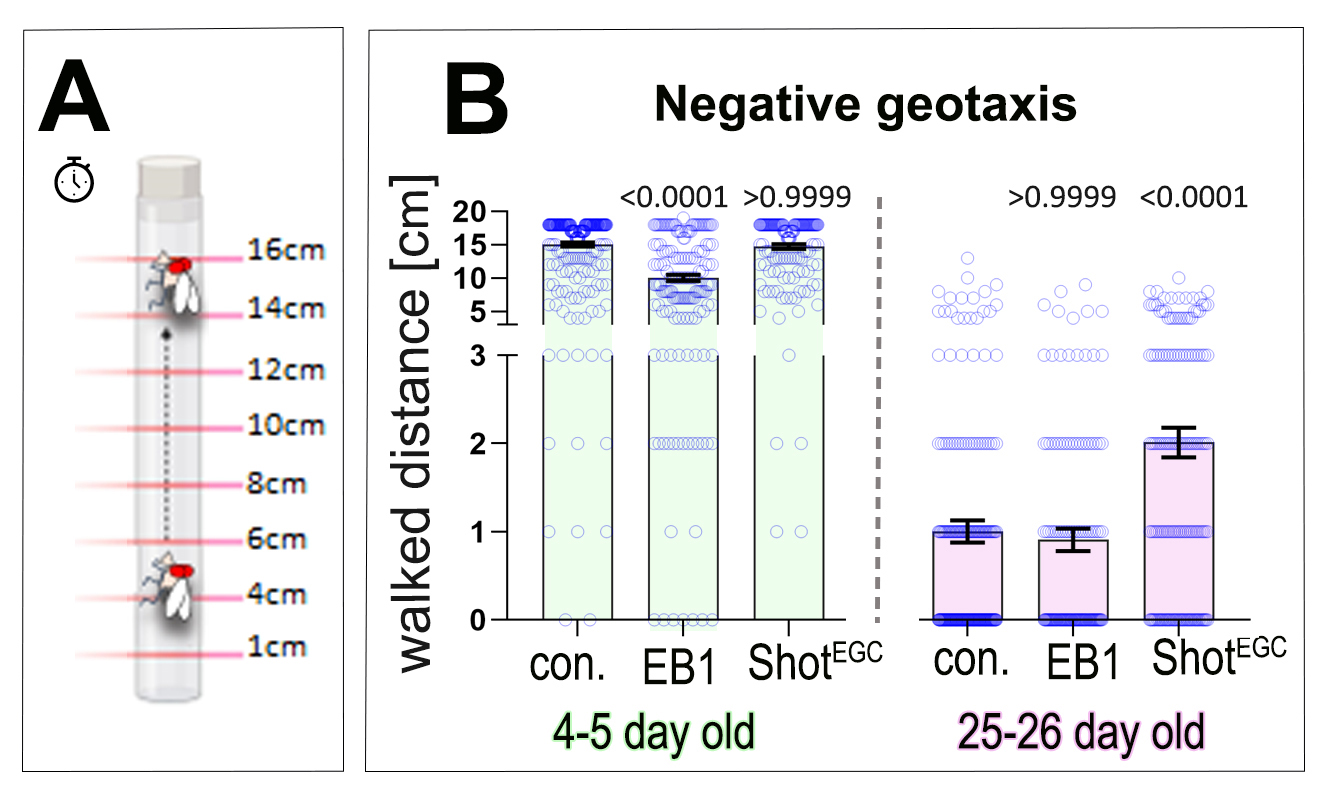

Supplement: S15 Fig — (A) Representation of the negative geotaxis walking assays with flies allowed to walk upwards for 15 s, in a narrow-graduated cylinder. (B) Quantifications of the distance walked by flies in 15 s at 2 different ages 4–5 days old and 25–26 days old. Specific conditions are indicated below the X-axes, and include control flies or flies expressing either ShotEGC or EB1 in adulthood with the pan-neuronal elav-Gal4 driver using the UAS/Gal4/Gal80ts system (gene expression is induced after development is completed by shifting newly hatched flies from 18 to 29°C, controls are treated with the same regime but are lacking the transgene). Data points are shown as blue circles and as mean ± SEM. P-values obtained via Kruskall–Wallis ANOVA multiple comparison tests are indicated in the graphs. Data were taken from a minimum of 140 specimens per group. For detailed statistical values and genotypes, see Table O within the S1 Tables. All the single values are provided in the S1 Datapoints. (TIF) [file pbio.3002504.s015.tif]

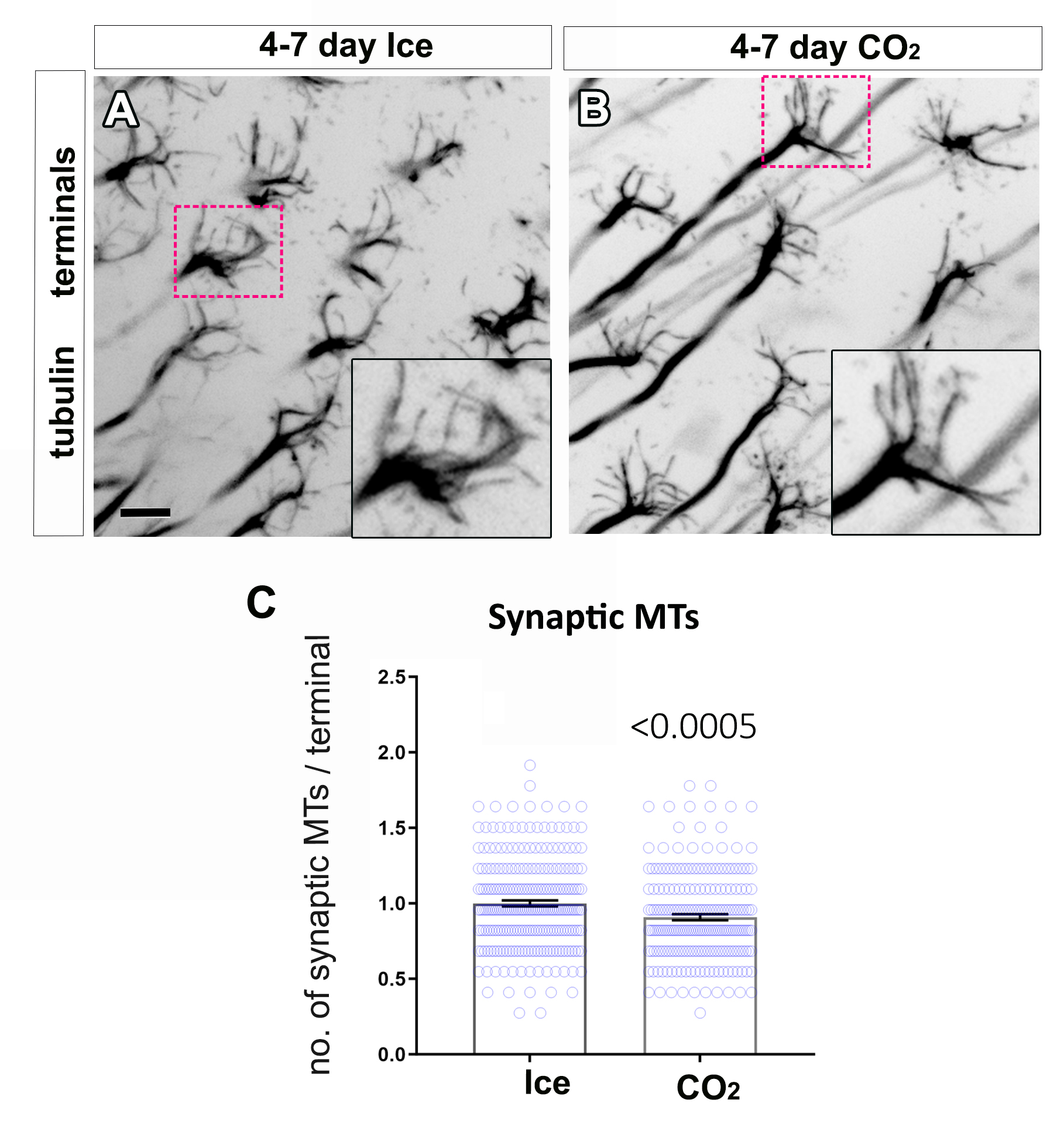

Supplement: S16 Fig — (A and B) Synaptic terminals with labelled MTs with GFP-tagged α-tubulin (tubulin), from 4–7 days old specimens which have been anaesthetised for 90 s, either by incubation on ice (A) or exposure to CO2 (B) (boxed areas shown as 2-fold magnified insets). (C) Quantifications of synaptic MTs from similar images shown in A and B, with type of anaesthesia treatment indicated below the X-axes; data points are shown as blue circles and as mean ± SEM; p-values obtained via Mann–Whitney test. For detailed statistical values and genotypes, see Table P within the S1 Tables. All the single values are provided in the S1 Datapoints. A scale bar can be found in A, bottom left (A and B, 10 μm). (TIF) [file pbio.3002504.s016.tif]

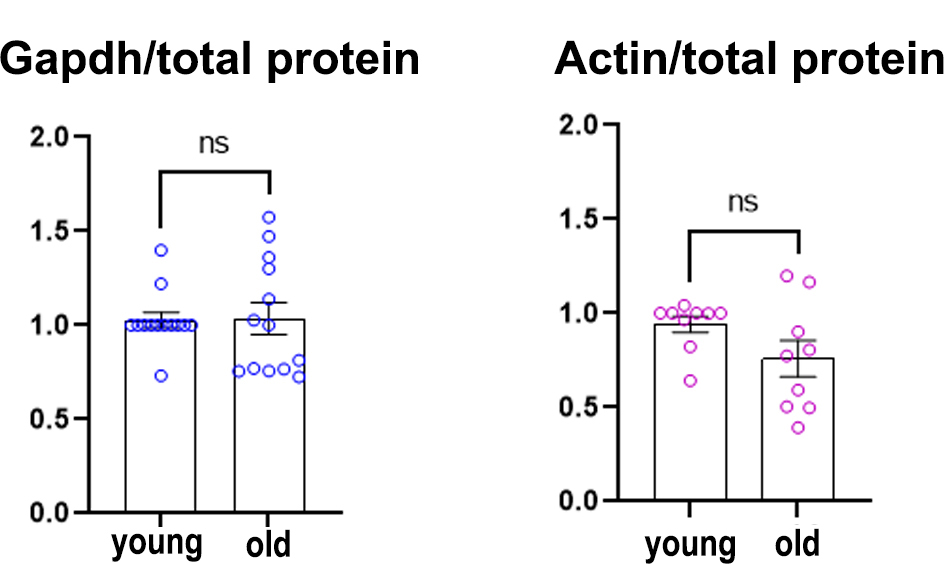

Supplement: S17 Fig — Quantifications of total Gapdh and Actin from western blots and relative to the total protein are compared between young (4–9 days at 29°C) and old (29–32 days at 29°C) specimens, demonstrating there is no significant difference between young and old. The graphs depict data points that represent independent lysates, and mean bars ± SEM; p-values obtained via Mann–Whitney test. Values have been normalised to the correspondent young for each experiment. For detailed statistical values and genotypes, see Table Q within the S1 Tables. All the single values are provided in the S1 Datapoints, and all western blots can be found in the S1 Western Blots. (TIF) [file pbio.3002504.s017.tif]

Figure 5.C

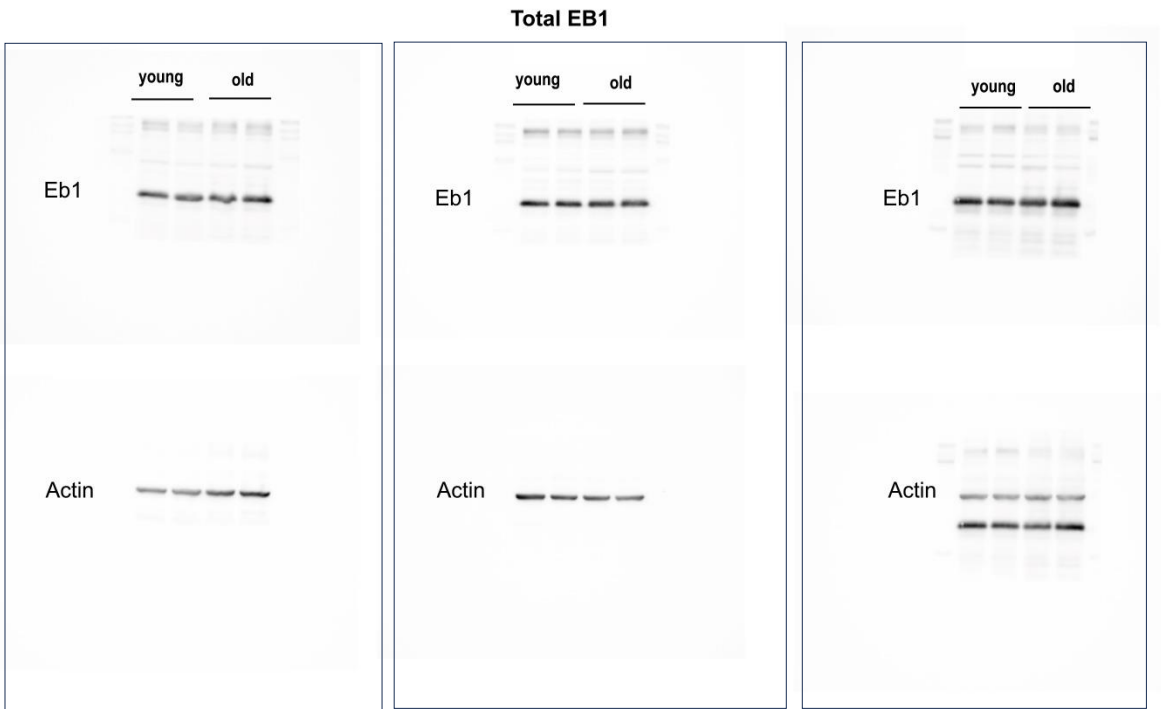

Figure 5.D and H

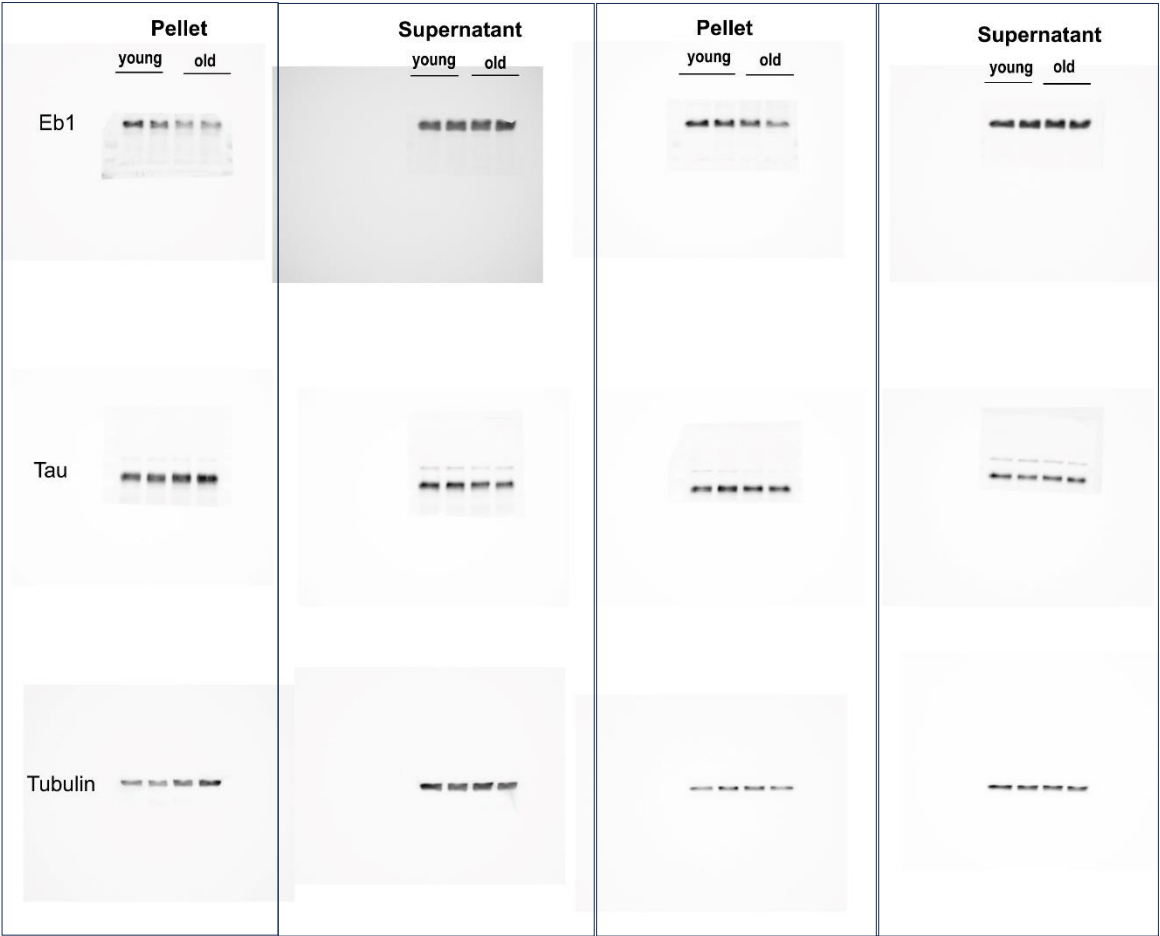

Figure 5.D and H

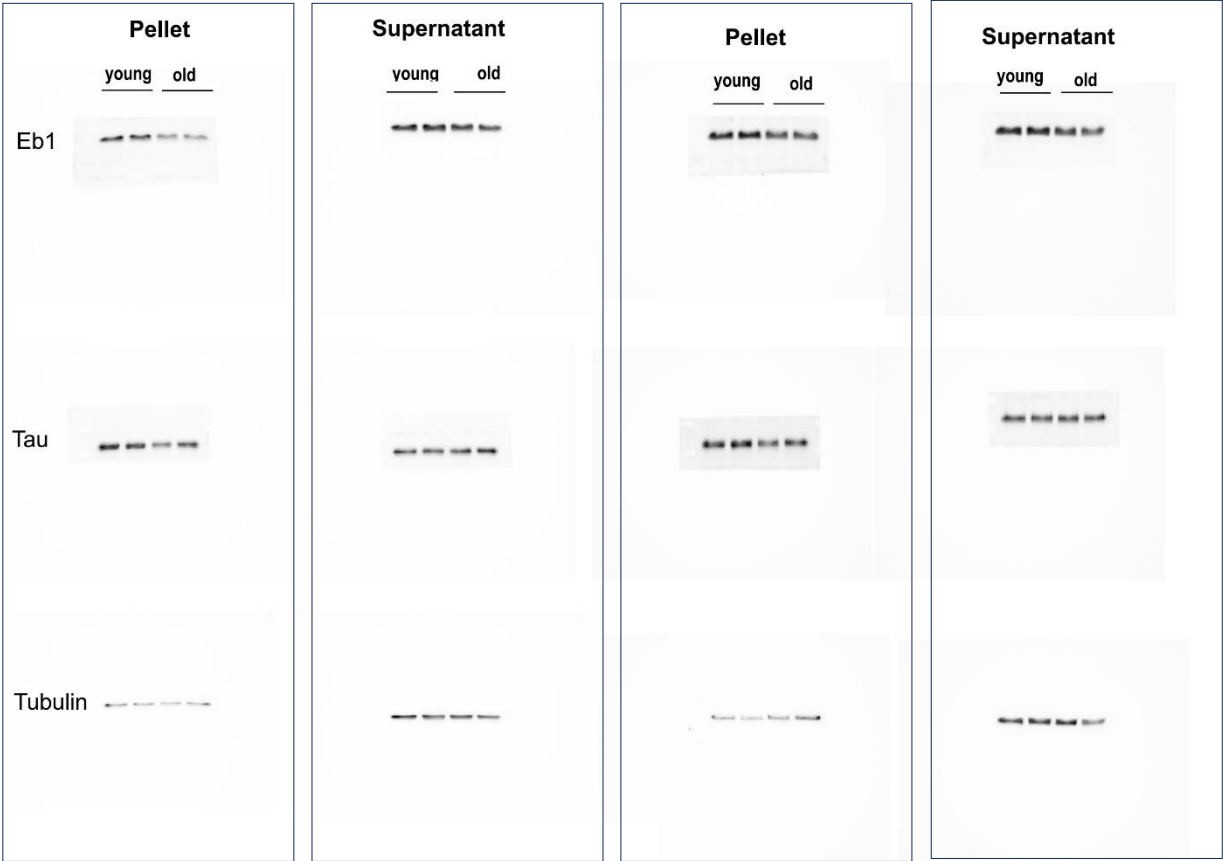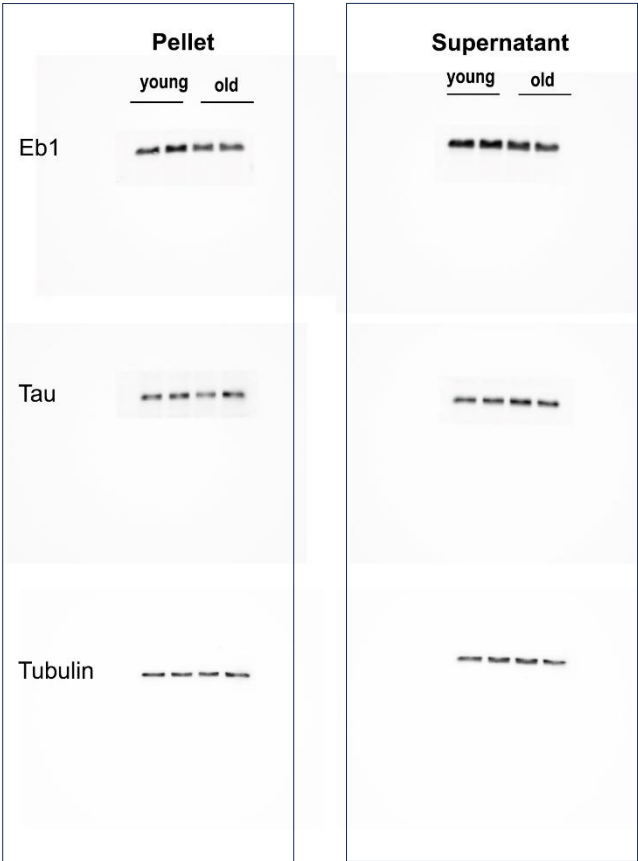

Figure 5.G

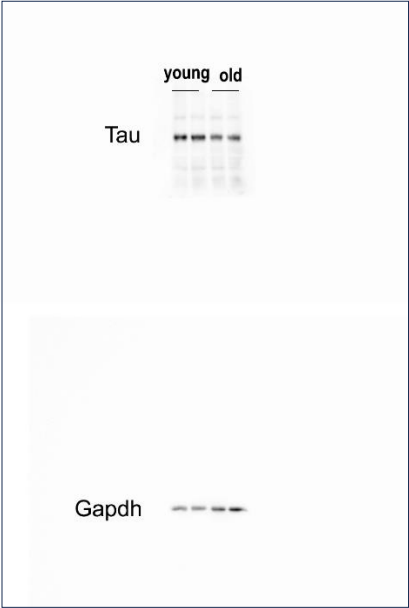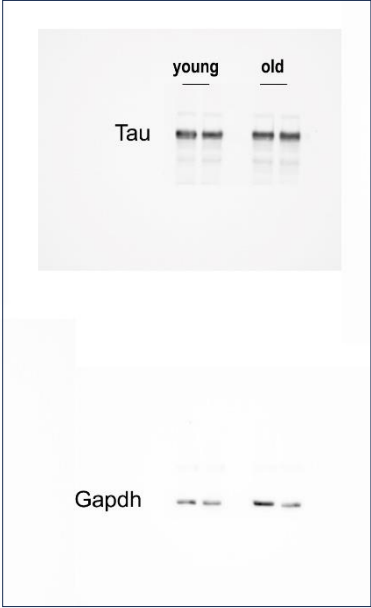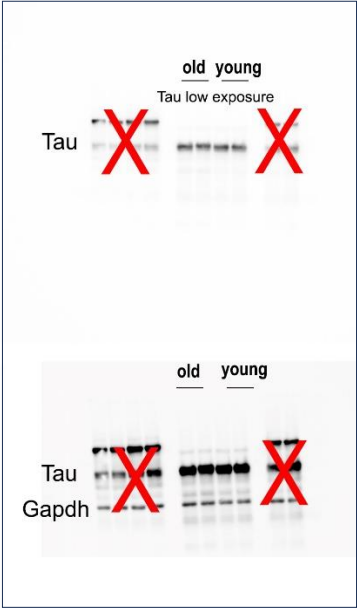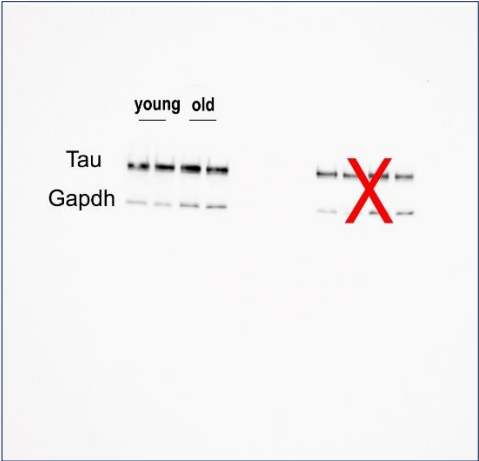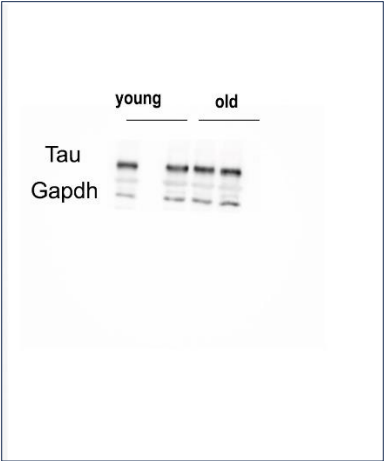

### S16 Figure

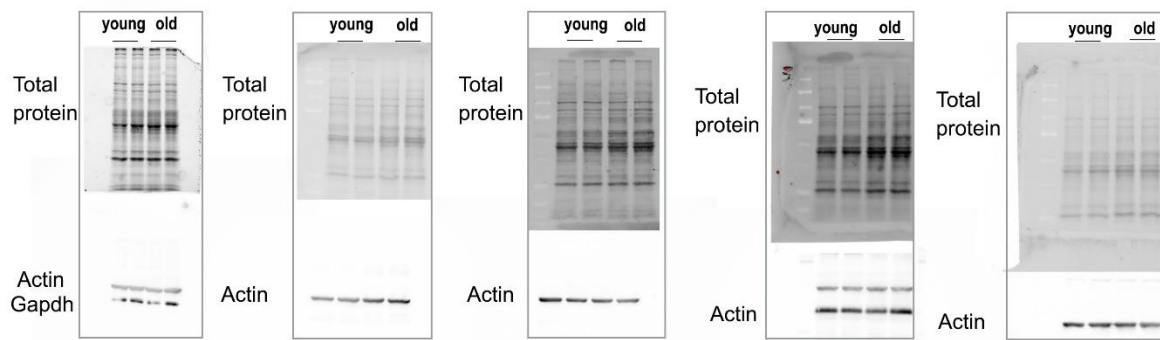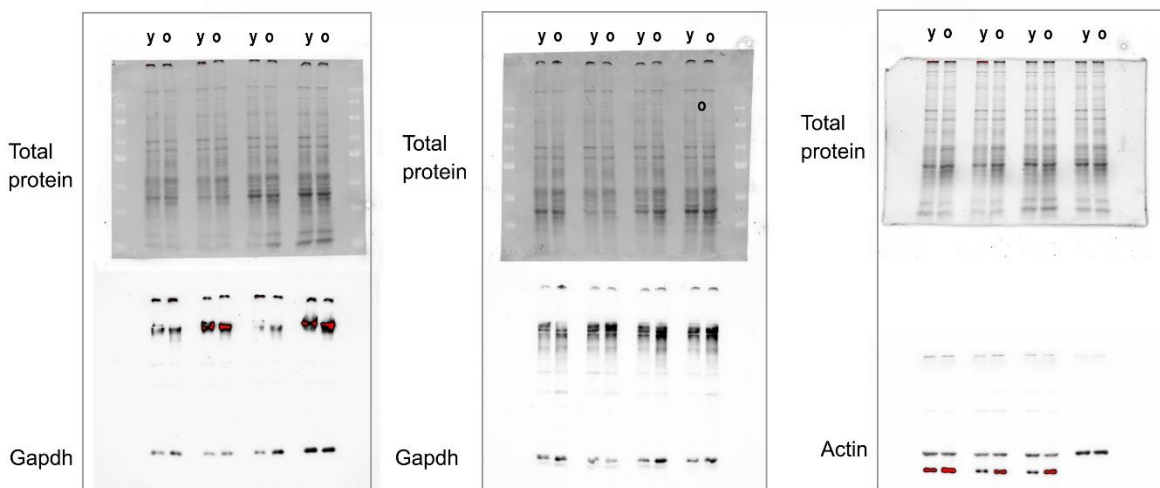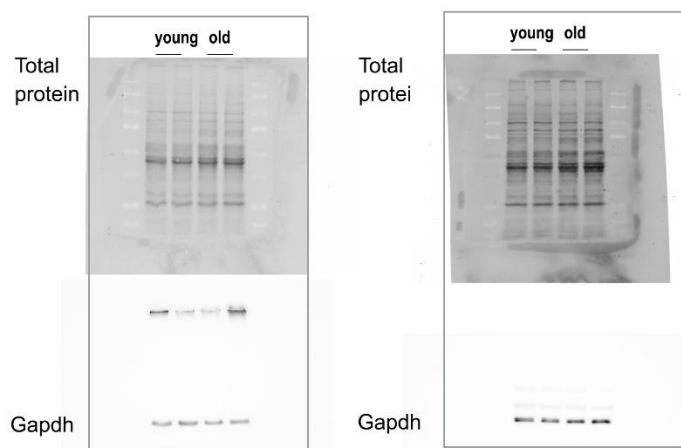

Supplement: S1 Western Blots — Compilation of western blots used for quantification in Figs 5 and S16. (PDF) [file pbio.3002504.s020.pdf]
