# Supplementary material for: Neuronal ageing is promoted by the decay of the microtubule cytoskeleton
Source: PLoS Biol. 2024 Mar 13;22(3):e3002504. doi: 10.1371/journal.pbio.3002504 (PMC10962844; doi:10.1371/journal.pbio.3002504)
Supplement: S1 Tables — The statistical details and specific genotypes for all qualifications in this study can be found in this collection of tables. (PDF) [file pbio.3002504.s018.pdf]

TABLES

Genotype	W^{1118};*UAS-myr-tdTomato /+;UAS-GFP-α-tubulin84B, GMR31F10-Gal4/+*			
Age (days)	**5-10**	**19-23**	**29-31**	**38-42**
	**Axon diameter**			
Number of values (axons)	145	143	104	241
Number specimens	10	14	11	29
Mean	0.7094	0.6716	0.4997	0.4878
Std. Deviation	0.1546	0.1	0.07939	0.1029
Std. Error of Mean	0.01284	0.008363	0.007785	0.00663
	**Axon swellings**			
Number of values (µm of axon)	303	303	240	319
Number specimens	24	22	24	28
Mean	0.007629	0.01310	0.01397	0.02427
Std. Deviation	0.01474	0.02056	0.02106	0.02773
Std. Error of Mean	0.0008466	0.001181	0.001359	0.001552
	**Terminal area**			
Number of values (terminals)	366	535	426	467
Number specimens	14	15	23	33
Mean	36.18	37.67	32.45	27.95
Std. Deviation	12.58	13.47	12.12	11.59
Std. Error of Mean	0.6575	0.5824	0.5874	0.5362
	**Terminal morphology**			
Number of values (terminals)	1238	826	1118	1696
Number of normal	826	814	1050	1501
Number of swollen+broken	32	12	68	195
Number specimens	14	15	23	31

Table A: Correspondent statistical details for Fig 1 "Axons and synaptic terminals within the *Drosophila* visual system deteriorate during ageing".

Age (days)	**5**	**14**	**23**	**31**
Genotype	W^{1118};*UAS-GFP-α-tubulin84B /+;UAS- RedStinger, GMR31F10-Gal4/+*			
	**T1 neurons**			
Number of values-specimens	12	5	10	11
Mean	140.8	230.2	200.6	241.0
Std. Deviation	39.38	40.55	41.04	38.26
Std. Error of Mean	11.37	18.14	12.98	11.54

Table B: Correspondent statistical details for S2 Fig "Absence of neuronal death amongst T1 neurons during ageing".

Age (days)	**3 days 18^{0}C + 4 days 29^{0}C**	**56 days 18^{0}C + 4 days 29^{0}C**
Genotype	*W^{1118};tub-Gal80^{ts}/ UAS-my-tdTomato;UAS-GFP-α-tubulin84B, GMR31F10-Gal4/+*	
	**Axon diameter**	
Number of values (axons)	66	85
Number specimens	5	8
Mean	0.7732	0.5885
Std. Deviation	0.1393	0.1052
Std. Error of Mean	0.01714	0.01141
	**Axon swellings**	
Number of values (µm of axon)	123	98
Number specimens	5	8
Mean	0.006378	0.01722
Std. Deviation	0.01307	0.02129
Std. Error of Mean	0.001178	0.002151
	**Terminal morphology**	
Number of values (terminals)	300	485
Number of normal	298	371
Number of swollen+broken	2	114
Number specimens	5	8

Table C: Correspondent statistical details for S3 Fig "The deterioration of T1 neurons during ageing is independent of temperature and marker expression".

Age (days)	**5-10**	**19-24**	**29-31**	**38-42**
Genotype	*W^{1118};UAS-myr-tdTomato;UAS-GFP-α-tubulin84B, GMR31F10-Gal4/+*			
	**MT unbundling**			
Number of values (axonal bundle segment)	562	713	616	264
Number specimens	22	23	17	17
Mean	0.08007	0.1445	0.1851	0.3030
Std. Deviation	0.3027	0.4404	0.4887	0.6034
Std. Error of Mean	0.01277	0.01649	0.01969	0.03714
	**MT breaks**			
Number of values (axonal bundle segment)	563	696	500	266
Number specimens	22	23	17	17
Mean	0.04263	0.1710	0.1420	0.5489
Std. Deviation	0.2022	0.4562	0.4629	1.046
Std. Error of Mean	0.008522	0.01729	0.02070	0.06413
	**Synaptic MTs**			
Number of values (terminal)	297	458	333	252
Number specimens	15	18	15	13
Mean	10.25	7.175	5.186	3.988

Std. Deviation	2.646	2.226	1.874	1.633

	**MT bundle diameter Airyscan**	
Age (days)	**8-10**	**34-36**
Number of values (axons)	57	57
Number specimens	14	13
Mean	0.7610	0.6040
Std. Deviation	0.1702	0.1433
Std. Error of Mean	0.02254	0.01897
	**MT bundle diameter STED**	
Age (days)	**3**	**3**
Number of values (axons)	9	8
Number specimens	3	3
Mean	0.9265	0.5552
Std. Deviation	0.1785	0.1255
Std. Error of Mean	0.05950	0.04438

Table D: Correspondent statistical details for Fig 2 and S5 Fig "MT aberration precedes axonal and synaptic decay during ageing"

Age (days)	**3 days 18^0C + 15 days 29^0C**	**56 days 18^0C + 15 days 29^0C**
Genotype	*W^{1118};tub-Gal80^{ts}/ UAS-myr-tdTomato;UAS-GFP-α-tubulin84B, GMR31F10-Gal4/+*	
	**MT unbundling**	
Number of values (axonal bundle segment)	108	132
Number specimens	6	8
Mean	0.04630	0.3636
Std. Deviation	0.2515	0.6910
Std. Error of Mean	0.02420	0.06014
	**MT breaks**	
Number of values (axonal bundle segment)	108	132
Number specimens	6	8
Mean	0.009259	0.3864
Std. Deviation	0.09623	0.9050
Std. Error of Mean	0.009259	0.07877
	**Synaptic MTs**	
Number of values (terminal)	108	132
Number specimens	6	8
Mean	7.350	3.994
Std. Deviation	2.285	1.643
Std. Error of Mean	0.1931	0.09044

Table E: Correspondent statistical details for S6 Fig "MT alterations during ageing are independent of the MT reporter and are specific for aged specimens".

Age (days)	30-35	
Genotype	*W^{1118};UAS-myr-tdTomato /+; UAS-GFP-α-tubulin84B, GMR31F10-Gal4/+*	*W^{1118};UAS-myr-tdTomato /Chico^{1}; UAS-GFP-α-tubulin84B, GMR31F10-Gal4/+*
	**Axon swellings**	
Number of values (axonal bundle segment)	536	629
Number specimens	28	28
Mean	0.998	0.551
Std. Deviation	1.880	1.278
Std. Error of Mean	0.08119	0.05098
	**MT breaks**	
Number of values (axonal bundle segment)	563	629
Number specimens	28	28
Mean	0.998	0.630
Std. Deviation	1.676	1.222
Std. Error of Mean	0.070	0.048
	**MT unbundling**	
Number of values (axonal bundle segment)	555	620
Number specimens	28	28
Mean	0.998	0.462
Std. Deviation	4.255	3.315
Std. Error of Mean	0.180	0.1331
	**Axon diameter**	
Age (days)	**30-35**	
Number of values (segment)	128	170
Number specimens	14	16
Mean	1	1.2
Std. Deviation	0.201	0.1918
Std. Error of Mean	0.017	0.014
	**Synaptic MTs**	
Age (days)	**30-35**	
Number of values (terminal)	756	785
Number specimens	25	28
Mean	4.438	6.413
Std. Deviation	1.599	1.751
Std. Error of Mean	0.05815	0.06250

Table F: Correspondent statistical details for Fig 3 “A mutation in the insulin receptor substrate CHICO, known to increases lifespan, improves neuronal ageing hallmarks and MT decay”.

	Ratio GFP/Tom	
Age (days)	**3 days 18^{0}C + 4 days 29^{0}C**	**56 days 18^{0}C + 4 days 29^{0}C**
Genotype	*W^{1118};tub-Gal80^{ts}/ UAS-myr-tdTomato*; *UAS-GFP-α-tubulin84B, GMR31F10-Gal4/+*	
Number of values (axons)<br>Number specimens<br>Mean<br>Std. Deviation<br>Std. Error of Mean	127<br>5<br>1.848<br>0.4595<br>0.04078	200<br>7<br>1.076<br>0.2338<br>0.01653
	**Ratio GFP/Tom**	
Age (days)	**3 days 18^{0}C + 15 days 29^{0}C**	**56 days 18^{0}C + 15 days 29^{0}C**
Genotype	*W^{1118};tub-Gal80^{ts}/ UAS-myr-tdTomato*; *UAS-GFP-α-tubulin84B, GMR31F10-Gal4/+*	
Number of values (axons)<br>Number specimens<br>Mean<br>Std. Deviation<br>Std. Error of Mean	126<br>6<br>1.701<br>0.5551<br>0.04946	225<br>8<br>1.352<br>0.4969<br>0.03313

Table G: Correspondent statistical details for Fig 4 "Decreased presence of tubulin-GFP at axonal microtubules in a pulse chase experiment suggests changes in MT turnover with age".

	EB1 in axons	
Age (days)	**3-6 days**	**28-35 days**
Genotype	***WT***	
Number of values (medullas)<br>Number specimens<br>Mean<br>Std. Deviation<br>Std. Error of Mean	26<br>15<br>1.000<br>0.2688<br>0.05271	29<br>17<br>0.8534<br>0.3349<br>0.06219
	**Tau in axons**	
Age (days)	**5-7 days**	**33-35 days**
Genotype	**Tau^{wee304}/+**	
Number of values (medullas)<br>Number specimens<br>Mean<br>Std. Deviation<br>Std. Error of Mean	46<br>25<br>29.21<br>18.85<br>2.779	41<br>22<br>13.10<br>13.62<br>2.127
	**Total EB1**	
Age (days)	**3-9 days**	**28-34 days**
Genotype	***WT***	
Number of values	6	6

Mean<br>Std. Deviation<br>Std. Error of Mean	1.024<br>0.1688<br>0.06890	1.080<br>0.4631<br>0.1891
	**Total Tau**	
Age (days)	**6-9 days**	**30-35 days**
Genotype	***WT***	
Number of values<br>Mean<br>Std. Deviation<br>Std. Error of Mean	10<br>0.9664<br>0.1582<br>0.05002	11<br>0.6505<br>0.2655<br>0.08005
	**EB1-MT enrichment assay**	
Age (days)	**2-6 days**	**28-31 days**
Genotype	***WT***	
Number of values<br>**Mean MT pellet**<br>Std. Deviation<br>Std. Error of Mean	9<br>1.110<br>0.3633<br>0.1211	10<br>0.5600<br>0.2073<br>0.06556
**Mean supernatant**	1.062	1.069
Std. Deviation	0.1577	0.2732
Std. Error of Mean	0.04988	0.08638
	**Tau-MT enrichment assay**	
Age (days)	**2-6 days**	**28-31 days**
Genotype	***WT***	
Number of values<br>**Mean MT pellet**<br>Std. Deviation<br>Std. Error of Mean	10<br>1.193<br>0.3165<br>0.1001	10<br>0.8550<br>0.2171<br>0.06866
**Mean supernatant**	1.062	1.069
Std. Deviation	0.1577	0.2732
Std. Error of Mean	0.04988	0.08638

Table H: Correspondent statistical details for Fig 5 “The function of EB1 and Tau is altered during ageing”.

Genotype	*W*^{1118}*;UAS-GFP-α-tubulin84B, GMR31F10-Gal4/+*	*W*^{1118}*;UAS-GFP-α-tubulin84B, GMR31F10-Gal4/UAS-**Tau***^{RNI}	*W*^{1118}*;UAS-myr-tdTomato /+ ; UAS-GFP-α-tubulin84B, GMR31F10-Gal4/+*	*W*^{1118}*;UAS-myr-tdTomato /UAS-**Shot***^{RNI} *; UAS-GFP-α-tubulin84B, GMR31F10-Gal4/+*
Age (days)	**30-37**	**30-37**	**30-33**	**30-33**
	**Axon swellings**			
Number of values (axonal bundle segment)	511	502	310	295

Number specimens	19	17	14	12
Mean	1.000	1.278	1.001	2.109
Std. Deviation	1.198	1.389	1.440	2.566
Std. Error of Mean	0.05302	0.06198	0.08179	0.1494
	**MT unbundling**			
Number of values (axonal bundle segment)	511	502	310	289
Number specimens	19	17	14	12
Mean	1.000	3.885	0.9999	4.704
Std. Deviation	5.597	12.42	2.810	8.794
Std. Error of Mean	0.2476	0.5542	0.1596	0.5173
	**MT breaks**			
Number of values (axonal bundle segment)	511	502	311	289
Number specimens	19	17	14	12
Mean	1.000	1.421	0.9967	1.756
Std. Deviation	3.280	3.686	2.720	3.848
Std. Error of Mean	0.1451	0.1645	0.1543	0.2229
Genotype	*W^{1118};UAS-myristoylated-Tomato /+ ; UAS-GFP-α-tubulin84B, GMR31F10-Gal4/+*	*W^{1118};UAS-myristoylated-Tomato / UAS-**Shot^{RNI}** ; UAS-GFP-α-tubulin84B, GMR31F10-Gal4/UAS-**Tau^{RNI}***	*W^{1118};UAS-myristoylated-Tomato /+ ; UAS-GFP-α-tubulin84B, GMR31F10-Gal4/+*	*W^{1118};UAS-myristoylated-Tomato **/EB1 Df**; UAS-GFP-α-tubulin84B, GMR31F10-Gal4/ **UAS-EB1^{RNI}***
Age (days)	**28-32**	**28-32**	**25-28**	**25-28**
	**Axon swellings**			
Number of values (axonal bundle segment)	340	446	464	457
Number specimens	19	18	14	14
Mean	1.000	3.366	1.000	2.907
Std. Deviation	1.806	3.892	2.351	4.458
Std. Error of Mean	0.09792	0.1843	0.1091	0.2085
	**MT unbundling**			
Number of values (axonal bundle segment)	340	442	465	461
Number specimens	19	18	14	14
Mean	1.000	5.103	1.000	2.143
Std. Deviation	3.237	8.556	3.639	6.860
Std. Error of Mean	0.1756	0.4070	0.1687	0.3195
	**MT breaks**			

Number of values (axonal bundle segment)	344	437	466	465
Number specimens	19	18	14	14
Mean	0.9999	3.173	1.000	2.795
Std. Deviation	3.013	6.000	3.267	5.571
Std. Error of Mean	0.1622	0.2870	0.1514	0.2583

Table I: Correspondent statistical details for Fig 6 "Knocking down of Eb1, Tau and Shot exacerbates age-related MT decay and ageing hallmarks".

Genotype	*W^{1118};UAS-myr-tdTomato /+ ; UAS-GFP-α-tubulin84B, GMR31F10-Gal4/+*	*W^{1118};UAS-myr-tdTomato / UAS-**$Shot^{RNI}$** ; UAS-GFP-α-tubulin84B, GMR31F10-Gal4/UAS-**Tau^{RNI}***	*W^{1118};UAS-myr-tdTomato /+ ; UAS-GFP-α-tubulin84B, GMR31F10-Gal4/+*	*W^{1118};UAS-myr-tdTomato /+; UAS-GFP-α-tubulin84B, GMR31F10-Gal4/ **UAS-mcherry-EB1***
Age (days)	**28-32**	**28-32**	**25-28**	**25-28**
	**Synaptic MTs**			
Number of values (terminals)	340	446	219	184
Number specimens	14	20	17	14
Mean	5.703	2.574	4.909	6.658
Std. Deviation	2.116	1.598	1.668	1.761
Std. Error of Mean	0.1614	0.1027	0.1127	0.1298
	**Terminal Morphology**			
Number of values (terminals)	1143	1220	2006	1770
Number of normal	1028	663	1506	1623
Number of swollen+broken	115	557	500	147
Number specimens	14	20	17	14
Genotype	*W^{1118};UAS-myr-tdTomato /+ ; UAS-GFP-α-tubulin84B, GMR31F10-Gal4/+*	*W^{1118};UAS-myr-tdTomato / +; GMR31F10-Gal4/ **UAS-$Shot^{EGC}$-GFP***		
Age (days)	**27-31**	**27-31**		
	**Synaptic MTs**			
Number of values (terminals)	194	146		
Number specimens	15	13		
Mean	5.701	6.411		
Std. Deviation	2.057	2.237		
Std. Error of Mean	0.1477	0.1851		
	**Terminal Morphology**			

Number of values (terminals)	340	442		
Number of normal	725	654		
Number of swollen+broken	188	78		
Number specimens	15	13		

Table J: Correspondent statistical details for S9 Fig "The deterioration of synaptic terminals during ageing can be exacerbated or rescued by altering the function of MT regulators"

Genotype	*W1118;UAS-GFP-α-tubulin84B, GMR31F10-Gal4/+*	*W1118;UAS-GFP-α-tubulin84B, GMR31F10-Gal4/UAS-**Tau**RNI*	*W1118;UAS-myr-tdTomato /+ ; UAS-GFP-α-tubulin84B, GMR31F10-Gal4/+*	*W1118;UAS-myr-tdTomato /UAS-**Shot**RNI ; UAS-GFP-α-tubulin84B, GMR31F10-Gal4/+*
Age (days)	**3-8**	**3-8**	**5-7**	**5-7**
	**Axon swellings**			
Number of values (axonal bundle segment)	567	598	73	311
Number specimens	19	24	6	8
Mean	1.000	1.102	0.9999	1.056
Std. Deviation	1.766	2.280	1.948	2.154
Std. Error of Mean	0.07417	0.09325	0.2280	0.1221
	**MT unbundling**			
Number of values (axonal bundle segment)	478	598	73	319
Number specimens	19	24	6	8
Mean	1.000	0.1802	1.000	0.5035
Std. Deviation	4.789	1.963	3.714	2.668
Std. Error of Mean	0.2191	0.08029	0.4346	0.1494
	**MT breaks**			
Number of values (axonal bundle segment)	478	597	73	321
Number specimens	19	24	6	8
Mean	1.000	1.021	0.9999	0.6822
Std. Deviation	2.054	2.118	4.863	4.023
Std. Error of Mean	0.09393	0.08668	0.5692	0.2245
Genotype	*W1118;UAS-myristoylated-Tomato /+ ; UAS-GFP-α-tubulin84B,*	*W1118;UAS-myristoylated-Tomato / UAS-**Shot**RNI ; UAS-GFP-α-tubulin84B,*	*W1118;UAS-myristoylated-Tomato /+ ; UAS-GFP-α-tubulin84B,*	*W1118;UAS-myristoylated-Tomato **/EB1 Df**; UAS-GFP-α-tubulin84B,*

	GMR31F10-Gal4/+	*GMR31F10-Gal4/UAS-**Tau**^{RNI}*	*GMR31F10-Gal4/+*	*GMR31F10-Gal4/ **UAS-EB1**^{RNI}*
Age (days)	**3-7**	**3-7**	**3-8**	**3-8**
	**Axon swellings**			
Number of values (axonal bundle segment)	383	450	329	359
Number specimens	17	18	13	17
Mean	0.9973	0.7563	1.000	0.7590
Std. Deviation	1.065	1.080	1.366	1.176
Std. Error of Mean	0.05449	0.05093	0.07533	0.06234
	**MT unbundling**			
Number of values (axonal bundle segment)	382	450	329	357
Number specimens	17	18	13	17
Mean	1.000	1.313	0.9999	0.4280
Std. Deviation	8.320	9.268	5.362	4.219
Std. Error of Mean	0.4257	0.4369	0.2956	0.2233
	**MT breaks**			
Number of values (axonal bundle segment)	382	450	329	356
Number specimens	17	18	13	17
Mean	1.000	1.352	1.000	0.7732
Std. Deviation	8.504	10.80	2.071	1.837
Std. Error of Mean	0.4351	0.5093	0.1142	0.09734

Table K: Correspondent statistical details for S10 Fig “MT decay and ageing hallmarks are not present in young specimens with EB1, Tau and Shot knockdowns”.

Genotype	*W^{1118};UAS-myr-tdTomato /+ ; UAS-GFP-α-tubulin84B, GMR31F10-Gal4/+*	*W^{1118};UAS-myr-tdTomato /+; UAS-GFP-α-tubulin84B, GMR31F10-Gal4/ **UAS-mcherry-EB1***	*W^{1118};UAS-myr-tdTomato /+ ; UAS-GFP-α-tubulin84B, GMR31F10-Gal4/+*	*W^{1118};UAS-myr-tdTomato / +; GMR31F10-Gal4/ **UAS-Shot^{EGC}-GFP***
Age (days)	**28-30**	**28-30**	**27-31**	**27-31**
	**MT unbundling**			
Number of values (axonal bundle segment)	377	308	382	303
Number specimens	17	14	14	13
Mean	0.9999	0.4584	1.000	0.03287
Std. Deviation	2.579	1.938	2.825	0.4039

Std. Error of Mean	0.1328	0.1104	0.1445	0.02321
	**MT breaks**			
Number of values<br>Number specimens<br>Mean<br>Std. Deviation<br>Std. Error of Mean	371<br>17<br>0.9421<br>2.996<br>0.1555	302<br>14<br>0.4247<br>1.779<br>0.1024	238<br>14<br>0.9998<br>2.844<br>0.1844	241<br>13<br>0.4481<br>1.744<br>0.1123
	**Axon Swellings**			
Number of values (axonal bundle segment)<br>Number specimens<br>Mean<br>Std. Deviation<br>Std. Error of Mean	375<br>17<br>0.9999<br>1.499<br>0.07742	301<br>14<br>0.6126<br>1.076<br>0.06201	381<br>14<br>1.000<br>1.828<br>0.09366	303<br>13<br>0.01488<br>0.1829<br>0.01051
	**MT bundle diameter**			
Age (days)	**25-28**	**25-28**	**29-30**	**29-30**
Number of values (axonal bundle segment)<br>Number specimens<br>Mean<br>Std. Deviation<br>Std. Error of Mean	31<br>5<br>1.000<br>0.2152<br>0.03865	40<br>7<br>1.261<br>0.3327<br>0.05261	145<br>10<br>1.000<br>0.2281<br>0.01894	129<br>10<br>1.377<br>0.2584<br>0.02275
	**Axon diameter**			
Age (days)	**35-38**	**35-38**	**27-31**	**27-31**
Number of values (axons)<br>Number specimens<br>Mean<br>Std. Deviation<br>Std. Error of Mean	291<br>16<br>1.000<br>0.1673<br>0.009805	165<br>12<br>1.137<br>0.1976<br>0.01539	207<br>13<br>1.000<br>0.1789<br>0.01243	242<br>12<br>1.468<br>0.2858<br>0.01837

Table L: Correspondent statistical details for Fig 7 "Expression of EB1 and Shot^{EGC} ameliorates axonal ageing phenotypes"

Genotype	*W^{1118};tub-Gal80^{ts}/ UAS-myr-tdTomato; UAS-GFP-α-tubulin84B, GMR31F10-Gal4/+*	*W^{1118};tub-Gal80^{ts}/ UAS-myr-tdTomato; UAS-GFP-α-tubulin84B, GMR31F10-Gal4/* ***UAS-mcherry-EB1***	*W^{1118};tub-Gal80^{ts}/ UAS-myr-tdTomato; UAS-GFP-α-tubulin84B, GMR31F10-Gal4/+*	*W^{1118};tub-Gal80^{ts}/ UAS-myr-tdTomato; GMR31F10-Gal4/* ***UAS-Shot^{EGC}-GFP***
Age (days)	**27-31**	**27-31**	**27-31**	**27-31**
	**MT unbundling**			

Number of values (axonal bundle segment)	721	449	721	697
Number specimens	34	24	26	23
Mean	1.000	0.7952	1.000	0.3564
Std. Deviation	4.104	3.441	4.104	2.440
Std. Error of Mean	0.1529	0.1624	0.1529	0.09242
	**MT breaks**			
Number of values (axonal bundle segment)	721	449	721	697
Number specimens	34	24	26	23
Mean	1.000	0.6500	1.000	0.2334
Std. Deviation	3.441	2.785	3.441	1.849
Std. Error of Mean	0.1282	0.1314	0.1282	0.07003
	**Axon Swellings**			
Number of values (axonal bundle segment)	721	449	721	697
Number specimens	34	24	26	23
Mean	1.000	0.6801	1.000	0.2759
Std. Deviation	1.148	0.9823	1.148	0.6025
Std. Error of Mean	0.04274	0.04636	0.04274	0.02282

Table M: Correspondent statistical details for S14 Fig "Post-developmental expression of Shot^{EGC} but not of EB1 is sufficient to rescue all axonal ageing phenotypes".

Genotype	*W^{1118};elav-GS/+*	*W^{1118}; elav-GS/**UAS-Shot^{EGC}-GFP***	*W^{1118}; elav-GS/**UAS-EB1-GFP***	*W^{1118};elav-GS/+*	*W^{1118}; elav-GS/**UAS-Shot^{EGC}-GFP***	*W^{1118}; elav-GS/**UAS-EB1-GFP***
			**Females**			
Age (days)	**6-10**			**22-25**		
Number specimens	**- RU486**<br>189<br>**+RU486**<br>182	**- RU486**<br>104<br>**+RU486**<br>111	**- RU486**<br>55<br>**+RU486**<br>96	**- RU486**<br>137<br>**+RU486**<br>140	**- RU486**<br>60<br>**+RU486**<br>88	**- RU486**<br>54<br>**+RU486**<br>98
Mean	**- RU486**<br>1<br>**+RU486**<br>1.188	**- RU486**<br>1<br>**+RU486**<br>0.9881	**- RU486**<br>1<br>**+RU486**<br>1.164	**- RU486**<br>1<br>**+RU486**<br>1.137	**- RU486**<br>1<br>**+RU486**<br>1.609	**- RU486**<br>1<br>**+RU486**<br>1.253
Std. Deviation	**- RU486**<br>0.6845<br>**+RU486**<br>0.6559	**- RU486**<br>0.6197<br>**+RU486**<br>0.5638	**- RU486**<br>0.598<br>**+RU486**<br>0.7889	**- RU486**<br>0.8816<br>**+RU486**<br>1.087	**- RU486**<br>0.8452<br>**+RU486**<br>0.8657	**- RU486**<br>0.7896<br>**+RU486**<br>0.7036
Std. Error of Mean	**- RU486**<br>0.04979<br>**+RU486**	**- RU486**<br>0.06076<br>**+RU486**	**- RU486**<br>0.08073<br>**+RU486**	**- RU486**<br>0.07532<br>**+RU486**	**- RU486**<br>0.04979<br>**+RU486**	**- RU486**<br>0.1075<br>**+RU486**

	0.04862	0.05352	0.08052	0.1091	0.09228	0.04862
**Males**						
Age (days)	**6-10**			**22-25**		
Number specimens	**- RU486**<br>180<br>**+RU486**<br>185	**- RU486**<br>105<br>**+RU486**<br>85	**- RU486**<br>42<br>**+RU486**<br>90	**RU486**<br>187<br>**+RU486**<br>180	**- RU486**<br>92<br>**+RU486**<br>89	**- RU486**<br>56<br>**+RU486**<br>109
Mean	**- RU486**<br>1<br>**+RU486**<br>0.9731	**- RU486**<br>1<br>**+RU486**<br>1.056	**- RU486**<br>1<br>**+RU486**<br>0.7269	**- RU486**<br>1<br>**+RU486**<br>0.8237	**- RU486**<br>1<br>**+RU486**<br>1.033	**- RU486**<br>1<br>**+RU486**<br>0.9327
Std. Deviation	**- RU486**<br>0.7819<br>**+RU486**<br>0.8123	**- RU486**<br>0.6893<br>**+RU486**<br>0.7375	**- RU486**<br>0.3029<br>**+RU486**<br>0.4873	**- RU486**<br>0.7994<br>**+RU486**<br>0.7501	**- RU486**<br>0.7166<br>**+RU486**<br>0.6547	**- RU486**<br>0.3207<br>**+RU486**<br>0.3878
Std. Error of Mean	**- RU486**<br>0.05828<br>**+RU486**<br>0.05972	**- RU486**<br>0.06727<br>**+RU486**<br>0.08000	**- RU486**<br>0.04674<br>**+RU486**<br>0.05136	**- RU486**<br>0.05846<br>**+RU486**<br>0.05591	**- RU486**<br>0.07471<br>**+RU486**<br>0.06940	**- RU486**<br>0.04285<br>**+RU486**<br>0.03714

TableN: Correspondent statistical details for Fig 8 "Expression of Shot ^{EGC} and EB1 in adult *Drosophila* using the Geneswitch system improves locomotion of aged flies ".

Genotype	*W^{1118};tub-Gal80^{ts}/ +; elav-Gal4/+*	*W^{1118};tub-Gal80^{ts}/ +; elav-Gal4/**UAS-mcherry-EB1***	*W^{1118};tub-Gal80^{ts}/ +; elav-Gal4/**UAS-Shot^{EGC}-GFP***	*W^{1118};tub-Gal80^{ts}/ +; elav-Gal4/+*	*W^{1118};tub-Gal80^{ts}/ +; elav-Gal4/**UAS-mcherry-EB1***	*W^{1118};tub-Gal80^{ts}/ +; elav-Gal4/**UAS-Shot^{EGC}-GFP***
Age (days)	**4-5**	**4-5**	**4-5**	**25-26**	**25-26**	**25-26**
Number specimens	239	162	146	230	143	168
Mean	15.00	10.02	14.71	1.004	0.9091	2.012
Std. Deviation	4.733	5.817	4.489	1.884	1.524	2.189
Std. Error of Mean	0.3061	0.4570	0.3715	0.1242	0.1274	0.1689

Table O: Correspondent statistical details for S15 Fig "The decline in locomotion of aged flies improves upon adult expression of Shot^{EGC}".

	**Synaptic MTs**	
	**Ice anaesthesia**	**CO2 anaesthesia**
Genotype	*W^{1118};tub-Gal80^{ts}/ UAS-myr-tdTomato; UAS-GFP-α-tubulin84B, GMR31F10-Gal4/+*	
Number of values (axons)	250	224
Number specimens	15	13
Mean	1	0.907
Std. Deviation	0.3017	0.2944
Std. Error of Mean	0.01908	0.01967

Table P: Correspondent statistical details for S16 Fig "Comparison of the effect on T1 MTs of Ice/cold or CO2 based anaesthesia treatments ".

	Gapdh/total protein	
Age (days)	**4 to 9 days 29^{0}C**	**29 to 32 days 29^{0}C**
Genotype	*W^{1118}*	
Number of values (axons)	13	13
Mean	1.02	1.03
Std. Deviation	0.15	0.30
Std. Error of Mean	0.04	0.08
	**Actin/total protein**	
Age (days)	**4 to 9 days 29^{0}C**	**29 to 32 days 29^{0}C**
Genotype	*W^{1118}*	
Number of values (axons)	9	9
Mean	0.941	0.7595
Std. Deviation	0.128	0.2912
Std. Error of Mean	0.042	0.09

Table Q: Correspondent statistical details for S17 Fig "The levels of loading control proteins Gapdh and Actin do not change during ageing ".
